# SARS-CoV-2 Transmission in Alberta, British Columbia, and Ontario, Canada, January 2020–January 2022

**DOI:** 10.3201/eid3005.230482

**Published:** 2024-05

**Authors:** Aubrey D. Kehoe, Arshpreet Kaur Mallhi, Charles R. Barton, Hunter M. Martin, Christopher M. Turner, Xinyi Hua, Kin On Kwok, Gerardo Chowell, Isaac Chun-Hai Fung

**Affiliations:** Georgia Southern University Jiann-Ping Hsu College of Public Health, Statesboro, Georgia, USA (A.D. Kehoe, A.K. Mallhi, C.R. Barton, H.M. Martin, C.M. Turner, X. Hua, I.C.-H. Fung);; Chinese University of Hong Kong JC School of Public Health and Primary Care, Hong Kong (K.O. Kwok);; Georgia State University School of Public Health, Atlanta, Georgia, USA (G. Chowell)

**Keywords:** COVID-19, SARS-CoV-2, viruses, coronavirus disease, respiratory infections, zoonoses, epidemiology, infectious diseases, reproduction number, transmission, Canada

## Abstract

We estimated COVID-19 transmission potential and case burden by variant type in Alberta, British Columbia, and Ontario, Canada, during January 23, 2020–January 27, 2022; we also estimated the effectiveness of public health interventions to reduce transmission. We estimated time-varying reproduction number (R_t_) over 7-day sliding windows and nonoverlapping time-windows determined by timing of policy changes. We calculated incidence rate ratios (IRRs) for each variant and compared rates to determine differences in burden among provinces. R_t_ corresponding with emergence of the Delta variant increased in all 3 provinces; British Columbia had the largest increase, 43.85% (95% credible interval [CrI] 40.71%–46.84%). Across the study period, IRR was highest for Omicron (8.74 [95% CrI 8.71–8.77]) and burden highest in Alberta (IRR 1.80 [95% CrI 1.79–1.81]). Initiating public health interventions was associated with lower R_t_ and relaxing restrictions and emergence of new variants associated with increases in R_t_.

Emergence of SARS-CoV-2 in 2019 resulted in the COVID-19 pandemic (*1*). Since then, the virus has mutated into multiple variants. Each variant differed in transmissibility and severity of health outcomes, leading to differences in incident case and death counts in different regions (*2*,*3*). COVID-19 transmission rates vary depending on multiple factors, including public health policies and characteristics of emerging variants. Interventions such as working from home, school closures, face mask mandates, and vaccination were implemented as efforts to control the pandemic. 

Various studies have evaluated the effectiveness of pandemic interventions in Canada (*4*–*7*). Indoor mask mandates were found to be associated with weekly decreases of 22% in case counts (*4*). Physical distancing was found to effectively mitigate COVID-19 spread in Ontario (*5*). The mitigating effect of stay-at-home policies on COVID-19 spread in Toronto was also reported (*7*). Most of the previous studies conducted on populations in Canada have evaluated COVID-19 preventive measures in a single city or province or evaluated only 1 or 2 preventive measures. Limited research has been conducted comparing the additive mitigating effects of prevention and control measures among major provinces in Canada. With the exception of a few studies focused mainly on COVID-19 vaccine effectiveness, little research has examined the effects of initiating public health interventions or the emergence of new variants on COVID-19 transmission potential and disease burden in Canada (*8*,*9*). Moreover, differences in time-varying reproduction number (R_t_) and incidence rate ratio (IRR) associated with specific SARS-CoV-2 variants have yet to be explored among populations in Canada. A 2020 study calculated IRR to compare disease burden across 3 provinces (*10*); however, variant-specific comparisons have yet to be explored.

We addressed 2 of those research questions in this study. First, we investigated transmission potential and case burden associated with wild-type, Alpha, Delta, and Omicron SARS-CoV-2 variants in Alberta, British Columbia, and Ontario. Second, we investigated the effectiveness of public health interventions to reduce SARS-CoV-2 transmission potential in those 3 provinces. The Georgia Southern University institutional review board determined that this project (H20364) did not involve human subjects under the G8 exemption category of Code of Federal Regulations Title 45, Part 46. 

## Methods

### Data Acquisition

We downloaded and analyzed publicly available COVID-19 case data from Ontario, British Columbia, and Alberta. Data from Ontario, which is divided into 6 subprovincial public health regional areas—Central, East, North-East, North-West, Toronto, and West—came from 1,033,294 cases reported during January 23, 2020–January 27, 2022 (Appendix Table 1) (*11*,*12*). Data from British Columbia, which is divided into 5 subprovincial regional health authorities—Fraser, Interior, Northern, Vancouver Coastal, and Vancouver Island—came from 320,540 cases reported during January 29, 2020–January 27, 2022 (Appendix Table 2) (*13*,*14*). Data from Alberta, which is divided into 5 subprovincial health services zones—Calgary, Central, Edmonton, North, and South—came from 487,045 cases during March 6, 2020–January 27, 2022 (Appendix Table 3) (*15*,*16*). 

We obtained information from the government of Canada, the Canadian Institute for Health Information, and the Upper Canada District School Board websites on COVID-19 policies, including descriptions and dates of implementation and relaxation (Appendix Table 4) (*17*–*19*). In addition to the policy timepoints, we recorded dates of incidence spikes because of Delta and Omicron variants, chosen to represent the beginning of the time period when emergence of the variant resulted in increases in case counts and R_t_ (Appendix Table 4). Through this analysis, we aimed to examine the effect on R_t_ of mitigation policies and emergence of highly transmissible variants. We used the Government of Canada website (*20*) as the source for data on COVID-19 variants, including weekly percentages for each identified variant among all cases for which samples were sequenced. Percentages represented the weekly distribution of confirmed cases by variant. We multiplied daily case counts by weekly variant percentages to estimate daily cases attributed to each variant (Appendix). 

### Statistical Analysis

We presented epidemic curves of weekly incident case count by province according to age group, sex, and public health district and log_10_-transformed monthly cumulative case counts in maps at the subprovincial level. Basic reproduction number (R_0_) is the average number of secondary cases of an infectious disease infected by the primary case-patient in a totally susceptible population (*21*). R_t_, which is the expected number of new cases attributed to 1 infected person, changes over time because of public health interventions and the changing proportion of the population with immunity against the infection. An R_t_ >1 indicates epidemic growth, whereas R_t_ <1 indicates epidemic decline (*22*). 

We used the R package EpiEstim (The R Project for Statistical Computing, https://cran.r-project.org/web/packages/EpiEstim/index.html) to estimate R_t_ from incident COVID-19 case counts in Ontario, British Columbia, and Alberta. R_t_ is estimated by calculating the ratio between the number of incident cases at time t and the total infectiousness of all infected persons at that same time (*23*,*24*). We specified a mean serial interval (SI) distribution of 4.60 d (SD 5.55 d) for the analysis (*25*). We generated 2 forms of R_t_: a 7-day sliding-window R_t_ and a policy change R_t_ over nonoverlapping time-windows. The 7-day sliding-window R_t_ estimate is an average of daily R_t_ estimates over a sliding-window time period comprising the current day and the 6 immediately previous days; therefore, each daily R_t_ estimate is included in 7 different estimates. The policy change R_t_ is an average of daily R_t_ estimates over a nonoverlapping time-window in which each daily R_t_ is included in only 1 estimate. We conducted a sensitivity analysis of provincial-level R_t_ estimates that incorporated uncertainty associated with underreporting of infections. We calculated R_t_ using estimated infection counts assuming averages from one sensitivity analysis using a multiplier of 4 (*26*) and another using a multiplier of 11 (*27*) infected persons reported as case-patients. 

To calculate incidence rates, we divided cumulative case counts by observed person-time data and multiplied by 100,000. Person-time observed was the product of the estimated population size in 2021 (*28*) and the number of days observed by province, variant, or both. We stratified incidence rates over the entire study period and IRR by sex, age, and variant for each province (Appendix). We analyzed data using R version 4.2.2 (https://cran.r-project.org/bin/windows/base/old/4.2.2). 

## Results 

### Descriptive Statistics and R_t_ by Province

#### Ontario

**Figure 1:**
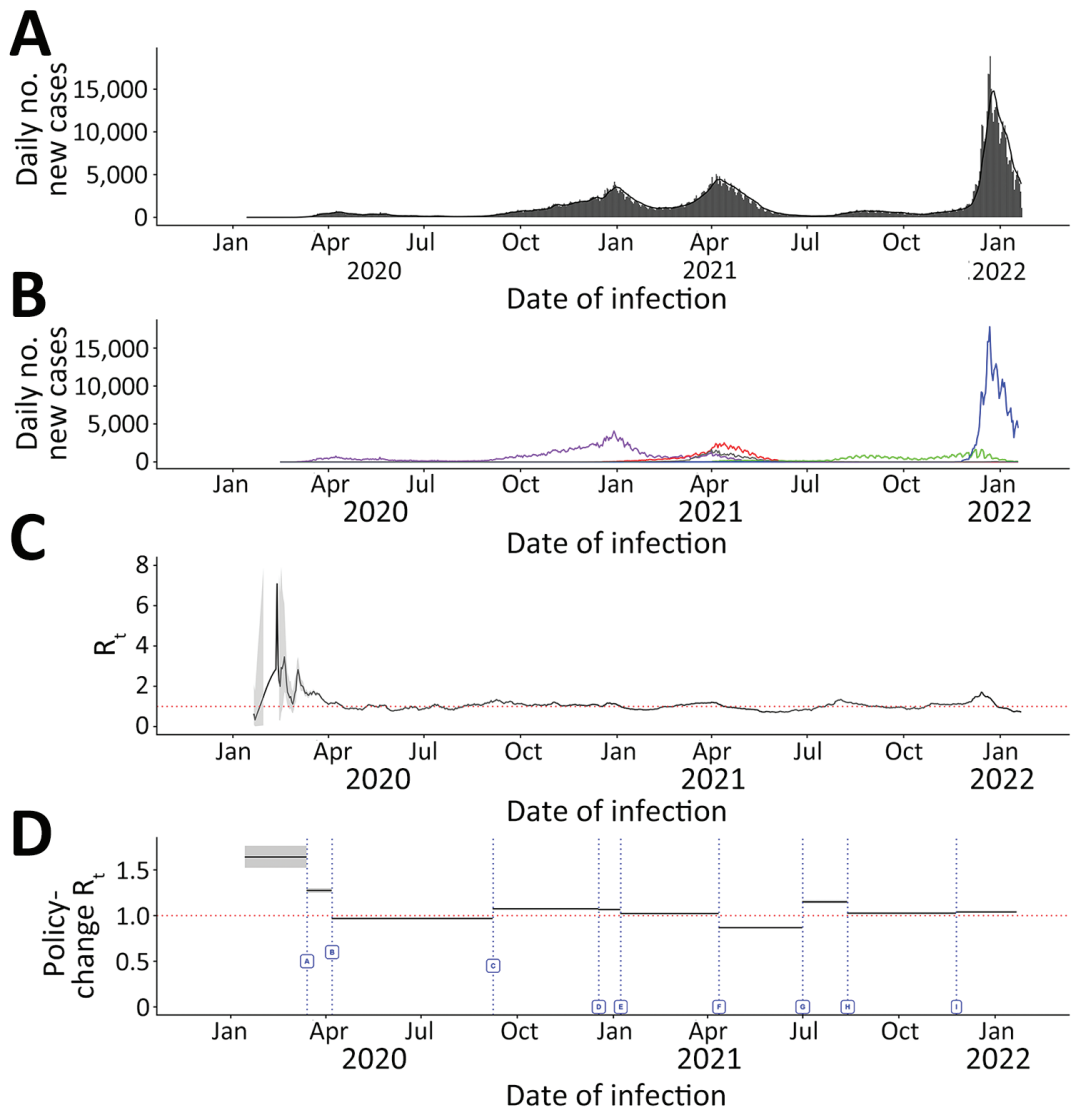
Daily incident COVID-19 case count and 7-day moving average R_t_, by date of infection and policy-change R_t_ or new variants by initiation dates, Ontario, Canada, January 23, 2020–January 27, 2022. Dates are assumed infection dates (i.e., report date minus 9 days); red dotted lines in panels C and D indicate R_t_ = 1. A, B) Incident case count by date of infection for all variants combined (A) and by variant type (B). Colors indicate COVID-19 variants: purple, wild-type; red, Alpha; green, Delta; blue, Omicron; and gray, other variants. C) Seven-day moving average R_t_ by date of infection. D) Policy-change R_t_. Policy changes or detection of new variations by dates of initiation: A, school closure (March 14, 2020); B, recommendation for use of face masks (April 7, 2020); C, phased school reopening (September 8, 2020); D, priority populations vaccination rollout (December 18, 2020); E, partial school reopening (January 8, 2021); F, school closure (April 12, 2021); G, increase in cases because of Delta COVID-19 variant (July 1, 2021); H, requirement of vaccination for federal workers (August 13, 2021); I, increase in cases because of the Omicron COVID-19 variant (November 25, 2021).

Ontario experienced 3 major pandemic waves during our study period; more substantial case burdens occurred in metropolitan areas. The first surge, which peaked in December 2020, was attributed to wild-type virus; the second surge, peaking in April 2021, was mainly from Alpha; and the third, peaking in December 2021, was mainly from Omicron (Figure 1; Appendix Figures 1, 2). Unlike British Columbia and Alberta, Ontario did not experience a surge because of Delta; when the Delta variant became the dominant strain in June 2021, the daily incident case count remained stable and Delta did not cause any major surges during June–December 2021, after which the Omicron variant became dominant (Figure 1). Corresponding to the sustained transmission presented in the epidemic curves, R_t_ was >1 during August–September 2020, February–April 2021, July–August 2021, and November–December 2021. Except for February and March 2020, R_t_ was not >2. Sensitivity analysis using estimated infection counts generated similar R_t_ trends but with a wider 95% credible interval (CrI) that included 1 throughout much of the study period time frame (Appendix Figure 3). 

The policy change R_t_ fluctuated throughout the study period with implementation of policies and emergence of new variants (Figure 1; Table 1). Before policies were implemented, a median R_t_ of 1.64 (95% CrI 1.52–1.76) was observed. Initiating school closures in March 2020 (policy A), recommendation of face coverings (policy B), partial school reopening after Christmas/New Year holiday break (policy E), school closures in April 2021 (policy F), and required vaccination for government workers (policy H) were associated with decreased median R_t_, whereas relaxation of policies such as phased school reopening (policy C) were associated with increased median R_t_. Emergence of Delta (denoted by G) and Omicron variants (denoted by I) were associated with increased median R_t_. Implementation of vaccination rollout for priority populations (policy D) was not associated with any change in median R_t_. In Ontario, the most significant increase in R_t_, 32.63% (95% CrI 30.22%–35.07%), was observed after the Delta variant emerged. In contrast, the greatest decrease in R_t_, −24.01% (95% CrI −25.60% to −22.30%), was observed after the face covering recommendation took effect. The lowest median R_t_, 0.87 (95% CrI, 0.86–0.87), during the study period was observed after school closures in April 2021. 

**Table 1:** Estimates for median R_t_ and percentage change in R_t_ after implementation of policies and the emergence of COVID-19 variant in 3 provinces of Canada (Ontario, British Columbia, and Alberta) during January 23, 2020–January 27, 2022*

Policy/variant	Median R_t_ (95% credible interval)	% Change (95% credible interval)
Ontario		
Policy label/emergent variant†		
Before A	1.641 (1.523–1.764)	Not applicable
A→B	1.275 (1.249–1.301)	−22.426 (−28.083 to −16.190)
B→C	0.968 (0.958–0.979)	−24.008 (−25.597 to −22.302)
C→D	1.075 (1.069–1.081)	10.964 (9.753–12.243)
D→E	1.066 (1.058–1.074)	−0.828 (−1.733–0.131)
E→F	1.023 (1.019–1.028)	−4.021 (−4.854 to −3.145)
F→G	0.867 (0.862–0.872)	−15.232 (−15.841 to −14.556)
G→H	1.150 (1.130–1.170)	32.629 (30.223–35.067)
H→I	1.027 (1.019–1.035)	−10.789 (−12.515 to −9.077)
After I	1.040 (1.036–1.043)	1.296 (0.440–2.094)
British Columbia		
Policy label/emergent variant†		
Before A	1.641 (1.505–1.784)	Not applicable
A→B	0.945 (0.889–1.004)	−42.558 (−48.205 to −35.902)
B→C	1.069 (1.043–1.096)	13.263 (5.929–21.413)
C→D	1.042 (1.032–1.052)	−2.577 (−5.004–0.087)
D→E	1.007 (0.989–1.026)	−3.333 (−5.309 to −1.139)
E→F	1.036 (1.028–1.044)	2.802 (0.796–4.972)
F→G	0.862 (0.852–0.873)	−16.787 (−17.974 to −15.455)
G→H	1.240 (1.218–1.262)	43.849 (40.706–46.835)
H→I	0.984 (0.976–0.992)	−20.725 (−22.297 to −19.154)
After I	1.075 (1.069–1.082)	9.364 (8.235–10.445)
Alberta		
Policy label/emergent variant†		
Before A	1.815 (1.614–2.031)	Not applicable
A→B	1.187 (1.130–1.245)	−34.710 (−42.227 to −25.800)
B→C	1.021 (1.004–1.038)	−13.898 (−17.841 to −9.344)
C→D	1.056 (1.049–1.063)	3.467 (1.733–5.358)
D→E	0.945 (0.932–0.958)	−10.522 (−11.882 to −9.013)
E→F	1.049 (1.040–1.057)	10.925 (9.174–12.810)
F→G	0.906 (0.898–0.913)	−13.657 (−14.622 to −12.595)
G→H	1.270 (1.246–1.294)	40.273 (37.563–42.923)
H→I	0.989 (0.982–0.995)	−22.172 (−23.655 to −20.689)
After I	1.101 (1.095–1.106)	11.385 (10.452–12.280)

**Figure 2:**
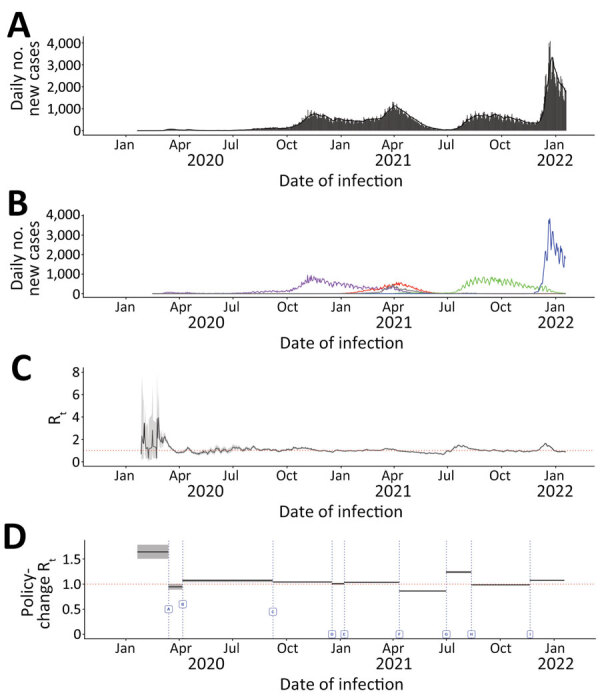
Daily incident COVID-19 case count and 7-day moving average R_t_, by date of infection and policy-change R_t_ or new variants by initiation dates, British Columbia, Canada, January 23, 2020–January 27, 2022. Dates are assumed infection dates (i.e., report date minus 9 days); red dotted lines in panels C and D indicate R_t_ = 1. A, B) Incident case count by date of infection for all variants combined (A) and by variant type (B). Colors indicate COVID-19 variants: purple, wild-type; red, Alpha; green, Delta; blue, Omicron; and gray, other variants. C) Seven-day moving average R_t_ by date of infection. D) Policy-change Rt. Policy changes or detection of new variants by dates of initiation: A, school closure (March 14, 2020); B, recommendation for use of face masks (April 7, 2020); C, phased school reopening (September 8, 2020); D, priority populations vaccination rollout (December 18, 2020); E, partial school reopening (January 8, 2021); F, school closure (April 12, 2021); G, increase in cases because of Delta COVID-19 variant (July 1, 2021); H, requirement of vaccination for federal workers (August 13, 2021); I, increase in cases because of the Omicron COVID-19 variant (November 21, 2021).

**Figure 3:**
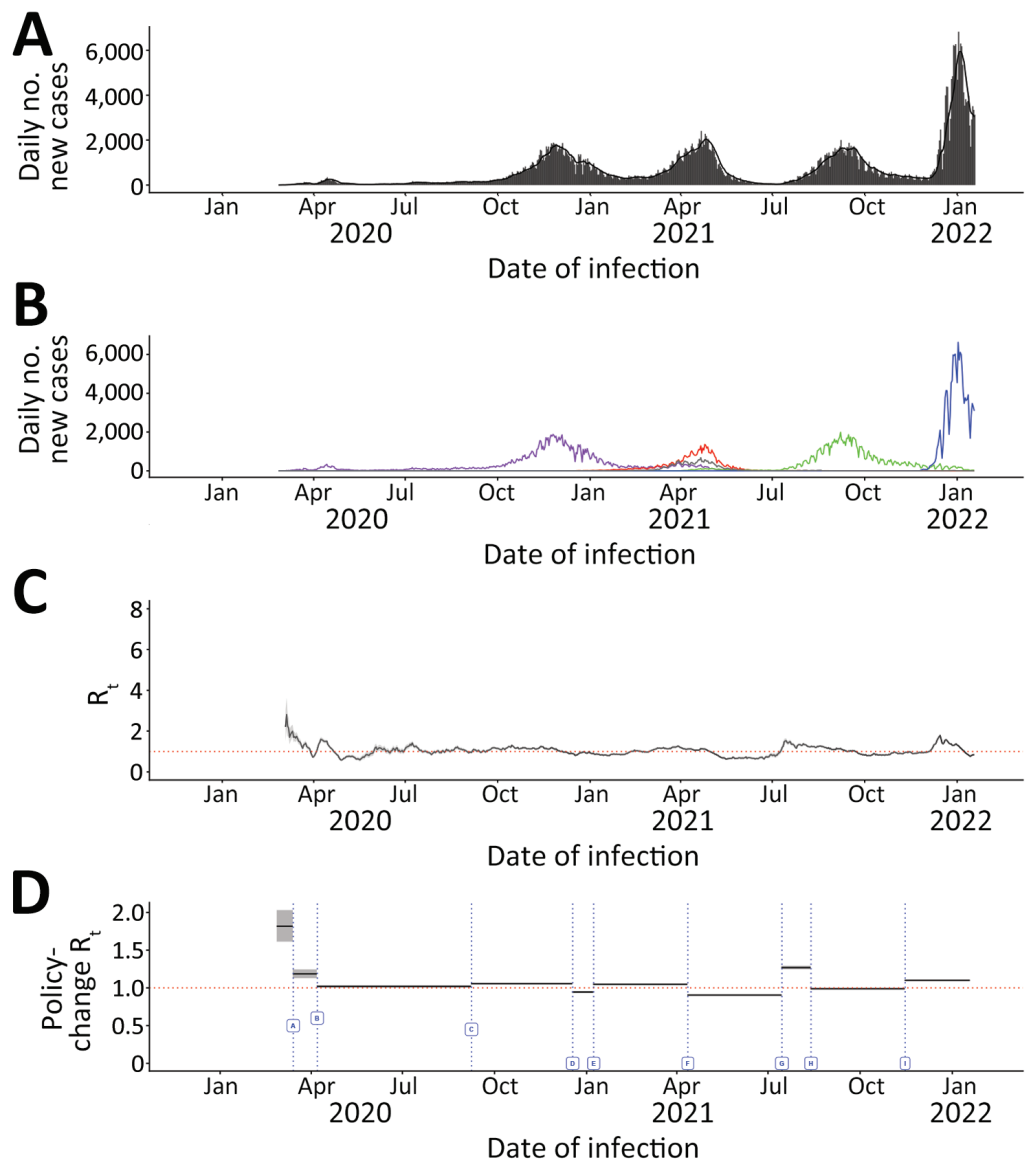
Daily incident COVID-19 case count and 7-day moving average R_t_, by date of infection and policy-change R_t_ or new variants by initiation dates, Alberta, Canada, January 23, 2020–January 27, 2022. Dates are assumed infection dates (i.e., report date minus 9 days); red dotted lines in panels C and D indicate R_t_ = 1. A, B) Incident case count by date of infection for all variants combined (A) and by variant type (B). Colors indicate COVID-19 variants: purple, wild-type; red, Alpha; green, Delta; blue, Omicron; and gray, other variants. C) Seven-day moving average R_t_ by date of infection. D) Policy-change Rt. Policy changes or detection of new variants by dates of initiation: A, school closure (March 14, 2020); B, recommendation for use of face masks (April 7, 2020); C, phased school reopening (September 8, 2020); D, priority populations vaccination rollout (December 18, 2020); E, partial school reopening (January 8, 2021); F, school closure (April 12, 2021); G, increase in cases because of Delta COVID-19 variant (July 15, 2021); H, requirement of vaccination for federal workers (August 13, 2021); I, increase in cases because of the Omicron COVID-19 variant (November 15, 2021).

*Calculations performed by using the instantaneous reproduction number method as implemented in the R package EpiEstim (The R Foundation for Statistical Computing, https://www.r-project.org). The analysis used a γ-distributed serial interval (mean 4.60 days; SD 5.55 days; α = 0.05). R_t_, time-varying reproduction number. †The labels are defined in the text and in the captions of Figures 1–3.

#### British Columbia 

British Columbia experienced 4 major pandemic waves during our study period with greater case burden in metropolitan areas. The first surge, peaking in November 2020, was attributed to wild-type virus; the second, peaking in April 2021, mainly to Alpha; the third, peaking in September 2021, to Delta; and the fourth, peaking in December 2021, to Omicron (Figure 2; Appendix Figures 4, 5). The case counts during the Omicron surge in early December 2021 were substantially higher than during preceding months. During July–November 2020, July–August 2021, and December 2021, British Columbia experienced elevated R_t_ values of 1–2 (Appendix Figure 6).

As in Ontario, the median policy change R_t_ in British Columbia fluctuated as policies were implemented or rescinded and with emergence of new variants (Figure 2; Table 1). From the early cases to the day before school closure in March 2020, a median R_t_ of 1.64 (95% CrI 1.51–1.78) similar to R_t_ in Ontario was observed. In British Columbia, initiating school closures in March 2020 and April 2021, rollout of vaccination for priority populations and required vaccination for government workers were policies associated with decreased median R_t_. In contrast, phased school reopening was associated with increased R_t_. In contrast to Ontario and Alberta, in British Columbia initiating recommendation of face coverings was associated with slightly increased R_t_, and partial school reopening after the holiday break did not substantially affect R_t_. Delta and Omicron emergence led to increased R_t_. As for Ontario, British Columbia also experienced the greatest increase in R_t_, 43.85% (95% CrI 40.71%–46.84%) after Delta emerged. The greatest decrease in R_t_, −42.56% (95% CrI −48.21% to −35.90%), was observed after school closures in March 2020. The lowest median R_t_, 0.86 (95% CrI 0.85–0.87) was achieved after school closures in April 2021. 

#### Alberta 

Similar to British Columbia, Alberta experienced 4 major pandemic waves with greater case burden in metropolitan areas. The first surge, peaking in November 2020, was attributed to wild-type virus; the second, peaking in April 2021, mainly to Alpha; the third, peaking in September 2021, to Delta; and the fourth, peaking in January 2022, to Omicron (Figure 3; Appendix Figures 7, 8). As in other provinces, Omicron led to the highest R_t_ in Alberta during the study period. During September–December 2020, March–May 2021, July–September 2021, and January 2022, Alberta experienced rising transmission rates peaking with R_t_ values of 1–2 (Appendix Figure 9). 

Fluctuations in median policy change R_t_ in Alberta followed a trend similar to those observed in Ontario and British Columbia (Figure 3; Table 1). In Alberta, at the beginning of the study period, median R_t_ was 1.82 (95% CrI 1.61–2.03), higher than the initial R_t_ in Ontario and British Columbia. Initiating school closures in March 2020 and April 2021, recommendation of face coverings, rollout of vaccination for priority populations, and required vaccination for government workers were policies associated with decreased median R_t_; policies that relaxed those interventions, such as phased school reopening and partial school reopening after holiday break were associated with increased median R_t_. Emergence of Delta and Omicron variants also increased R_t_. As in other provinces, the greatest increase in R_t_, 40.27% (95% CrI 37.56%–42.92%), was observed after emergence of Delta, whereas the most significant decrease in R_t_, −34.71% (95% CrI −42.23% to −25.80%), was observed after school closures in March 2020. Similar to other provinces, the lowest median R_t_, 0.91 (95% CrI 0.90, 0.91), was observed after school closure in April 2021.

### R_t_ by Variant 

**Figure 4:**
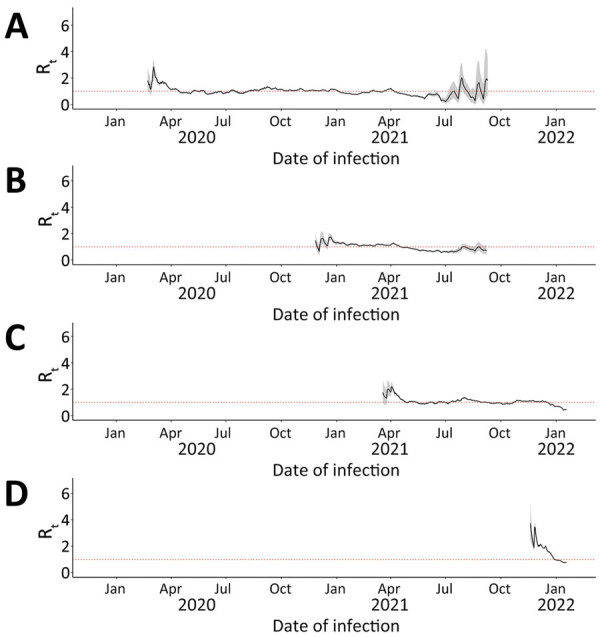
Seven-day sliding-window R_t_ by COVID-19 variant in Ontario, Canada, January 23, 2020–January 27, 2022. A) Wild-type; B) Alpha; C) Delta; D) Omicron. We estimated infection dates by subtracting 9 days from report dates. Red dotted lines indicate R_t_ = 1.

**Figure 5:**
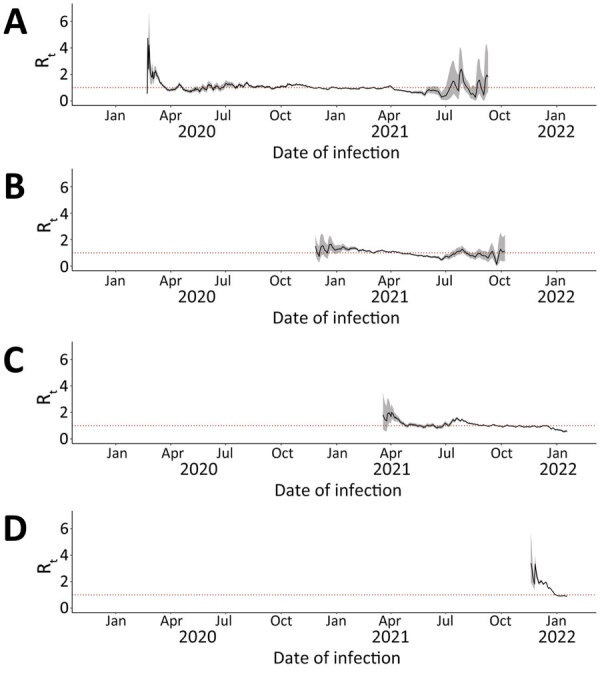
Seven-day sliding-window R_t_ by COVID-19 variant in British Columbia, Canada, January 23, 2020–January 27, 2022. A) Wild-type; B) Alpha; C) Delta; D) Omicron. We estimated infection dates by subtracting 9 days from report dates. Red dotted lines indicate R_t_ = 1.

**Figure 6:**
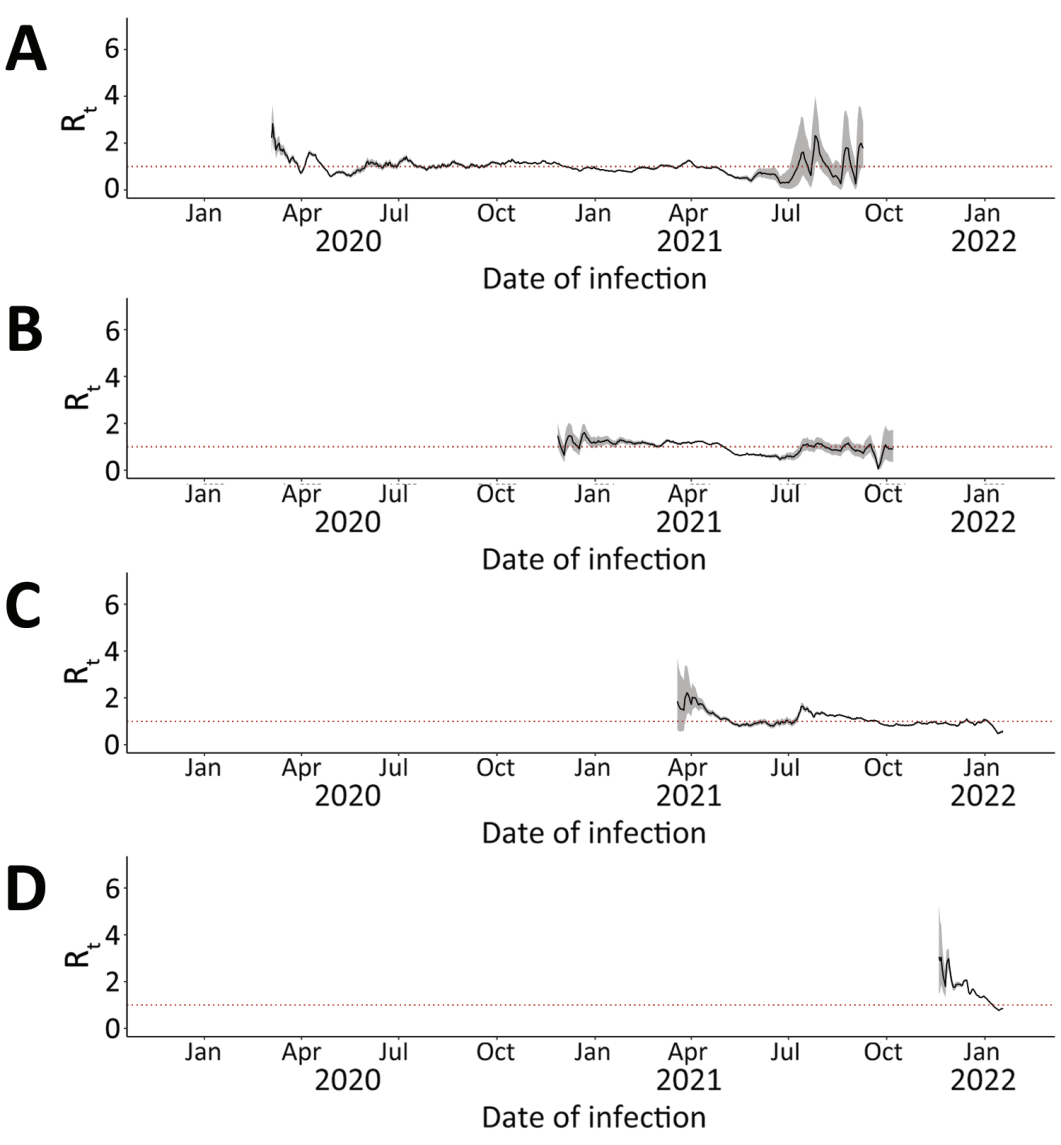
7-day sliding-window R_t_ by COVID-19 variant in Alberta, Canada, January 23, 2020–January 27, 2022. A) Wild-type; B) Alpha; C) Delta; D) Omicron. We estimated infection dates by subtracting 9 days from report dates. Red dotted lines indicate R_t_ = 1.

We calculated median 7-day sliding-window R_t_ by variant for Ontario (Figure 4), British Columbia (Figure 5), and Alberta (Figure 6). We note that each time a new variant emerged in each province, R_t_ was >1: R_t_ was 2–3 for wild-type, 1–2 for Alpha, ≈2 for Delta, and ≈3 for Omicron. Soon after this initial increase, R_t_ would decrease to ≈1. Before wild-type, Alpha, and Delta variants were overtaken by other variants, R_t_ would drop to <1. 

### IRR at the Provincial Level 

We calculated the number of cases per 100,000 person-days for each variant stratified by province and for each province stratified by variant (Table 2). We observed that overall, incidence rate for Omicron was 8.742 (95% CI 8.710–8.774) times that for wild-type, which we used as referent, whereas rates were <0.7 times those for wild-type for Delta (0.692 [95% CI 0.689–0.695]) and Alpha (0.370 [95% CI 0.368–0.372]). When stratified by province, variants followed similar trends, with Omicron having a higher incidence rate and Alpha and Delta lower incidence rates than wild-type, except in British Columbia, where the incidence rate for Delta was higher than for wild-type: IRR 1.126 (95% CI 1.116–1.137). When stratified by variant, with British Columbia as the referent, Alberta had the highest IRR for all variants: wild-type, 1.927 (95% CI 1.912–1.943); Alpha, 1.734 (95% CI 1.711–1.757); Delta, 1.664 (95% CI 1.649–1.678); and Omicron, 1.827 (95% CI 1.801–1.832). Next highest IRRs were for Ontario for all variants except Delta, for which the IRR was 0.519 (95% CI 0.514, 0.524). For all variants combined, with British Columbia as the referent, Alberta (1.799 [95% CI 1.790, 1.807]) had a higher IRR than Ontario (1.117 [95% CI 1.112, 1.121]) (Appendix Tables 8, 9). 

**Table 2:** Number of days observed, cumulative case count, cumulative case count per 100,000 person-days, and incidence rate ratio between variants by province, and between provinces by variant in 3 provinces of Canada (Ontario, British Columbia, and Alberta) during January 23, 2020–January 27, 2022*

Category	Alberta	British Columbia	Ontario	Overall
Population size, 2021	4,262,635	5,000,879	14,223,942	23,487,456
No. days observed				
Variant				
Wild-type	562	573	572	Not applicable
Alpha	602	602	602	Not applicable
Delta	460	460	460	Not applicable
Omicron	68	68	68	Not applicable
Cumulative case count				
Variant				
Wild-type	146,910	91,170	337,299	575,379
Alpha	54,621	36,951	133,162	224,734
Delta	116,902	82,441	121,710	321,053
Omicron	141,661	91,491	366,495	599,646
Total (%)	460,094 (26.74)	302,053 (17.55)	958,666 (55.71)	1,720,812 (100)
Cumulative case count per 100,000 person-days				
Variant				
Wild-type	6.13	3.18	4.15	4.29
Alpha	2.13	1.23	1.56	1.59
Delta	5.96	3.58	1.86	2.97
Omicron	48.87	26.90	37.89	37.54
Total	6.38	3.45	3.96	4.31
IRR between variant by province				
Variant				
Wild-type (95% CI)	Referent	Referent	Referent	Referent
Alpha (95% CI)	0.347 (0.344–0.351)†	0.386 (0.381–0.390)†	0.375 (0.373–0.378)†	0.370 (0.368–0.372)†
Delta (95% CI)	0.972 (0.965–0.980)†	1.126 (1.116–1.137)†	0.449 (0.446–0.452)†	0.692 (0.689–0.695)†
Omicron (95% CI)	7.969 (7.911–8.028)†	8.456 (8.379–8.534)†	9.140 (9.097–9.183)†	8.742 (8.710–8.774)†
IRR between province by variant				
Variant				
Wild-type (95% CI)	1.927 (1.912–1.943)†	Referent	1.303 (1.294–1.313)†	Not applicable
Alpha (95% CI)	1.734 (1.711–1.757)†	Referent	1.267 (1.252–1.282)†	Not applicable
Delta (95% CI)	1.664 (1.649–1.678)†	Referent	0.519 (0.514–0.524)†	Not applicable
Omicron (95% CI)	1.827 (1.801–1.832)†	Referent	1.408 (1.398–1.419)†	Not applicable
Overall (95% CI)	1.799 (1.790–1.807)†	Referent	1.117 (1.112–1.121)†	Not applicable

*IRR, incidence rate ratio. †p<0.0001.

## Discussion 

We found that daily incident case count and R_t_ fluctuations during the study period followed trends consistent across the 3 provinces (Figures 1–3). COVID-19 case count was highest in Ontario, followed by Alberta and British Columbia (Table 2). All 3 provinces experienced surges in case counts and increased R_t_ after Delta and Omicron variants emerged. Nationwide interventions included school closures, face mask recommendations, and rollout of vaccination for priority populations. Our results showed that these interventions were associated with lowered R_t_, which in turn decreased COVID-19 burden. Across the 3 provinces, the school closure policy in April 2021 (policy F) corresponded with the lowest R_t_ during the study period; that decrease underscores the fact that social distancing, in addition to other interventions, can play a substantial role in mitigating spread. However, school closure alone was insufficient to mitigate transmission (*29*,*30*). Our results were consistent with previous studies that suggested mandating face masks (*4*,*31*), physical distancing (*5*), shelters in place (*32*), and school closures (*33*) have been powerful tools for controlling the pandemic. Our study confirms the critical role of high compliance by affected populations towards nonpharmaceutical interventions and other recommended measures for pandemic control before vaccines became available (*34*). Even after vaccine availability, emergence of new variants led to surges with R_t_ >1. Exhausting the numbers of susceptible persons during case surges led to declines in R_t_. 

Policy effectiveness was influenced by variant prevalence at any given time. In agreement with previous studies (*35*–*37*), our study showed that most cases in July–December 2021 were attributed to the Delta variant and in December 2021–January 2022 to the Omicron variant. R_t_ increased in all 3 provinces after emergence of Delta (label G). However, despite having the highest Delta case count, for persons in Ontario, risk of contracting Delta was least across the 3 provinces (Table 2). Also, in contrast with the other 2 provinces, emergence of Omicron did not substantially affect the policy change R_t_ in Ontario. Although Ontario, with its larger population, accounted for more than half of all cases across the 3 provinces, Alberta had the highest total IRR, indicating that residents had the highest risk for COVID-19 among the 3 provinces despite enacting similar mitigation policies. Attitudes and behaviors toward COVID-19 preventive measures could explain the higher total IRR in Alberta. For example, according to 1 study, during the COVID-19 pandemic, Alberta had the highest percentage of antivaccination posts (i.e., tweets) in Canada on the social media site Twitter (now X; https://twitter.com) (*38*). 

After school closures in March 2020, British Columbia was the only one of the 3 provinces that experienced a drastic decrease in the policy change R_t_, from 1.64 to 0.95 (Table 1). After the World Health Organization pandemic declaration on March 11, 2020, British Columbia was the first province to implement stringent public health responses and declare an outbreak (*39*). Also, among the 3 provinces, the highest Oxford Stringency Index score, a measure of adoption of government-recommended policies, was observed in British Columbia both before and after the outbreak declaration (*39*). 

The high incidence rate for Omicron compared with wild-type could be attributed to the high contagiousness of Omicron and its ability to evade naturally acquired immunity against previous strains (*40*,*41*). The lower-than-expected IRR for Alpha might be attributable to the long duration when the variant was prevalent but not the dominant strain; higher person-time under observation for Alpha could have accounted for the lower incidence rate. 

Among limitations in our study, the original data lacked information on date of infection; to account for that missing information, following a simple acceptable method (*42*), we estimated the approximate date of infection by subtracting 9 days (sum of the median delay in reporting and the mean incubation period) from the report date. Second, we assumed the same case-detection rate across provinces. However, if this assumption did not hold, our case burden findings would be skewed. Third, for policy change R_t_, we used dates of specific major policy changes as cutoff points for each time period. In addition, because >1 nonpharmaceutical intervention could be in effect during the same period, we could not attribute changes in R_t_ solely to a single policy change. Fourth, we could not adjust our results to account for differences in levels of policy compliance because those data were not accessible to us. Fifth, potential variation in the availability of COVID-19 testing across the study period and among provinces could affect the daily incident case count reported and thus affect R_t_ estimates. Sixth, we a priori defined an SI distribution for estimating R_t_. Therefore, we could not assess changes in SI because we lacked information on infector-infectee pairs. SI for SARS-CoV-2 could have been shortened over time by use of nonpharmaceutical interventions (*43*). We also did not assess the possibility of variant-specific SI distribution. Seventh, case count data reflected only the subset of SARS-CoV-2 infections testing positive, because many asymptomatic or mildly symptomatic infected persons were never tested. We attempted to demonstrate the effects of that uncertainty in our 7-day sliding-window R_t_ estimates through sensitivity analysis (Appendix Figures 3, 6, and 9). Eighth, lacking population data by public health district, we could not calculate incidence rates by district, which limited the scope of our analysis. Ninth, calculations of provincial variant incidence rates might have been influenced by the subjectively chosen length of duration for variant surges, which we based on the number of days from the index to the last reported cases for each variant in each province. Tenth, ecologic fallacy is possible in a study such as ours using aggregated data; association does not demonstrate causality. 

In conclusion, we observed substantial fluctuations in R_t_ for COVID-19 across 3 provinces in Canada during January 2020–January 2022. Our findings showed that initiating pandemic policies, such as recommendations for face coverings, school closures, and rollout of vaccination for priority populations, were associated with decreases in R_t_. Conversely, relaxing mask mandates and reopening schools were associated with increases in R_t_, especially in conjunction with emergence of new SARS-CoV-2 variants. As mutated variants continue to emerge, public health authorities must remain vigilant to adapt mitigation, testing, and treatment strategies. 

Appendix Additional information from study of SARS-CoV-2 transmission in Alberta, British Columbia, and Ontario, Canada during January 2020–January 2022.
