# Supplementary material for: SARS-CoV-2 Transmission in Alberta, British Columbia, and Ontario, Canada, January 2020–January 2022
Source: Emerg Infect Dis. 2024 May;30(5):956–67. doi: 10.3201/eid3005.230482 (PMC11060455; doi:10.3201/eid3005.230482)
Supplement: Appendix — Additional information from study of SARS-CoV-2 transmission in Alberta, British Columbia, and Ontario, Canada during January 2020–January 2022. [file 23-0482-Techapp-s1.pdf]

Article DOI: https://doi.org/10.3201/eid3005.230482

EID cannot ensure accessibility for Appendix materials supplied by authors. Readers who have difficulty accessing Appendix content should contact the authors for assistance.

SARS-CoV-2 Transmission in Alberta, British Columbia, and Ontario, Canada, January 2020–January 2022

Appendix

Background

The first imported COVID-19 case in Canada was reported in Toronto on January 23, 2020 (*1*,*2*). On March 17, 2020, Alberta, British Columbia, and Ontario declared public health emergencies due to the rapid spread of COVID-19 (3–5). Before the availability of a vaccine, Canada imposed social distancing measures that were generally more stringent than those in the United States. As vaccines became available, life in Canada started to return to a 'new normal' where some of these measures were gradually lifted, and residents could resume previously restricted activities. As of March 6, 2023, Canada had reported a total of 4,600,615 cases and 51,447 deaths attributed to SARS-CoV-2 (*6*). The emergence of highly transmissible variants, such as Delta and Omicron, coincided with surges in cases in Canada (*7*,*8*) with the Delta variant (*8*,*9*) and the Omicron variant (*8*,*10*) being first detected in April and November 2021 respectively.

Characteristics of Study Population

The population data for our study was obtained from the Government of Ontario (*11*), the British Columbia Center for Disease Control (*12*), and the Government of Alberta (*13*). A breakdown of the total and provincial age and sex makeup of our study populations is provided (Appendix Tables 1–3).

Policy and COVID-19 Variant Emergence

Using information obtained from the Government of Canada (*14*), the Canadian Institute for Health Information (*15*), and school year calendars from the Upper Canada District School

Board (*16*), notable COVID-19 mitigation policies and variant-specific emergence were identified for each province (Appendix Table 4). These dates were used to create the policy change R_t figures for Ontario, British Columbia, and Alberta (Figures 1–3).

Methods

Sex/Gender in the dataset

It is critical to note that in the line list datasets of COVID-19 cases, Alberta specified 'gender' (female, male, unknown, and NA), British Columbia specified 'sex' (female, male, and unknown) and Ontario specified 'gender' (female, male and other). For consistency in this study, we standardized and used 'sex' for all instances and all provinces.

R Package Versions

'EpiEstim' package version 2.2.4 was used to estimate R_t; 'sf' package version 1.0.9 was used to create maps. The 95% confidence intervals for IRRs were calculated using the 'fmsb' package version 0.7.5.

R_t estimation

The 7-day sliding-window R_t estimates were calculated using the average of R_t over a 7-day sliding-window. "Policy change R_t" was calculated using the average daily R_t estimates over a non-overlapping time-window between two timepoints. The percentage change for the non-overlapping window R_t and its 95% credible interval (CrI) for all three provinces were calculated by bootstrapping. The policy change R_t estimate at each policy interval was compared with the previous policy interval, i.e., $(t_2 - t_1) / t_1$. At each policy interval, 1,000 random numbers were drawn from each distribution of policy change R_t estimate to obtain a distribution of percentage change in R_t over a policy change and thus calculate the 95% CrI.

Variant R_t

The variant case counts were generated by multiplying the daily provincial case count by the weekly COVID-19 variant percentage using Microsoft Excel (Redmond, WA). Each variant was plotted over the longest time period with confirmed cases; cases that were separated by 14 or more days of no cases were excluded from the plotted time period. The estimated variant cases were used to generate an epidemic curve showing the incidence of each variant. Variant R_t was estimated using a 7-day sliding-window.

Sensitivity analyses of R_t using estimated infection count

Two sensitivity analyses were conducted to estimate R_t using estimated infection counts. Two multipliers were used based on two different sources for the infection-to-case ratio of COVID-19. We assumed that on average 1 in 4 (*17*), and 1 in 11 (*18*), infected persons, respectively, were reported as cases. For each day of the case count time series, a multiplier was drawn from a Poisson distribution with a mean of 4. This generated a hypothetical time series of daily numbers of new infections. The process was repeated 1,000 times to generate 1,000 hypothetical time series, from which R_t estimates were generated. For each day we sampled 10 random values from the posterior distribution of each of the 1,000 R_t estimates (in total 10,000 values) to generate the 95% CrI of the R_t. The process is repeated for multipliers drawn from a Poisson distribution with a mean of 11.

Incidence rate and incidence rate ratio (IRR)

Regarding IRR between provinces, British Columbia served as the reference group, due to it having the lowest case count per person-days among the 3 provinces, for the calculation of the IRR for Alberta and Ontario by sex and age group. Due to differences in the start date of case reporting, the actual durations observed were different for each province: Alberta – 693 days, British Columbia – 730 days, and Ontario – 736 days.

Using SARS-CoV-2 Wild-type as the reference, the IRR of Alpha, Delta, and Omicron variants were calculated by province. Incidence rate and IRR of variants for all three provinces were calculated with variant-specific durations of observation: Alberta: Wild-type – 562 days, Alpha – 602 days, Delta – 460, and Omicron – 68 days; British Columbia: Wild-type – 573 days, Alpha – 602 days, Delta – 460 days, Omicron – 68 days; and Ontario: Wild-type – 572, Alpha – 602 days, Delta – 460 days, and Omicron – 68 days.

Results

Ontario

Weekly incidence curves for age, sex, and subprovincial public health regional area (PHRA) (*19*) are displayed in Appendix Figure 1. In panel 1 we found that all age groups followed a similar epidemiologic wave pattern with the highest incidence in the under 20 age group at every peak. Panel 2 shows the weekly incidence by sex. No major differences in

incidence were observed over time between the two sexes. Lastly, panel 3 clearly shows that across our study period, the Central PHRA had the highest sustained weekly incidence across all the other PHRAs.

It is of note that although cumulative case count in the under 20 age group may appear to be high and there were more female cases than male cases (Appendix Figure 1, Appendix Table 5), 20–29-year-old females had the highest cumulative case count of 12,468 per 100,000 persons followed by males 80 years or older (11,540 per 100,000 persons). Appendix Table 5 displays the cumulative case counts within Ontario by sex and age. Generally, females had a higher total burden than males (cumulative case count of 7,579 per 100,000) and persons 20–29 years-old had the greatest total burden (cumulative case count of 11,845.9 per 100,000) than any other age group. The total disease burden of COVID-19 observed in Ontario was 7,451 per 100,000 persons.

During the early stages of the pandemic, the south-eastern subprovincial areas with large metropolitan areas like Toronto, had an average monthly cumulative case count at least 10-fold greater than that of the other regions (Appendix Figure 2). By the end of 2021 and January 2022, the North-East and North-West PHUs had a monthly cumulative case count in the 10,000s while the metropolitan PHUs', Central, East, Toronto, and West, numbers were in the 100,000s.

In Ontario, the 7-day sliding-window median R_t results showed (Appendix Figure 3: first panel) that for most parts of the time series, the 95% CrI of R_t estimates spanned across 1. The exceptions were at the beginning of the pandemic and in December 2021 when $R_t > 1$ with its 95% CrI lower bound >1. The sensitivity analysis using the estimated daily number of new infections (mean multiplier, $\lambda = 4$ and $\lambda = 11$) showed that all R_t graphs followed a similar trend but with a different 95% CrI (Appendix Figure 3: second and third panels). The widest CrI band was observed for R_t estimates obtained using the estimated number of infections assuming one reported case in four infections, followed by one reported case in eleven infections, and the narrowest CrI was observed for R_t estimates using case count as inputs.

British Columbia

Weekly incidence curves for age, sex, and subprovincial regional health authorities (RHA) (*20*) are displayed in Appendix Figure 4. In panel 1 we found that all age groups followed a similar epidemiologic wave pattern with the highest incidence in the under 20 age group at

every peak. Panel 2 shows that the weekly incidence by sex. No major differences in incidence were observed over time between the two sexes. Lastly, panel 3 clearly shows that across our study period, the Fraser RHA had the highest sustained weekly incidence across all the other RHAs.

It is of note that although the cumulative case count in the under 20 age group may appear to be high and there were more female cases than male cases in general (Appendix Figure 4), 20–29-year-old females had the highest cumulative case count of 10,930 per 100,000 persons followed by males in the same age group at 10,023 per 100,000 persons. Appendix Table 6 displays the cumulative case counts within British Columbia based on sex and age. Generally, males had a higher total burden (cumulative case count of 6,402 per 100,000) than females, and persons between 20–29-years-old had the greatest total burden (cumulative case count of 10,515 per 100,000) than any other age group. The total disease burden of COVID-19 seen in British Columbia was 6,412 cases per 100,000 persons.

During the early stages of the pandemic, the Fraser RHA had a monthly cumulative case count in the 1,000s while the other regions' counts were in their 100s (Appendix Figure 5), even though it does not contain the most populous cities. Surprisingly this trend remained and by the end of 2021 and January 2022, the Fraser RHA had a monthly cumulative case count in the 100,000s while other RHAs were in the 10,000s.

The 7-day sliding-window median R_t showed in British Columbia (Appendix Figure 6: first panel) that for most parts of the time series, the 95% CrI of R_t estimates spanned across 1. The exceptions were in June 2021 when R_t <1 and surges of R_t at the beginning of the pandemic, and in July 2021 and December 2021 when R_t >1 with its lower bound >1. The sensitivity analysis using the estimated daily number of new infections (mean multiplier, $\lambda = 4$ and $\lambda = 11$) has been shown in the second and third panels in Appendix Figure 6. The R_t values are similar in all R_t graphs with a different 95% CrI. Similar to Ontario, the widest CrI was observed for R_t estimates obtained using the estimated number of infections assuming one reported case in four infections, followed by one in eleven, and the narrowest CrI was observed for R_t estimates using case count as inputs.

Alberta

Weekly incidence curves for age, sex, and subprovincial health services zones (HSZ) (*21*) are displayed in Appendix Figure 7. In panel 1 we found that all age groups followed a similar epidemiologic wave pattern with the highest incidence in the under 20 age group at every peak. Panel 2 shows that the weekly incidence by sex. No major differences in incidence were observed over time between the two sexes. Lastly, panel 3 clearly shows that across our study period, the Calgary HSZ had the highest sustained weekly incidence across all the other HSZs.

It is of note that although the cumulative case count may appear to be high in the <20 age group and there were more female cases than male cases in general (Appendix Figure 7), 20–29-year-old females had the highest cumulative case count of 17,889 per 100,000 persons followed by males in the same age group at 15,801 per 100,000 persons. Appendix Table 7 displays the cumulative case counts within Alberta based on sex and age. Generally, females had a higher total burden (cumulative case count of 11,659 per 100,000) than males, and persons between the age of 20–29 had the greatest total burden (cumulative case count of 16,847 per 100,000) than any other age group. The total disease burden of COVID-19 seen in Alberta was 11,428 cases per 100,000 persons.

During the early stages of the pandemic, Calgary and South subprovincial HSZs had a monthly cumulative case count in the 1,000s while the other regions were in their 100s (Appendix Figure 8). In contrast, by the end of 2021 and January 2022, the populous Calgary and Edmonton HSZs had a monthly cumulative case count in the 100,000s while other regions were in the 10,000s.

In Alberta, the 7-day sliding-window median R_t showed that for most parts of the time series, the 95% CrI of R_t estimates spanned across 1 (Appendix Figure 9: first panel). The exceptions include the beginning of the pandemic, May-June 2021 when R_t <1, and peaks of R_t in July 2021 and December 2021 when R_t >1 with its 95% CrI lower bound >1. Appendix Figure 9 showed a similar trend in all R_t graphs but with a different 95% CrI. Similar to other provinces, the widest CrI was observed for R_t estimates obtained using the estimated number of infections assuming one reported case in four infections, followed by one in eleven, and the narrowest CrI was observed for R_t estimates using case count as inputs.

IRR

Appendix Table 8 presents the 2021 population and cumulative case counts of each variant by province. Despite having the smallest population, Alberta had the largest cumulative case count per 100,000 overall of all three provinces (11,435.1 per 100,000). The Omicron variant had the highest cumulative case count (2,553.1 per 100,000) just higher than the Wild-type (2,449.7 per 100,000).

Cumulative case count per person-day was used to calculate the IRR for each age group stratified by sex, with British Columbia used as the reference (Appendix Table 9). Among females, the largest IRR observed was among those who were 80 or older residing in Alberta (IRR = 2.274, 95% CI, 2.198, 2.352); among males, the largest was seen in those 80 or older in Ontario (IRR = 2.671, 95% CI, 2.584, 2.791); and overall, the largest IRR was found in the Alberta population 80 years or older (IRR = 2.195, 95% CI, 2.139, 2.253). In general, for almost all of the categories stratified by sex and age, Alberta had a higher incidence rate than that of the other two provinces in the same categories.

Discussion

Females in the 20–29 age group in all provinces are likely to have been more socially mobile leading to a larger disease burden. Alternatively, it could be that they were more likely to seek testing when feeling ill than other groups in all provinces. The overall cumulative case count of 11,427.9 per 100,000 in Alberta was 53.4% more than that of Ontario and 78.2% more than that of British Columbia. However, when stratified by age and sex, the IRR between provinces showed that males in Ontario who were 80 years or older had a higher disease burden with an IRR of 2.8 when compared with British Columbia. Females in the same age range in Alberta had an IRR of 2.3 compared with British Columbia making them the second most burdened group by age and sex.

When comparing by variant, Omicron and the Wild-type accounted for 64.1% of all cumulative cases. Although these two variants appear to have a similar disease burden, this can be attributed to our study period cutoff; across provinces, the Wild-type variant was prevalent for an average of 569 days whereas Omicron was prevalent for an average of 68 days within our study period. Therefore, in Table 2 we calculated the variant IRR using cumulative case count

per 100,000 person days. Using the Wild-type as the reference group and stratified by province, Omicron had the highest IRR of 9.1 in Ontario, followed by 8.5 in British Columbia, and 8.0 in Alberta. Regarding the overall totals in Table 2 in the main text, Alberta had the greatest burden of COVID-19 with an IRR of 1.8, followed by Ontario (IRR = 1.1), when compared with British Columbia. Stratified by age and sex and also by variant, Alberta was found to have a higher burden of cases than British Columbia and Ontario.

References

1. Silverstein WK, Stroud L, Cleghorn GE, Leis JA. First imported case of 2019 novel coronavirus in Canada, presenting as mild pneumonia. Lancet. 2020;395:734. PubMed https://doi.org/10.1016/S0140-6736(20)30370-6

2. Schwartz KL, Murti M, Finkelstein M, Leis JA, Fitzgerald-Husek A, Bourns L, et al. Lack of COVID-19 transmission on an international flight. CMAJ. 2020;192:E410. PubMed https://doi.org/10.1503/cmaj.75015

3. Ontario Office of the Premier. Ontario enacts declaration of emergency to protect the public [cited 2022 Jun 03]. https://news.ontario.ca/en/release/56356/ontario-enacts-declaration-of-emergency-to-protect-the-public

4. Bartko K, Heidenreich P. Premier Jason Kenney declares COVID-19 public health emergency in Alberta [cited 2022 Jun 03]. https://globalnews.ca/news/6690858/alberta-coronavirus-march-17-2020/

5. BC Gov News. Province declares state of emergency to support COVID-19 response [cited 2022 Jun 03]. https://news.gov.bc.ca/releases/2020PSSG0017-000511

6. Government of Canada. COVID-19 epidemiology update: key updates [cited 2023 Mar 12]. https://health-infobase.canada.ca/covid-19/

7. Li T, Maier A, Carter M, Guan TH. Omicron and S-gene target failure cases in the highest COVID-19 case rate region in Canada—December 2021. J Med Virol. 2022;94:1784–6. PubMed https://doi.org/10.1002/jmv.27562

8. Otto SP, Day T, Arino J, Colijn C, Dushoff J, Li M, et al. The origins and potential future of SARS-CoV-2 variants of concern in the evolving COVID-19 pandemic. Curr Biol. 2021;31:R918–29. PubMed https://doi.org/10.1016/j.cub.2021.06.049

9. Fisman DN, Tuite AR. Evaluation of the relative virulence of novel SARS-CoV-2 variants: a retrospective cohort study in Ontario, Canada. CMAJ. 2021;193:E1619–25. PubMed https://doi.org/10.1503/cmaj.211248

10. Lord C. 1st Omicron cases in Canada landed at Montreal’s Trudeau International Airport [cited 2022 Jun 04]. https://globalnews.ca/news/8409802/ottawa-omicron-covid-montreal-airport/

11. Government of Ontario. Confirmed positive cases of COVID-19 in Ontario [cited 2022 January 28]. https://open.canada.ca/data/en/dataset/f4112442-bdc8-45d2-be3c-12efae72fb27

12. British Columbia Centre for Disease Control. Archived BC COVID-19 data [cited 2022 January 28]. http://www.bccdc.ca/health-info/diseases-conditions/covid-19/archived-b-c-covid-19-data

13. Government of Alberta. COVID-19 Alberta statistics [cited 2022 January 28]. https://web.archive.org/web/20220128231728/https://www.alberta.ca/stats/covid-19-alberta-statistics.htm

14. Government of Canada, Department of Justice. Government of Canada's response to COVID-19 [cited 2022 May 02]. https://www.justice.gc.ca/eng/csj-sjc/covid.html#shr-pg0

15. Canadian Institute for Health Information. COVID-19 intervention timeline in Canada [cited 2022 May 02]. https://www.cihi.ca/en/covid-19-intervention-timeline-in-canada

16. Upper Canada District School Board. 2021–2022 school year calendar [cited 2022 May 18]. https://web.archive.org/web/20220518125851/https://www.ucdsb.on.ca/for_families/school_year_calendar

17. Centers for Disease Control and Prevention. Estimated COVID-19 burden [cited 2022 August 28]. https://stacks.cdc.gov/view/cdc/117147

18. Centers for Disease Control and Prevention. COVID-19 Pandemic planning scenarios [cited 2022 August 28]. https://www.cdc.gov/coronavirus/2019-ncov/hcp/planning-scenarios.html

19. Public Health Ontario. Health services locator map [cited 2022 Sep 13]. https://www.publichealthontario.ca/en/data-and-analysis/commonly-used-products/maps/health-services-locator

20. Government of British Columbia. Regional health authorities [cited Sep 10, 2022]. https://www2.gov.bc.ca/gov/content/health/about-bc-s-health-care-system/partners/health-authorities/regional-health-authorities

21. Alberta Health Services. AHS map and zone overview [cited 2022 Sep 10]. https://www.albertahealthservices.ca/assets/about/publications/ahs-ar-2021/zones.html

Appendix Table 1. Characteristics of the study population in Ontario.

Age group, y	F	M	Other sex	Total (3 provinces)
<20	88,002	91,794	932	180,728
20–29	113,248	106,911	1,478	221,637
30–39	91,373	83,615	902	175,890
40–49	80,597	68,257	720	149,574
50–59	71,795	65,403	537	137,735
60–69	40,380	42,221	343	82,944
70–79	20,378	20,880	160	41,418
≥80	26,645	16,257	166	43,068
Unknown	113	121	66	300
Total	532,531	495,459	5,304	1,033,294

Appendix Table 2. Characteristics of the study population in British Columbia.

Age group, y	F	M	Other sex	Total (3 provinces)
<20	29,183	31,268	163	60,614
20–29	33,854	32,273	297	66,424
30–39	31,604	29,060	235	60,899
40–49	24,731	22,232	180	47,143
50–59	19,116	18,612	136	37,864
60–69	11,941	12,850	95	24,886
70–79	5,750	6,437	34	12,221
≥80	5,894	4,479	29	5,894
Unknown	31	24	32	87
Total	162,104	157,235	1,201	320,540

Appendix Table 3. Characteristics of the study population in Alberta.

Age group, y	F	M	Other sex	Total (3 provinces)
<20	54,914	58,130	91	113,044
20–29	45,993	42,204	118	88,197
30–39	50,110	45,767	71	95,877
40–49	40,778	37,699	41	78,477
50–59	27,219	26,932	31	54,151
60–69	14,941	15,910	17	30,851
70–79	6,722	6,777	8	13,499
≥80	7,620	5,022	9	12,642
Unknown	159	143	10	302
Total	248,456	238,584	396	487,436

Appendix Table 4. Policies implemented by government agencies and the time of the emergence of COVID-19 variants in Canada (*14–16*).

Label†	Description	Policy effective date/ variant emergence date	Effective until
	Recommendation of work-from-home policies implemented	2020 Mar 10	NA
A	School closure	2020 Mar 14	NA
B	Recommendation for the use of face masks	2020 Apr 7	NA
	Mandatory face masks for transportation workers	2020 Jun 4	NA
C	Phase school reopening	2020 Sep 8	2020 Sep 21
D	Priority populations vaccination rollout	2020 Dec 18	2021 Dec 8
	Online school	2021 Jan 4	NA
E	Partial school reopening	2021 Jan 8	2021 Feb 21
F	School closure	2021 Apr 12	2021 Sep 7
	Approved revised 2nd vaccine dose	2021 Mar 1	NA
	Released guidelines for safety measures	2021 Jun 25	NA
G	Increase in cases from Delta variant (approximate dates)	2021 Jul 1 (Ontario/British Columbia)<br>2021 Jul 15 (Alberta)	NA
H	Requirement of vaccination for federal workers	2021 Aug 13	NA
	Vaccination booster for immunocompromised	2021 Sep 10	NA
I	Increase in cases from Omicron variant (approximate dates)	2021 Nov 15 (Alberta)<br>2021 Nov 21 (British Columbia)<br>2021 Nov 25 (Ontario)	NA

*NA, Not applicable
†Label corresponds to events indicated in Figures 1, 2, and 3.

Appendix Table 5. Cumulative case count and cumulative case count per 100,000 persons of each SARS-CoV-2 variant by sex and age group in Ontario, Canada as of the date of report of January 27, 2022.

Category	Age group, y									
	<20	20–29	30–39	40–49	50–59	60–69	70–79	≥80	Unknown	Total
	2021 population									
Sex										
F	1,484,130	908,290	978,605	926,300	1,018,310	925,515	391,040	394,095	NA	7,026,285
M	1,569,115	962,710	950,635	858,440	963,120	854,280	542,920	140,872	NA	6,842,092
Total	3,053,245	1,871,000	1,929,240	1,784,740	1,981,430	1,779,795	933,960	534,967	NA	13,868,377
	Cumulative case count									
Sex										
F	88,002	113,248	91,373	80,597	71,795	40,380	20,378	26,645	113	532,531
M	91,794	106,911	83,615	68,257	65,403	42,221	20,880	16,257	121	495,459
Unknown	932	1,478	902	720	537	343	160	166	66	5,304
F, %	48.7	51.1	51.9	53.9	52.1	48.7	49.2	61.9	37.7	51.5
M, %	50.8	48.2	47.5	45.6	47.5	50.9	50.4	37.7	40.3	48.5
Total	180,728	221,637	175,890	149,574	137,735	82,944	41,418	43,068	300	1,033,294
	Cumulative case count /100,000 persons									
Sex										
F	5,929.5	12,468.3	9,337.1	8,701.0	7,050.4	4,363.0	5,211.2	6,761.1	NA	7,579.1
M	5,850.0	11,105.2	8,795.7	7,951.3	6,790.7	4,942.3	3,845.9	11,540.3	NA	7,241.3
Total	5,919.2	11,845.9	9,117.1	8,380.7	6,951.3	4,660.3	4,434.7	8,050.6	NA	7,450.7

NA, not applicable

Appendix Table 6. Cumulative case count and cumulative case count per 100,000 of each SARS-CoV-2 variant by sex and age group in British Columbia, Canada as of the date of report of January 27, 2022.*

Category	Age group, y									
	<20	20–29	30–39	40–49	50–59	60–69	70–79	≥80	Unknown	Total
	2021 population									
Sex										
F	470,095	309,730	357,835	323,775	354,285	351,620	237,010	139,015	NA	2,543,365
M	500,495	322,005	354,100	306,000	331,060	322,915	216,430	104,510	NA	2,457,517
Total	970,590	631,735	711,935	629,775	685,345	674,535	453,440	243,525	NA	5,000,880
	Cumulative case count									
Sex										
F	29,183	33,854	31,604	24,731	19,116	11,941	5,759	5,894	31	162,113
M	31,358	32,273	29,060	22,232	18,612	12,850	6,437	4,479	24	157,325
Unknown	163	297	235	189	136	95	34	29	32	1,291
F, %	48.1	51.0	51.9	52.5	50.5	48.0	47.1	56.7	35.6	50.6
M, %	51.7	48.6	47.7	47.2	49.2	51.6	52.6	43.1	27.6	49.1
Total	60,704	66,424	60,899	47,143	37,864	24,886	12,230	10,402	87	320,639
	Cumulative case count /100,000 persons									
Sex										
F	6,207.9	10,930.2	8,832.0	7,638.3	5,395.7	3,396.0	2,429.9	4,239.8	NA	6,374.0
M	6,265.4	10,022.5	8,206.7	7,265.4	5,621.9	3,979.4	2,974.2	4,285.7	NA	6,401.8
Total	6,254.3	10,514.5	8,554.0	7,485.7	5,524.8	3,689.4	2,697.2	4,271.4	NA	6,411.7

NA, not applicable

Appendix Table 7. Cumulative case count and cumulative case count per 100,000 of each SARS-CoV-2 variant by sex and age group in Alberta, Canada as of the date of report of January 27, 2022.*

	Age group, y									
Category	<20	20–29	30–39	40–49	50–59	60–69	70–79	≥80	Unknown	Total
					2021 population					
Sex										
F	514,785	257,105	333,645	292,020	269,445	243,935	140,395	83,370	NA	2,134,700
M	544,615	267,105	328,565	293,420	267,475	238,575	129,010	59,170	NA	2,127,935
Total	1,059,400	524,210	662,210	585,440	536,920	482,510	26,905	142,540	NA	4,262,635
					Cumulative case count					
Sex										
F	54,914	45,993	50,110	40,778	27,219	14,941	6,722	7,629	159	248,465
M	58,130	42,204	45,767	37,699	26,932	15,910	6,777	5,022	143	238,584
Unknown	86	118	71	41	31	17	8	9	10	381
F, %	48.5	52.1	52.2	51.9	50.2	48.4	49.8	60.3	51.0	51.0
M, %	51.4	47.8	47.7	48.0	49.7	51.5	50.2	39.7	45.8	49.0
Total	113,130	88,315	95,948	78,518	54,182	30,868	13,507	12,660	312	487,128
					Cumulative case count /100,000 persons					
Sex										
F	10,667.4	17,888.8	15,019.0	13,964.1	10,101.9	6,125.0	4,787.9	9,150.8	NA	11,639.3
M	10,673.6	15,800.5	13,929.4	12,848.1	10,069.0	6,668.8	5,253.1	8,487.4	NA	11,212.0
Total	10,678.7	16,847.3	14,489.1	13,411.8	10,091.3	6,397.4	5,013.6	8,881.7	NA	11,427.9

NA, not applicable

Appendix Table 8. Cumulative case count and cumulative case count per 100,000 persons of each SARS-CoV-2 variant stratified by province (Alberta, British Columbia, and Ontario) as of the date of report of January 27, 2022.

Variant	Alberta	British Columbia	Ontario	Total
		2021 population		
	4,262,635	5,000,879	14,223,942	23,487,456
		Cumulative case count		
Wild-type	146,910	91,170	337,299	575,379
Alpha	54,621	36,951	133,162	224,734
Delta	116,902	82,441	121,710	321,053
Omicron	141,661	91,491	366,495	599,647
Other	27,342	18,482	66,556	112,380
Total	487,436	30,535	1,025,222	1,833,193
		Cumulative case count /100,000 persons		
Wild-type	3,446.5	1,823.1	2,371.3	2,449.7
Alpha	1,281.4	738.9	936.2	956.8
Delta	2,742.5	1,648.5	855.7	1,366.9
Omicron	3,323.3	1,829.5	2,576.6	2,553.1
Other	641.4	369.6	467.9	478.5
Total	11,435.1	6,409.6	7,207.7	7,805.0

Appendix Table 9. Cumulative case count per 100,000 person-days and incidence rate ratio between Alberta, British Columbia, and Ontario provinces, Canada by sex and age group.*

	Cumulative case count /100,000 person-days			Incidence rate ratio			
				IRR	95% CI	IRR	95% CI
Age	AB	BC	ON	AB		ON	
			Females				
Age group, y							
<20	15.39	8.50	8.06	1.810	1.785–1.836	0.947	0.935–0.960
20–29	25.81	14.97	16.94	1.724	1.700–1.748	1.131	1.118–1.145
30–39	21.67	12.10	12.69	1.791	1.766–1.817	1.049	1.035–1.062
40–49	20.15	10.46	11.82	1.926	1.896–1.956	1.130	1.114–1.146
50–59	14.58	7.39	9.58	1.972	1.936–2.009	1.296	1.276–1.317
60–69	8.84	4.65	5.93	1.900	1.855–1.946	1.274	1.249–1.301
70–79	6.91	3.33	7.08	2.076	2.004–2.150	2.127	2.066–2.190
≥80	13.20	5.81	9.19	2.274	2.198–2.352	1.582	1.538–1.627
Total	16.78	8.73	10.30	1.923	1.911–1.935	1.179	1.182–1.196
			Males				
Age group, y							
<20	14.62	8.58	7.95	1.704	1.680–1.727	0.926	0.914–0.938
20–29	21.64	13.73	15.09	1.577	1.554–1.600	1.099	1.085–1.113
30–39	19.08	11.24	11.95	1.697	1.673–1.722	1.063	1.049–1.077
40–49	17.60	9.95	10.80	1.768	1.739–1.798	1.085	1.069–1.102
50–59	13.79	7.70	9.23	1.791	1.758–1.825	1.198	1.179–1.218
60–69	9.14	5.45	6.72	1.676	1.637–1.715	1.232	1.208–1.256
70–79	7.20	4.07	5.23	1.766	1.07–1.828	1.283	1.247–1.319
≥80	11.63	5.87	15.68	1.980	1.902–2.062	2.671	2.584–2.761
Total	16.17	8.77	9.84	1.844	1.832–1.856	1.122	1.115–1.128
			All				
Age group, y							
<20	15.40	8.54	8.00	1.802	1.784–1.820	0.936	0.928–0.945
20–29	24.28	14.34	15.99	1.693	1.676–1.710	1.115	1.105–1.125
30–39	20.89	11.67	12.32	1.790	1.772–1.808	1.056	1.046–1.066
40–49	19.34	10.22	11.33	1.894	1.872–1.915	1.109	1.098–1.121
50–59	14.55	7.54	9.41	1.930	1.905–1.955	1.248	1.233–1.262
60–69	9.23	5.03	6.31	1.833	1.802–1.863	1.252	1.235–1.270
70–79	7.23	3.68	6.00	1.962	1.915–2.011	1.629	1.596–1.662
≥80	12.81	5.83	10.90	2.195	2.139–2.253	1.867	1.828–1.908
Total	16.48	8.75	9.82	1.883	1.875–1.892	1.122	1.118–1.127

*AB, Alberta; BC, British Columbia; ON, Ontario IRR, incidence rate ratio
†British Columbia referent group

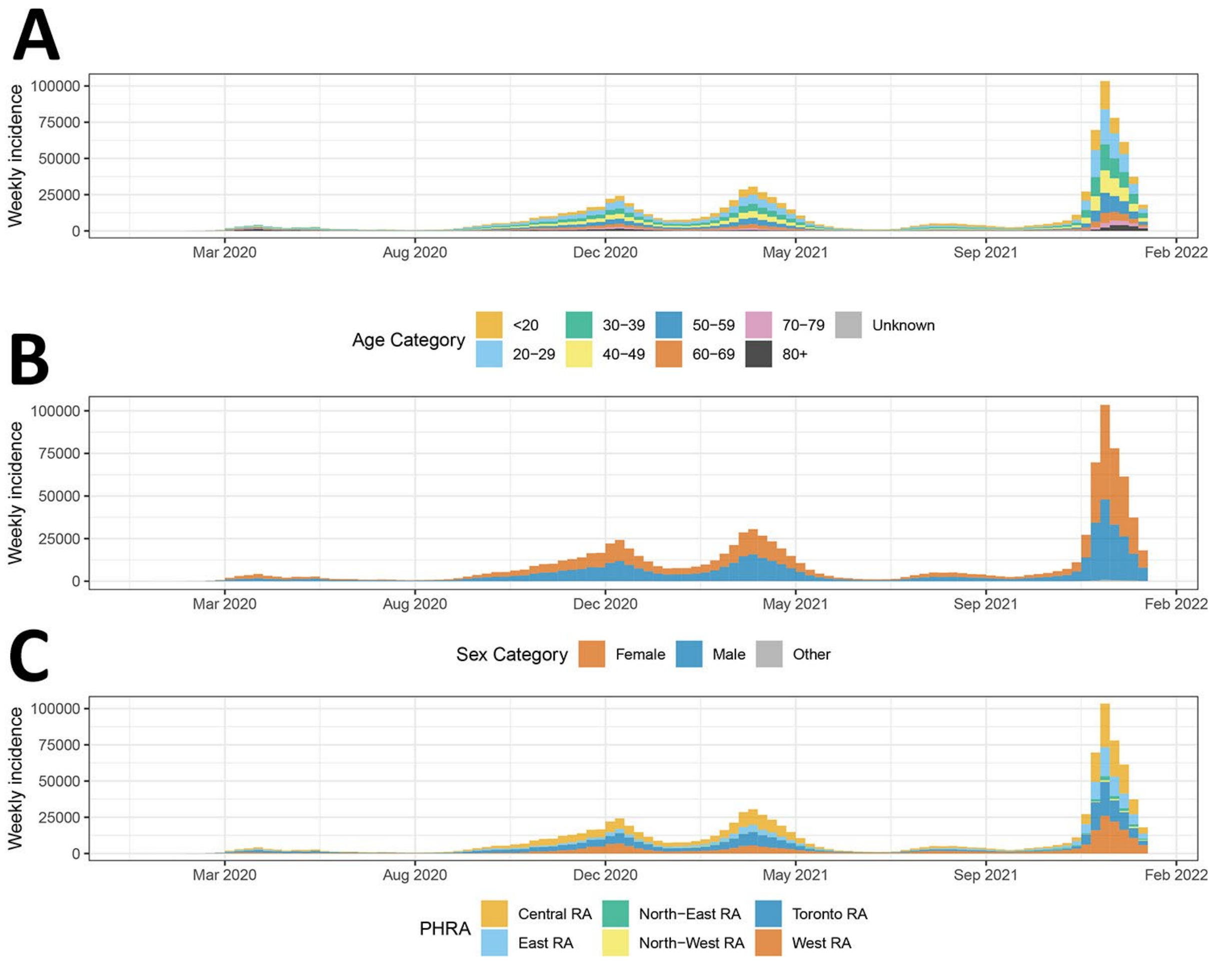

Appendix Figure 1. Descriptive weekly incidence curves by A) age, B) sex, and C) subprovincial public health regional area in Ontario.

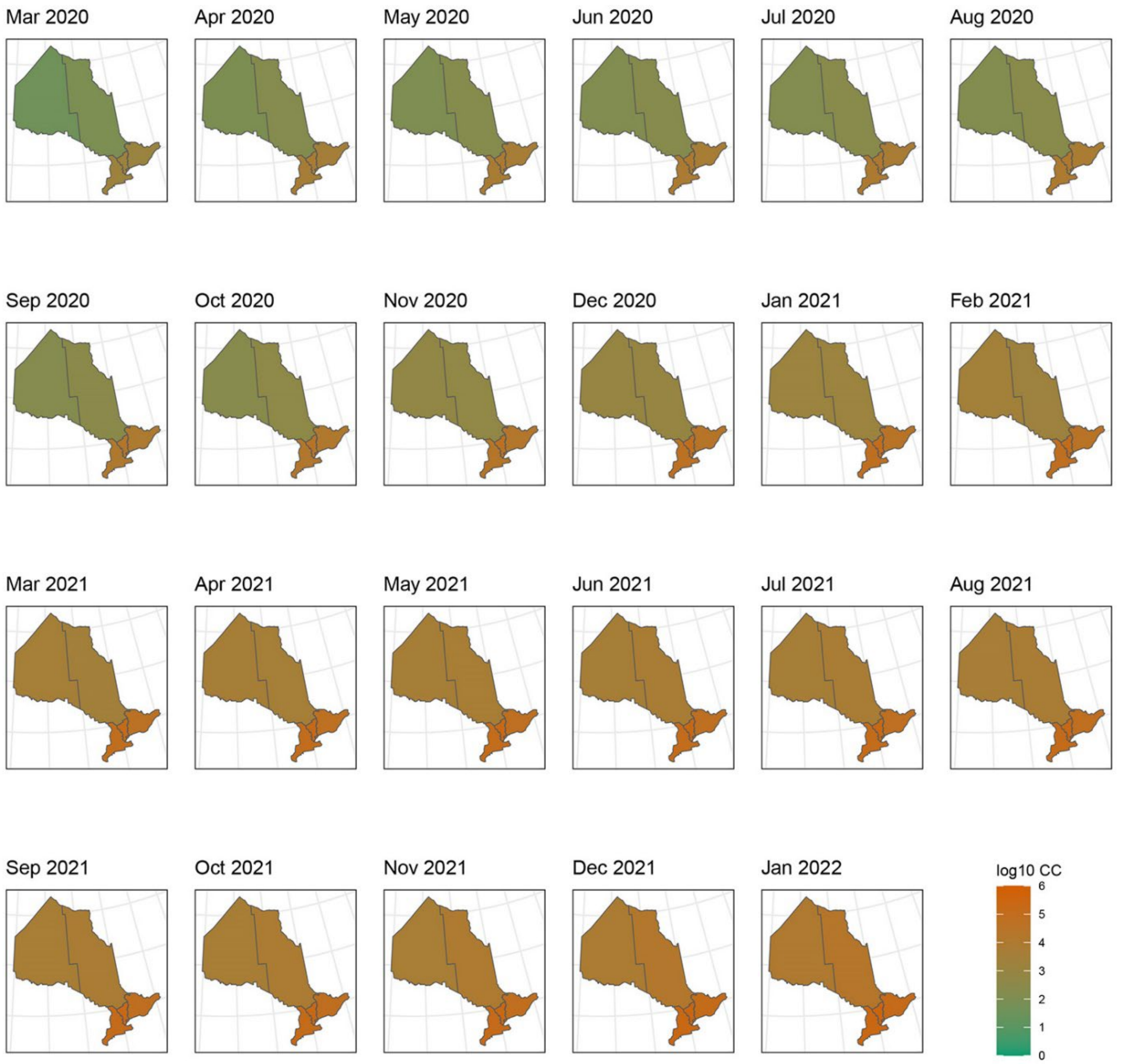

Appendix Figure 2. Maps of monthly $\log_{10}$-transformed cumulative case count by subprovincial public health regional area in Ontario, March 2020–January 2022. Log_{10} CC = $\log_{10}$-transformed cumulative case count.

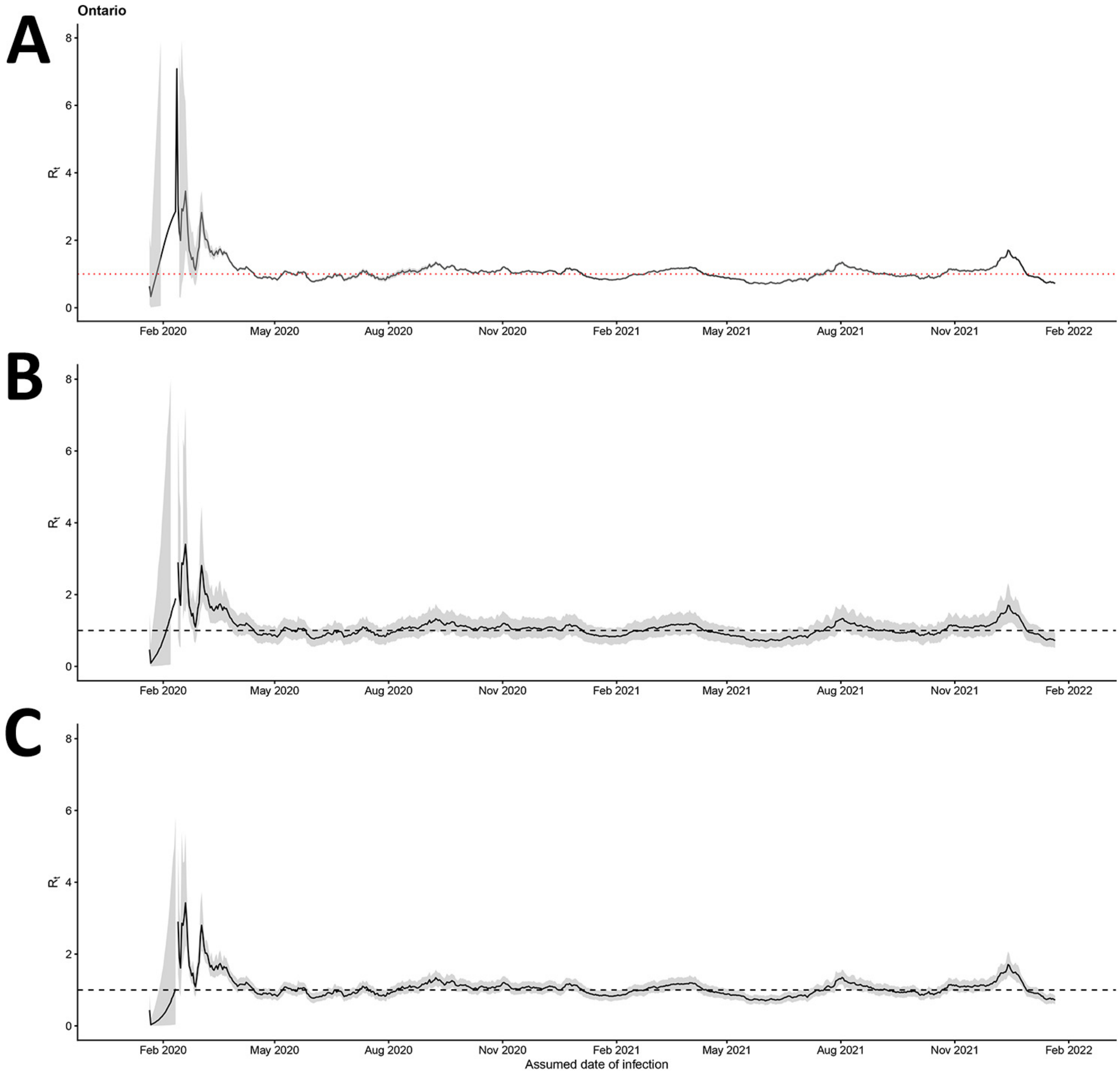

Appendix Figure 3. 7-day sliding-window median R_t using case count data as input (A), and the sensitivity analysis using the estimated infection count as input, assuming that on average B) 1/4 and C) 1/11 infected persons were reported as cases in Ontario.

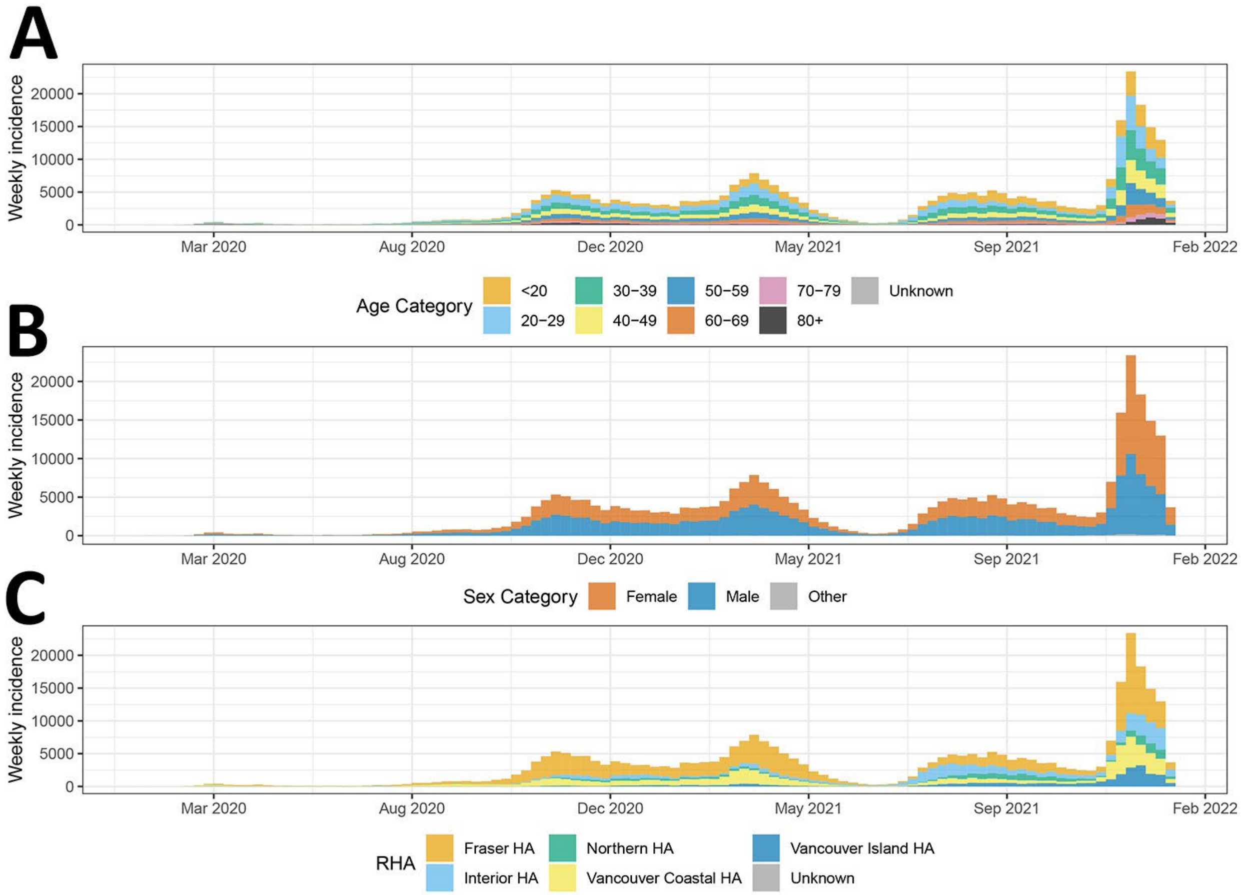

Appendix Figure 4. Descriptive weekly incidence curves by A) age, B) sex, and C) subprovincial public health regional area in British Columbia.

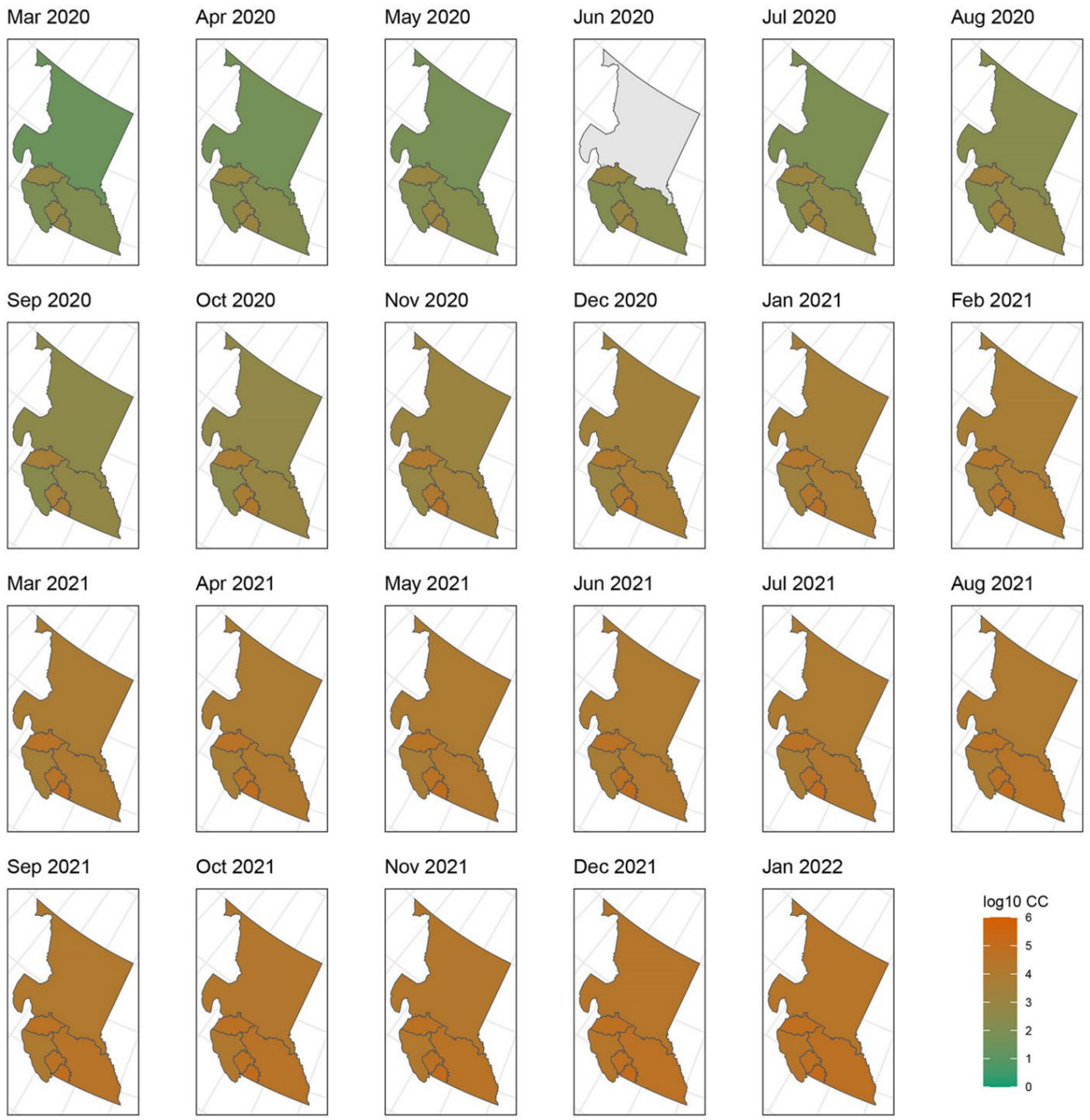

Appendix Figure 5. Maps of monthly $\log_{10}$-transformed cumulative case count by subprovincial regional health authority in British Columbia, March 2020–January 2022. Gray: no data available; $\log_{10}$ CC = $\log_{10}$-transformed cumulative case count.

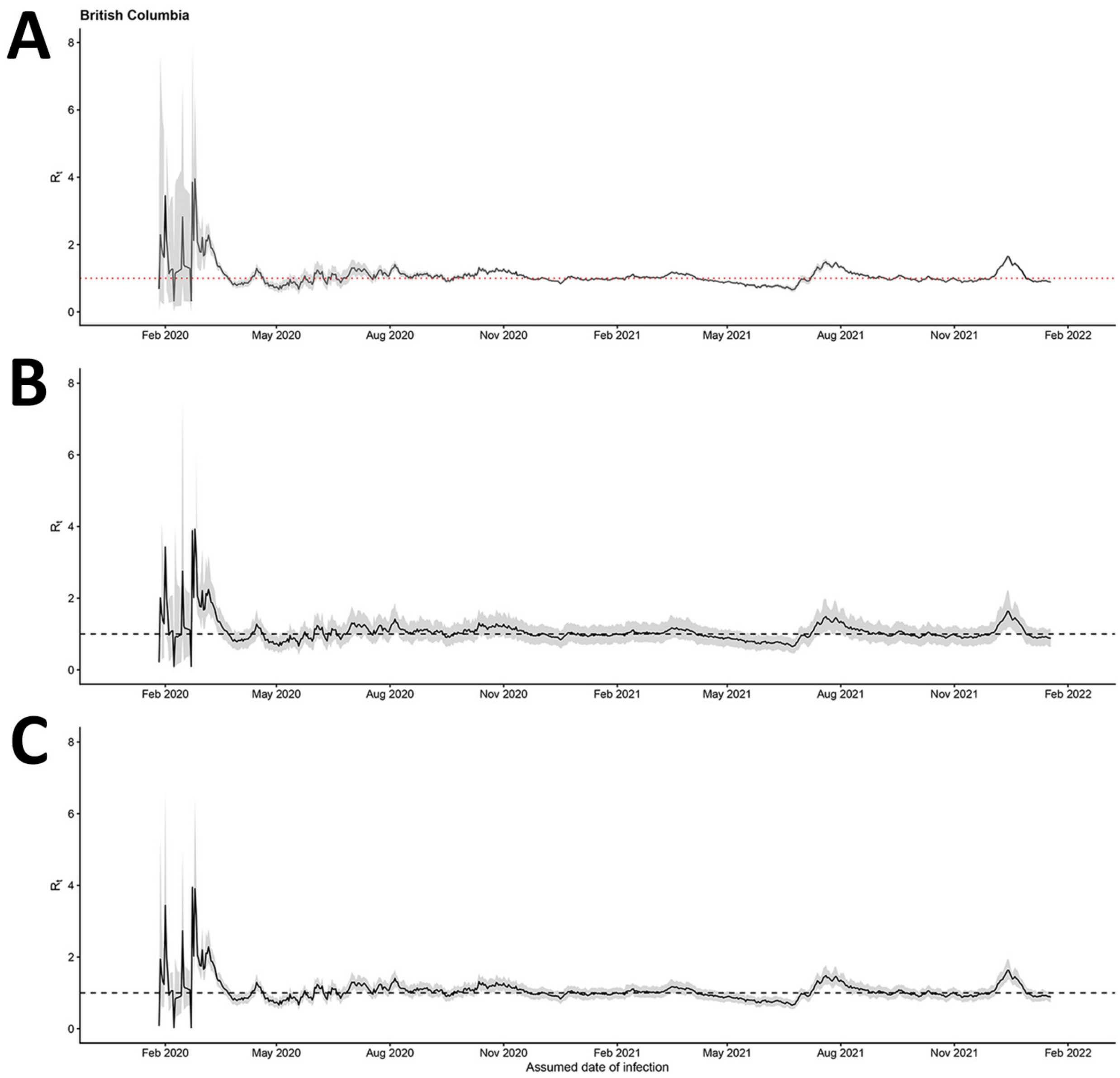

Appendix Figure 6. 7-day sliding-window median R_t using case count data as input (A), and the sensitivity analysis using the estimated infection count as input, assuming that on average B) 1/4 and C) 1/11 infected persons were reported as cases in British Columbia.

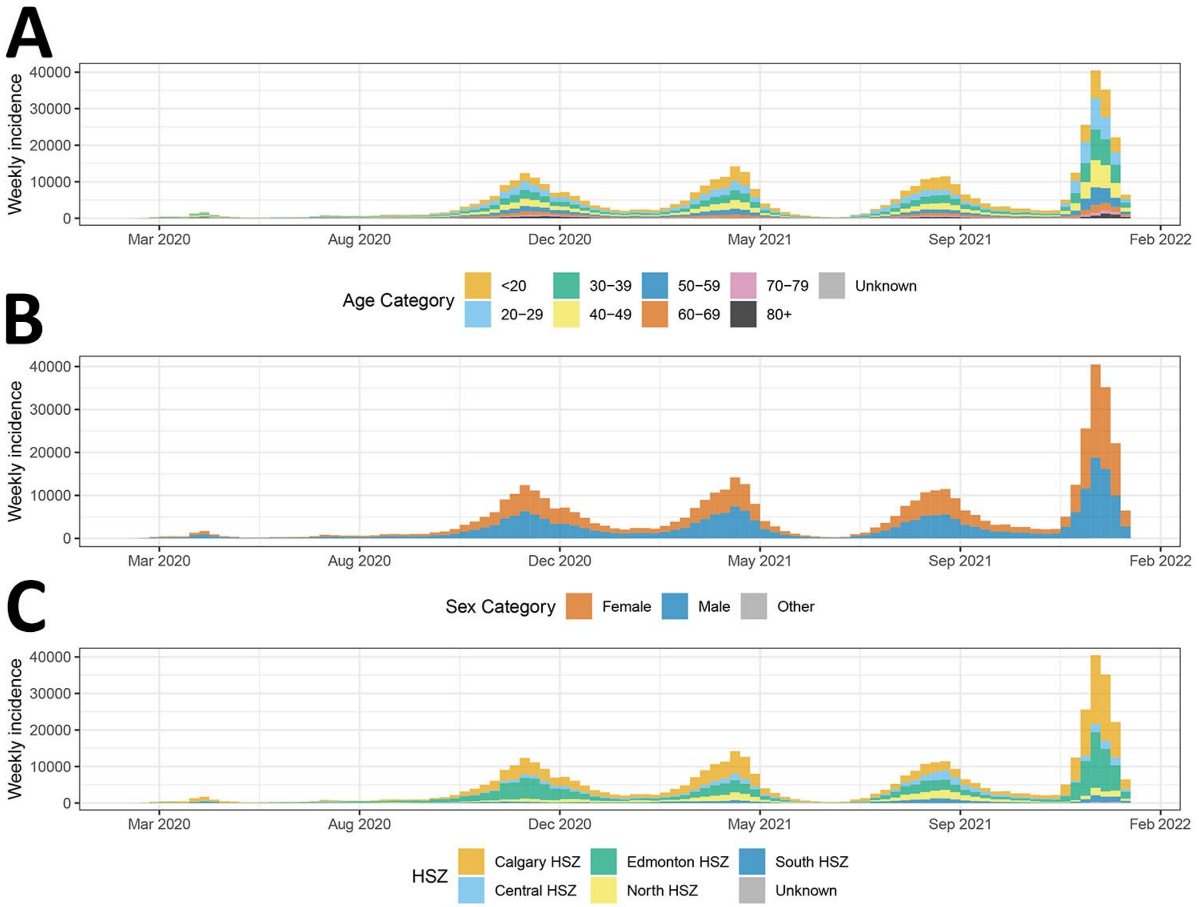

Appendix Figure 7. Descriptive weekly incidence curves A) age, B) sex, and C) subprovincial public health regional area in Alberta.

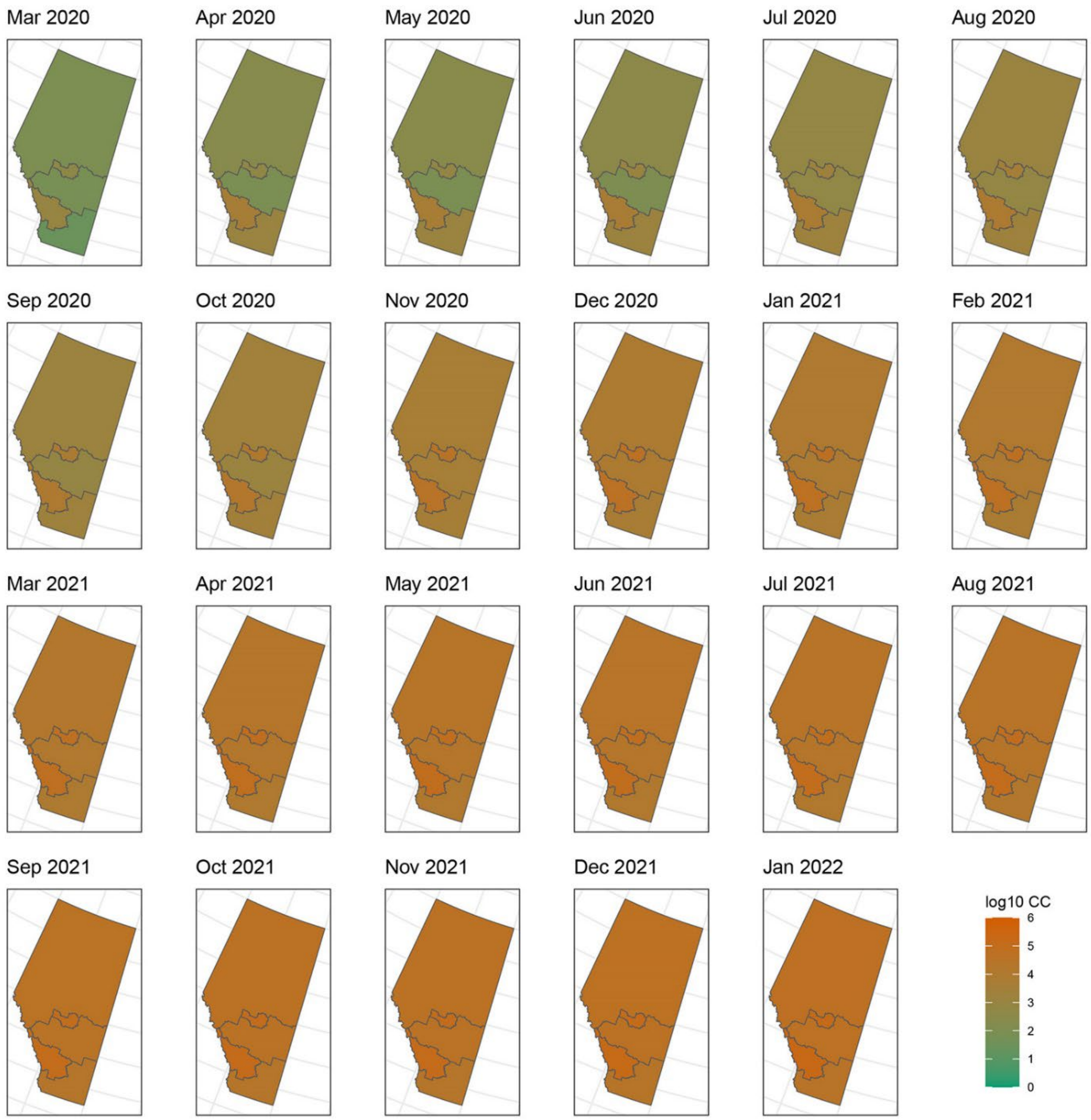

Appendix Figure 8. Maps of monthly $\log_{10}$-transformed cumulative case count by subprovincial health services zone in Alberta, March 2020–January 2022. $\log_{10}$ CC = $\log_{10}$-transformed cumulative case count.

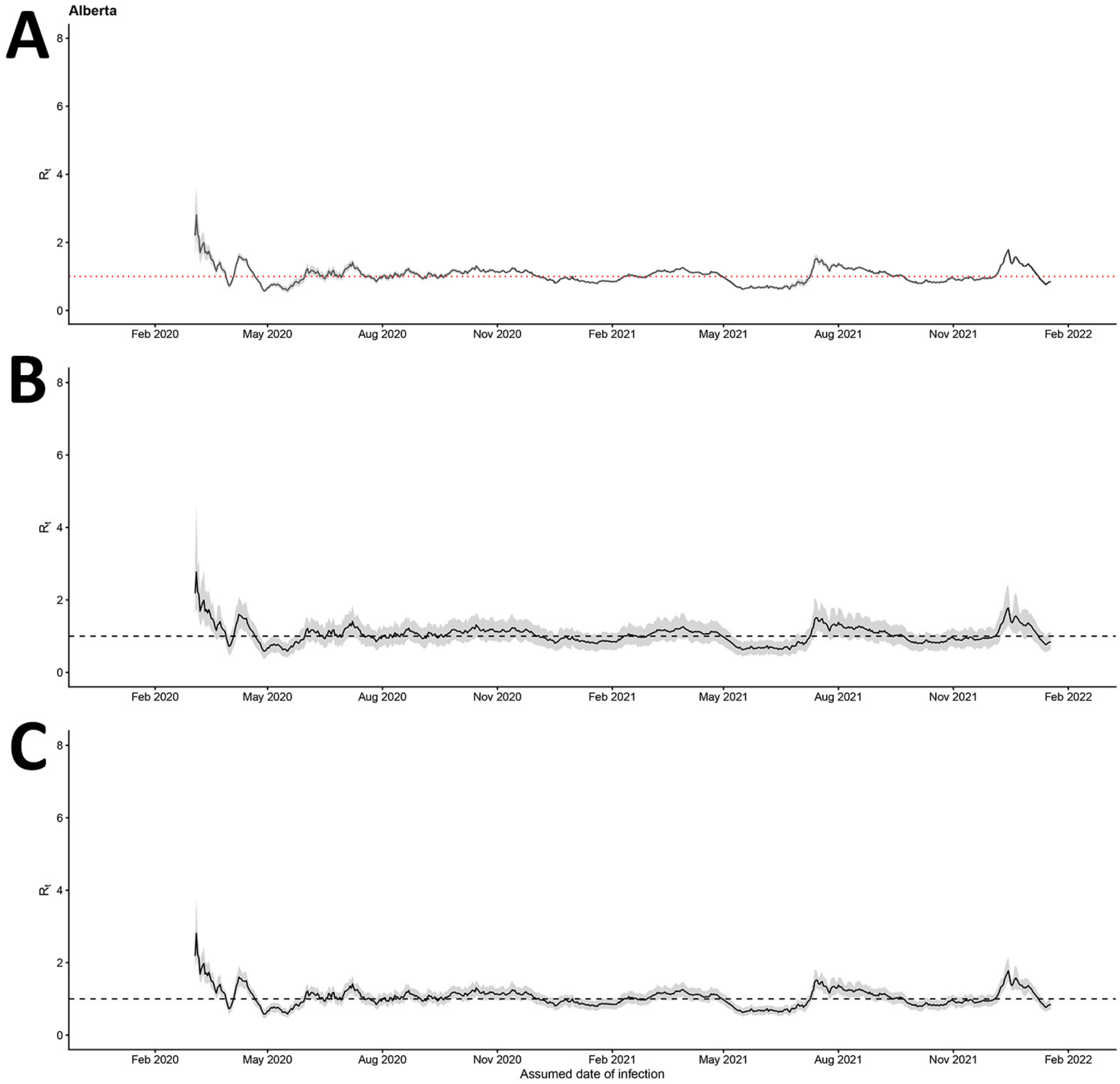

Appendix Figure 9. 7-day sliding-window median R_t using case count data as input (A), and the sensitivity analysis using the estimated infection count as input, assuming that on average B) 1/4 and C) 1/11 infected persons were reported as cases in Alberta.

Set Your Working Directory:

```
setwd("H:/Example/File/Path")
```

Load Required Packages:

Use: pacman::p_load() With the options: install = TRUE, update = getOption("pac_update") R will verify that the required packages are installed, if they are not then the install option will install the package, and the update option will update any packages to the newest version. This will also load the packages just like library() does.

```
pacman::p_load(
  cowplot,          # Streamlined ggplot2 theme and plot annotations
  dplyr,            # Data Manipulation
  EpiEstim,         # Rt estimation
  fmsb,             # fmsb::rateratio function is used to calculate IR
R and its CI
  ggplot2,          # Data visualizations
  ggpubr,           # General arguments description
  incidence2,       # Compute, handle and plot incidence of dated even
ts
  magrittr,
  scales,           # Graphically scales map data to aesthetics
  sf,
  tidyr)
```

READ PROVINCE FILES:

create data set for Ontario, British Columbia, and Alberta

```
# Ontario
linelistON <- read.csv("New_Ontario_linelist.csv", header = T)
# British Columbia
linelistBC <- read.csv("BCCDC_COVID19_Dashboard_Case_Details.csv", hea
der = T)
# Alberta
linelistAB <- read.csv("Alberta_Linelist.csv", header = T)
```

CLEAN LINELIST DATA:

verify sub-provincial public health units, standardize to NA, case count, 9-day shift for assumed date of infection

STANDARDIZED VARIABLES (linelist data sets):

- Date.Infected - assumed date of infection (9-day shift)
- Date.Reported - date reported of case

- Sex (Male, Female, Other)
- Age (<20, 20-29, 30-39, 40-49, 50-59, 60-69, 70-79, 80+, Unknown)
- Case.Count
- PH.Unit - sub-provincial public health unit (ON=PHRA, BC=RHA, AL=HSZ)

Ontario - data cleaning

```
# Sequence Case.count for all observations to 1
linelistON['Case.count'] <- seq(1)


# YYYY-MM-DD date formatting
linelistON$Case_Reported_Date <- as.Date(linelistON$Case_Reported_Date
, "%m/%d/%y")

# Create Date.Infected with a 9 day shift backwards
linelistON$Date.Infected <- linelistON$Case_Reported_Date-9


# Rename variables to above standards
linelistON <- linelistON %>%

  rename(Case.Count=Case.count,
         Date.Reported=Case_Reported_Date,
         Age=Age_Group,
         Sex=Client_Gender)

# Creation of PH.Unit variable based on reporting city
# Central
linelistON$PH.Unit <- ifelse(linelistON$Reporting_PHU_City == 'Barrie'
 |
                             linelistON$Reporting_PHU_City == 'Mississ
auga' |
                             linelistON$Reporting_PHU_City == 'Newmark
et' |
                             linelistON$Reporting_PHU_City == 'Oakvill
e',
                             'Central RA', 'Unknown')
# East
linelistON$PH.Unit <- ifelse(linelistON$Reporting_PHU_City == 'Bellevi
lle' |
                             linelistON$Reporting_PHU_City == 'Brockvi
lle' |
                             linelistON$Reporting_PHU_City == 'Cornwal
l' |
                             linelistON$Reporting_PHU_City == 'Kingsto
n' |
```

```
                             linelistON$Reporting_PHU_City == 'Ottawa'
 |
                             linelistON$Reporting_PHU_City == 'Peterbo
rough' |
                             linelistON$Reporting_PHU_City == 'Port Ho
pe' |
                             linelistON$Reporting_PHU_City == 'Whitby'
 |
                             linelistON$Reporting_PHU_City == 'Pembrok
e',
                             'East RA', linelistON$PH.Unit)
# North East
linelistON$PH.Unit <- ifelse(linelistON$Reporting_PHU_City == 'North B
ay' |
                             linelistON$Reporting_PHU_City == 'Sault S
te. Marie' |
                             linelistON$Reporting_PHU_City == 'Sudbury
' |
                             linelistON$Reporting_PHU_City == 'Timmins
' |
                             linelistON$Reporting_PHU_City == 'New Lis
keard',
                             'North-East RA', linelistON$PH.Unit)
# North West
linelistON$PH.Unit <- ifelse(linelistON$Reporting_PHU_City == 'Kenora'
 |
                             linelistON$Reporting_PHU_City == 'Thunder
 Bay',
                             'North-West RA', linelistON$PH.Unit)
# Toronto
linelistON$PH.Unit <- ifelse(linelistON$Reporting_PHU_City == 'Toronto
',
                             'Toronto RA', linelistON$PH.Unit)
#West
linelistON$PH.Unit <- ifelse(linelistON$Reporting_PHU_City == 'Chatham
' |
                             linelistON$Reporting_PHU_City == 'London'
 |
                             linelistON$Reporting_PHU_City == 'Owen So
und' |
                             linelistON$Reporting_PHU_City == 'Point E
dward' |
                             linelistON$Reporting_PHU_City == 'Stratfo
rd' |
                             linelistON$Reporting_PHU_City == 'St. Tho
mas' |
```

```
                                 linelistON$Reporting_PHU_City == 'Brantfo
rd' |
                                 linelistON$Reporting_PHU_City == 'Guelph'
 |
                                 linelistON$Reporting_PHU_City == 'Hamilto
n' |
                                 linelistON$Reporting_PHU_City == 'Simcoe'
 |
                                 linelistON$Reporting_PHU_City == 'Thorold
' |
                                 linelistON$Reporting_PHU_City == 'Waterlo
o' |
                                 linelistON$Reporting_PHU_City == 'Perth'
|
                                 linelistON$Reporting_PHU_City == 'Windsor
',
                                 'West RA', linelistON$PH.Unit)

# Re-coding of Age variables to standard
linelistON$Age <- ifelse(linelistON$Age == 'UNKNOWN', 'Unknown',
                             linelistON$Age)
linelistON$Age <- ifelse(linelistON$Age == '20s', '20-29',
                             linelistON$Age)
linelistON$Age <- ifelse(linelistON$Age == '30s', '30-39',
                             linelistON$Age)
linelistON$Age <- ifelse(linelistON$Age == '40s', '40-49',
                             linelistON$Age)
linelistON$Age <- ifelse(linelistON$Age == '50s', '50-59',
                             linelistON$Age)
linelistON$Age <- ifelse(linelistON$Age == '60s', '60-69',
                             linelistON$Age)
linelistON$Age <- ifelse(linelistON$Age == '70s', '70-79',
                             linelistON$Age)
linelistON$Age <- ifelse(linelistON$Age == '80s' | linelistON$Age == '
90+',
                             '80+',
                             linelistON$Age)

# Re-coding of Sex variables to standard
linelistON$Sex <- ifelse(linelistON$Sex == 'FEMALE', 'Female', linelis
tON$Sex)
linelistON$Sex <- ifelse(linelistON$Sex == 'MALE', 'Male', linelistON$
Sex)

# Create province variable
linelistON$Province <- "ON"
```

```
# Delete unnecessary variables
linelistON = subset(linelistON,
                    select = -c(Row_ID, Reporting_PHU, Reporting_PHU_C
ity))

# Reordering data set
linelistON <- linelistON[, c(5, 1, 3, 2, 4, 6, 7)]
```

British Columbia - data cleaning

```
# Sequence Case.count for all observations to 1
linelistBC['Case.Count'] <- seq(1)


# YYYY-MM-DD date formatting
linelistBC$Reported_Date <- as.Date(linelistBC$Reported_Date, "%Y-%m-%
d")

# Create Date.Infected with a 9 day shift backwards
linelistBC$Date.Infected <- linelistBC$Reported_Date-9


# Rename variables to above standards
linelistBC <- linelistBC %>%

  rename(Date.Reported=Reported_Date, Age=Age_Group, PH.Unit=HA)

# Re-coding of PH.Unit variables to include HA
linelistBC$PH.Unit <- ifelse(linelistBC$PH.Unit == 'Fraser',
                             'Fraser HA',
                             linelistBC$PH.Unit)
linelistBC$PH.Unit <- ifelse(linelistBC$PH.Unit == 'Interior',
                             'Interior HA',
                             linelistBC$PH.Unit)
linelistBC$PH.Unit <- ifelse(linelistBC$PH.Unit == 'Northern',
                             'Northern HA',
                             linelistBC$PH.Unit)
linelistBC$PH.Unit <- ifelse(linelistBC$PH.Unit == 'Vancouver Coastal'
,
                             'Vancouver Coastal HA',
                             linelistBC$PH.Unit)
linelistBC$PH.Unit <- ifelse(linelistBC$PH.Unit == 'Vancouver Island',

                             'Vancouver Island HA',
                             linelistBC$PH.Unit)
```

```
linelistBC$PH.Unit <- ifelse(linelistBC$PH.Unit == 'Out of Canada',
                             'Unknown',
                             linelistBC$PH.Unit)


# Re-coding of Age variables to standard
linelistBC$Age <- ifelse(linelistBC$Age == '<10' | linelistBC$Age == '
10-19',
                         '<20', linelistBC$Age)
linelistBC$Age <- ifelse(linelistBC$Age == '80-89' | linelistBC$Age ==
 '90+',
                         '80+', linelistBC$Age)

# Re-coding of Sex variables to standard
linelistBC$Sex <- ifelse(linelistBC$Sex == 'F', 'Female', linelistBC$S
ex)
linelistBC$Sex <- ifelse(linelistBC$Sex == 'M', 'Male', linelistBC$Sex
)
linelistBC$Sex <- ifelse(linelistBC$Sex == 'U', 'Other', linelistBC$Se
x)

# Create province variable
linelistBC$Province <- "BC"

# Delete unnecessary variables
linelistBC = subset(linelistBC, select = -c(Classification_Reported))


# Reordering data set
linelistBC <- linelistBC[, c(6, 1, 3, 4, 5, 2, 7)]
```

Alberta - data cleaning

```
# Sequence Case.count for all observations to 1
linelistAB['Case.Count'] <- seq(1)


# YYYY-MM-DD date formatting
linelistAB$Date.reported <- as.Date(linelistAB$Date.reported, "%m/%d/%
Y")

# Create Date.Infected with a 9 day shift backwards
linelistAB$Date.Infected <- linelistAB$Date.reported-9


# Rename variables to above standards
```

```
linelistAB <- linelistAB %>%

  rename(Date.Reported=Date.reported,
         Age=Age.group,
         PH.Unit=Alberta.Health.Services.Zone,
         Sex=Gender)

# Re-coding of PH.Unit variables to include HSZ
linelistAB$PH.Unit <- ifelse(linelistAB$PH.Unit == 'Calgary Zone', 'Ca
lgary HSZ',
                             linelistAB$PH.Unit)
linelistAB$PH.Unit <- ifelse(linelistAB$PH.Unit == 'Central Zone', 'Ce
ntral HSZ',
                             linelistAB$PH.Unit)
linelistAB$PH.Unit <- ifelse(linelistAB$PH.Unit == 'Edmonton Zone', 'E
dmonton HSZ',
                             linelistAB$PH.Unit)
linelistAB$PH.Unit <- ifelse(linelistAB$PH.Unit == 'North Zone', 'Nort
h HSZ',
                             linelistAB$PH.Unit)
linelistAB$PH.Unit <- ifelse(linelistAB$PH.Unit == 'South Zone', 'Sout
h HSZ',
                             linelistAB$PH.Unit)
linelistAB$PH.Unit[is.na(linelistAB$PH.Unit)] <- "Unknown"

# Re-coding of Age variables to standard
linelistAB$Age <- ifelse(linelistAB$Age == 'Under 20', '<20', linelist
AB$Age)
linelistAB$Age <- ifelse(linelistAB$Age == '20-29 years', '20-29', lin
elistAB$Age)
linelistAB$Age <- ifelse(linelistAB$Age == '30-39 years', '30-39', lin
elistAB$Age)
linelistAB$Age <- ifelse(linelistAB$Age == '40-49 years', '40-49', lin
elistAB$Age)
linelistAB$Age <- ifelse(linelistAB$Age == '50-59 years', '50-59', lin
elistAB$Age)
linelistAB$Age <- ifelse(linelistAB$Age == '60-69 years', '60-69', lin
elistAB$Age)
linelistAB$Age <- ifelse(linelistAB$Age == '70-79 years', '70-79', lin
elistAB$Age)
linelistAB$Age <- ifelse(linelistAB$Age == '80+ years', '80+', linelis
tAB$Age)

# Re-coding of Sex variables to standard
linelistAB$Sex <- ifelse(linelistAB$Sex == 'Unknown', 'Other', linelis
tAB$Sex)
```

```
linelistAB$Sex[is.na(linelistAB$Sex)] <- "Other"

# Create province variable
linelistAB$Province <- "AB"

# Delete unnecessary variables
linelistAB = subset(linelistAB, select = -c(...1, Case.status))


# Reordering data set
linelistAB <- linelistAB[, c(6, 1, 3, 4, 5, 2, 7)]
```

DESCRIPTIVE ANALYSIS: includes Figures 1-3 and Supplemental Figures S1, S2, S5, S6, S9, and S10

Ontario

ON Figure S1

```
# Create incidence
epi_day_ON <- incidence2::incidence(  # create incidence object (incid
ence2 package)
  x = linelistON,                     # dataset
  date_index = Date.Infected,         # date column
  interval = "week"                   # date grouping interval
)

# Create color-blind friendly color scales for Age, Sex, and PHU figur
es
# colors are identified with hex codes
cbpAge <- c("#E69F00", "#56B4E9", "#009E73", "#F0E442", "#0072B2", "#D
55E00",
            "#CC79A7", "#000000", "#999999")
cbpSex <- c("#D55E00", "#0072B2", "#999999")
cbpPHU <- c("#E69F00", "#56B4E9", "#009E73", "#F0E442", "#0072B2", "#D
55E00")
cbpPHU1 <- c("#E69F00", "#56B4E9", "#009E73", "#F0E442", "#0072B2", "#
999999")

### Weekly Incidence by Age Category
age_outbreak_ON <- incidence(
  linelistON,                              # dataset
  date_index = Date.Infected,              # date column
  interval = "week",                       # Monday weekly aggregation
 of cases
```

```
  groups = Age,                              # age_cat is set as a group
  na_as_group = TRUE)                        # missing values assigned t
heir own group

# plot the grouped incidence object - age
(d_3_ON<-plot(
  age_outbreak_ON,                           # incidence object with age
_cat as group
  fill = Age,                                # age_cat is used for bar f
ill color
  centre_dates = FALSE,                      # Create date label
  date_format = "%b %Y") +                   # Format date label
  scale_fill_manual(values = cbpAge) +       # fill colors must be set b
eforehand
  labs(fill = "Age Category") +              # create label
  theme(legend.position = "bottom"))         # specify legend location

### Weekly Incidence by Sex
sex_outbreak_ON <- incidence(
  linelistON,                                # dataset
  date_index = Date.Infected,                # date column
  interval = "week",                         # Monday weekly aggregation
 of cases
  groups = Sex,                              # age_cat is set as a group
  na_as_group = TRUE)                        # missing values assigned t
heir own group

# plot the grouped incidence object - sex
(d_5_ON<-plot(
  sex_outbreak_ON,                        # incidence object with age_ca
t as group
  fill = Sex,                                # age_cat is used for bar f
ill color
  centre_dates = FALSE,                      # Create date label
  date_format = "%b %Y") +                   # Format date label
  scale_fill_manual(values = cbpSex) +       # fill colors must be set b
eforehand
  labs(fill = "Sex Category") +           # create label
  theme(legend.position = "bottom"))         # specify legend location

### Weekly Incidence by PHRAs
location_outbreak_ON <- incidence(
  linelistON,                                # dataset
  date_index = Date.Infected,                # date column
  interval = "week",                         # Monday weekly aggregation
 of cases
```

```
  groups = PH.Unit,                            # age_cat is set as a group
  na_as_group = TRUE)                          # missing values assigned t
heir own group

# rename PH.Unit - add RA
location_outbreak_ON$PH.Unit <- factor(location_outbreak_ON$PH.Unit,
                                       levels = c("Central RA", "East
RA",
                                                  "North-East RA", "No
rth-West RA",
                                                  "Toronto RA", "West
RA",
                                                  "Unknown"))
# plot the grouped incidence object - PHRAs
(d_7_ON<- plot(
  location_outbreak_ON,                        # incidence object with age
_cat as group
  fill = PH.Unit,                              # age_cat is used for bar f
ill color
  centre_dates = FALSE,                        # Create date label
  date_format = "%b %Y") +                     # Format date label
  scale_fill_manual(values = cbpPHU) +         # fill colors must be set b
eforehand
  labs(fill = "PHRA") +                        # create label
  theme(legend.position = "bottom"))           # specify legend location

### Export 3-panel figure S1
prefix<-"ON_EpiCurve_Age_Sex_PHU"                      # name for figure
tiffname<-paste0(prefix, "_3panel.tif")                  # paste file ty
pe to name
tiff(tiffname,height=8, width=10, units="in",res=300)  # specify figur
e aspects
pdf(file="ON_EpiCurve_Age_Sex_PHU.pdf", width=10, height=8) # pdf file
 export
gridExtra::grid.arrange(d_3_ON,d_5_ON,d_7_ON, nrow=3)  # specify figur
e arrangement
dev.off()
```

Creation of map file for Ontario, British Columbia, and Alberta

```
# Creation of map names using HR_UID
mapname <- st_read("HR_000a18a_e.shp")

### Update Ontario PH_Unit Names
# Central
mapname$PH_Unit[mapname$HR_UID == "3553"] <- "Central RA"
```

```
mapname$PH_Unit[mapname$HR_UID == "3560"] <- "Central RA"
mapname$PH_Unit[mapname$HR_UID == "3570"] <- "Central RA"
mapname$PH_Unit[mapname$HR_UID == "3536"] <- "Central RA"
# East
mapname$PH_Unit[mapname$HR_UID == "3538"] <- "East RA"
mapname$PH_Unit[mapname$HR_UID == "3543"] <- "East RA"
mapname$PH_Unit[mapname$HR_UID == "3558"] <- "East RA"
mapname$PH_Unit[mapname$HR_UID == "3541"] <- "East RA"
mapname$PH_Unit[mapname$HR_UID == "3551"] <- "East RA"
mapname$PH_Unit[mapname$HR_UID == "3555"] <- "East RA"
mapname$PH_Unit[mapname$HR_UID == "3535"] <- "East RA"
mapname$PH_Unit[mapname$HR_UID == "3530"] <- "East RA"
mapname$PH_Unit[mapname$HR_UID == "3557"] <- "East RA"
# North East
mapname$PH_Unit[mapname$HR_UID == "3547"] <- "North-East RA"
mapname$PH_Unit[mapname$HR_UID == "3526"] <- "North-East RA"
mapname$PH_Unit[mapname$HR_UID == "3561"] <- "North-East RA"
mapname$PH_Unit[mapname$HR_UID == "3556"] <- "North-East RA"
mapname$PH_Unit[mapname$HR_UID == "3563"] <- "North-East RA"
# North West
mapname$PH_Unit[mapname$HR_UID == "3562"] <- "North-West RA"
mapname$PH_Unit[mapname$HR_UID == "3549"] <- "North-West RA"
# Toronto
mapname$PH_Unit[mapname$HR_UID == "3595"] <- "Toronto RA"
# West
mapname$PH_Unit[mapname$HR_UID == "3527"] <- "West RA"
mapname$PH_Unit[mapname$HR_UID == "3566"] <- "West RA"
mapname$PH_Unit[mapname$HR_UID == "3537"] <- "West RA"
mapname$PH_Unit[mapname$HR_UID == "3534"] <- "West RA"
mapname$PH_Unit[mapname$HR_UID == "3546"] <- "West RA"
mapname$PH_Unit[mapname$HR_UID == "3565"] <- "West RA"
mapname$PH_Unit[mapname$HR_UID == "3540"] <- "West RA"
mapname$PH_Unit[mapname$HR_UID == "3544"] <- "West RA"
mapname$PH_Unit[mapname$HR_UID == "3533"] <- "West RA"
mapname$PH_Unit[mapname$HR_UID == "3542"] <- "West RA"
mapname$PH_Unit[mapname$HR_UID == "3539"] <- "West RA"
mapname$PH_Unit[mapname$HR_UID == "3568"] <- "West RA"
mapname$PH_Unit[mapname$HR_UID == "3554"] <- "West RA"
mapname$PH_Unit[mapname$HR_UID == "3575"] <- "West RA"

### Update British Columbia PH_Unit Names
# These are updated here since we are using one map file for all 3 pro
vinces
# Fraser
mapname$PH_Unit[mapname$HR_UID == "5921"] <- "Fraser HA"
mapname$PH_Unit[mapname$HR_UID == "5922"] <- "Fraser HA"
```

```r
mapname$PH_Unit[mapname$HR_UID == "5923"] <- "Fraser HA"
# Interior
mapname$PH_Unit[mapname$HR_UID == "5911"] <- "Interior HA"
mapname$PH_Unit[mapname$HR_UID == "5912"] <- "Interior HA"
mapname$PH_Unit[mapname$HR_UID == "5913"] <- "Interior HA"
mapname$PH_Unit[mapname$HR_UID == "5914"] <- "Interior HA"
# Northern
mapname$PH_Unit[mapname$HR_UID == "5951"] <- "Northern HA"
mapname$PH_Unit[mapname$HR_UID == "5952"] <- "Northern HA"
mapname$PH_Unit[mapname$HR_UID == "5953"] <- "Northern HA"
# Vancouver Coastal
mapname$PH_Unit[mapname$HR_UID == "5931"] <- "Vancouver Coastal HA"
mapname$PH_Unit[mapname$HR_UID == "5932"] <- "Vancouver Coastal HA"
mapname$PH_Unit[mapname$HR_UID == "5933"] <- "Vancouver Coastal HA"
# Vancouver Island
mapname$PH_Unit[mapname$HR_UID == "5941"] <- "Vancouver Island HA"
mapname$PH_Unit[mapname$HR_UID == "5942"] <- "Vancouver Island HA"
mapname$PH_Unit[mapname$HR_UID == "5943"] <- "Vancouver Island HA"

### Update Alberta PH_Unit Names
# These are updated here since we are using one map file for all 3 pro
vinces
mapname$PH_Unit[mapname$ENGNAME == "Calgary Zone"]  <-"Calgary HSZ"
mapname$PH_Unit[mapname$ENGNAME == "Central Zone"]  <-"Central HSZ"
mapname$PH_Unit[mapname$ENGNAME == "Edmonton Zone"] <-"Edmonton HSZ"
mapname$PH_Unit[mapname$ENGNAME == "North Zone"]    <-"North HSZ"
mapname$PH_Unit[mapname$ENGNAME == "South Zone"]    <-"South HSZ"

# Drop all PH_Unit that are NA
mapname <- mapname %>% drop_na(PH_Unit)
```

ON Figure S2

Monthly log10 incidence by PHRAs

```r
### Monthly log10 incidence by PHRAs
mapsON <- linelistON[c("Province", "PH.Unit", "Date.Infected")]
mapsON$X <- 1

# Unique ID creation M2001 = January 2020 | M2105 = May 2021
mapsON$mo <- paste0("M", format(as.Date(mapsON$Date.Infected), "%y%m")
)

# Calculate the cumulative case count by PH.Unit
mapsON<-mapsON %>%
  group_by(Province, PH.Unit) %>%
  arrange(Province, PH.Unit, Date.Infected)  %>%
```

```
  mutate(cumcase_PHRA = cumsum(X))

# Collapse line list data to monthly data
mapsON <- mapsON %>%
  group_by(Province, PH.Unit, mo) %>%
  filter(cumcase_PHRA == max(cumcase_PHRA)) %>%
  arrange(Province, PH.Unit, mo)

# Delete unnecessary variables
mapsON = subset(mapsON, select = -c(X, Date.Infected))

# Transform data from long to wide format
mapsON<-spread(mapsON, mo, cumcase_PHRA)

# Merge geometry polygons based on PH_Unit
# Source: https://www.jla-data.net/eng/merging-geometry-of-sf-objects-
in-r/
ON_map <- mapname %>%
  group_by(PH_Unit) %>%
  summarise()

# Identify ON map regions
ON_PHRU <- c("Central RA", "East RA", "North-West RA",
             "North-East RA", "Toronto RA", "West RA")

# Select map regions in ON only
ON_map<-ON_map %>%
  filter(PH_Unit %in% (ON_PHRU))

ggplot()+
  geom_sf(data=ON_map, aes(fill=PH_Unit)) #Confirm Map of the 6 PH_uni
t

ON_map1 <- merge(ON_map, mapsON, by.x = 'PH_Unit', by.y = 'PH.Unit', a
ll.x=T)
View(ON_map1) #PH_unit, geometry, M2001, M2002, etc.

### Testing: plot the first plot for March 2020
ggplot()+
  geom_sf(data=ON_map1, aes(fill=log10(M2003))) +
  theme_bw() +
  coord_sf(default_crs = sf::st_crs(4326)) +
  scale_fill_continuous(low="#009E73",
                        high="#D55E00",
                        guide="colorbar",
                        na.value="gray90", limits=c(0, 6)) +
```

```
  theme(axis.title.x = element_blank(),  axis.title.y = element_blank(
),
        axis.text.x=element_blank(),             # remove x-axis labe
ls
        axis.ticks.x=element_blank(),            # remove x-axis tick
s
        axis.text.y=element_blank(),             # remove y-axis labe
ls
        axis.ticks.y=element_blank(),            # remove y-axis tick
s
        legend.position = "none",
        text=element_text(size=8))+
  ggtitle("Mar 2020")

# To simplify the code, first save your theme modifications and
# scale modifications as 2 objects to be re-used.
mytheme<-  theme(axis.title.x = element_blank(),  # remove x-axis titl
e
                 axis.title.y = element_blank(),  # remove y-axis titl
e
                 axis.text.x  = element_blank(),  # remove x-axis labe
ls
                 axis.text.y  = element_blank(),  # remove y-axis labe
ls
                 axis.ticks.x = element_blank(),  # remove x-axis tick
s
                 axis.ticks.y = element_blank(),  # remove y-axis tick
s
                 legend.position = "none",
                 text=element_text(size=8))
myscale<- scale_fill_continuous(low="#009E73",
                                high="#D55E00",
                                guide="colorbar",
                                na.value="gray90",
                                limits=c(0, 6))

### Testing: it works!
ggplot()+
  geom_sf(data=ON_map1, aes(fill=log10(M2003))) +
  theme_bw() +
  coord_sf(default_crs = sf::st_crs(4326)) +
  myscale +
  mytheme +
  ggtitle("Mar 2020")

### To prepare for a loop
```

```
(list1ON<-colnames(mapsON[,c(4:27)]))
# [1] "M2003" "M2004" "M2005" "M2006" "M2007" "M2008" "M2009" "M2010"
"M2011" "M2012"
# [11] "M2101" "M2102" "M2103" "M2104" "M2105" "M2106" "M2107" "M2108"
 "M2109" "M2110"
# [21] "M2111" "M2112" "M2201"

(list2ON<-stringr::str_sub(list1ON, start=2L,end=3L))
(list3ON<-stringr::str_sub(list1ON, start=4L,end=5L))
(list2aON<-paste("20", list2ON, sep=""))
(list3aON<-as.integer(list3ON))

# Source: https://datacornering.com/how-to-get-the-month-name-from-the
-number-in-r/

month.name[list3aON]
month.abb[list3aON]
paste(month.abb[list3aON], list2aON, sep=" ")

(title_vectorON <- paste(month.abb[list3aON], list2aON, sep=" "))
# [1] "Mar 2020" "Apr 2020" "May 2020" "Jun 2020" "Jul 2020" "Aug 2020
" "Sep 2020"
# [8] "Oct 2020" "Nov 2020" "Dec 2020" "Jan 2021" "Feb 2021" "Mar 2021
" "Apr 2021"
# [15] "May 2021" "Jun 2021" "Jul 2021" "Aug 2021" "Sep 2021" "Oct 202
1" "Nov 2021"
# [22] "Dec 2021" "Jan 2022"

### Testing using list1 and title_vector
ggplot()+
  geom_sf(data=ON_map1, aes(fill=log10(.data[[list1ON[1]]]))) +
  theme_bw() +
  coord_sf(default_crs = sf::st_crs(4326)) +
  myscale +
  mytheme +
  ggtitle(title_vectorON[1])

# Write a function
ON_individual_plot <- function(index,
                               data,
                               list1=list1ON,
                               title_vector=title_vector,
                               myscale=myscale,
                               mytheme=mytheme){
  ggplot()+
    geom_sf(data=data, aes(fill=log10(.data[[list1ON[index]]]))) +
```

```r
    theme_bw() +
    coord_sf(default_crs = sf::st_crs(4326)) +
    myscale +
    mytheme +
    ggtitle(title_vectorON[index])
}

length(title_vectorON)
#[1] 24
(indicesON<-c(1:24))

#Create empty list of length same as the length of indices
ON_plots_list<-vector(mode='list', length=length(indicesON))

# testing
ON_individual_plot(1, ON_map1, list1ON, title_vectorON, myscale, mythe
me)
ON_individual_plot(4, ON_map1, list1ON, title_vectorON, myscale, mythe
me)

### A for-loop using the ON_individual_plot function
### to create the individual plots
for (i in indicesON){
  ON_plots_list[[i]]<-ON_individual_plot(i,
                                         ON_map1,
                                         list1ON,
                                         title_vectorON,
                                         myscale,
                                         mytheme)
}

### Export Figure S2
legend_on <- get_legend(ON_plots_list[[length(title_vectorON)]]+
                          theme(legend.position="right")+
                          labs(fill="log10 CC"))
prefix<-"ON_MonMap"
tiffname<-paste0(prefix, ".tif")
tiff(tiffname,height=8, width=8, units='in', res=300)
pdf(file="ON_MonMap.pdf", width=8, height=8)
gridExtra::grid.arrange(ON_plots_list[[2]],
                        ON_plots_list[[3]],ON_plots_list[[4]],
                        ON_plots_list[[5]],ON_plots_list[[6]],
                        ON_plots_list[[7]],ON_plots_list[[8]],
                        ON_plots_list[[9]],ON_plots_list[[10]],
                        ON_plots_list[[11]],ON_plots_list[[12]],
                        ON_plots_list[[13]],ON_plots_list[[14]],
```

```
                        ON_plots_list[[15]],ON_plots_list[[16]],
                        ON_plots_list[[17]],ON_plots_list[[18]],
                        ON_plots_list[[19]],ON_plots_list[[20]],
                        ON_plots_list[[21]],ON_plots_list[[22]],
                        ON_plots_list[[23]],ON_plots_list[[24]],
                        legend_on, ncol=6, nrow=4)
dev.off()
```

ON Figure S4

3-panel sensitivity figure

```
# Create dataset of case counts by day
ONcases <- incidence2::incidence(linelistON, date_index = Date.Infecte
d) %>%
  tidyr::complete(date_index = seq.Date(# ensure all dates are represe
nted
    from = min(date_index, na.rm = T),
    to = max(date_index, na.rm=T),
    by = "day"),
    fill = list(count = 0)) %>%         # convert NA counts to 0
  rename(I = count,                      # rename to names expected by
estimateR
         dates = date_index)

# Set the configuration for estimate_R function
# for the mean and standard deviation for serial interval distribution
config_lit <- make_config(
  mean_si = 4.6, # mean of serial interval = 4.60
  std_si = 5.55  # standard deviation of serial interval = 5.55
)

# Estimate R for - Epi curves, R, and SI distribution
epiestim_res_litON <- estimate_R(
  incid = ONcases,
  method = "parametric_si", # we use the parametric method
  config = config_lit
)

### median Rt
dfON<-data.frame(epiestim_res_litON$dates[8:length(epiestim_res_litON$
dates)],
               epiestim_res_litON$R$`Median(R)`,
               epiestim_res_litON$R$`Quantile.0.025(R)`,
               epiestim_res_litON$R$`Quantile.0.975(R)`)
colnames(dfON)<-c("dates", "median", "lower", "upper")
w_ON <-ggplot(data=dfON) +
```

```
  geom_line(aes(x=dates, y=median))+
  geom_ribbon(aes(x=dates, ymin=lower, ymax=upper),
              fill="grey70", alpha=0.5)

d_ON <-w_ON+theme_classic()+
  labs(title="Ontario",x=" ",y=expression(R[t]))+
  theme(legend.position="bottom")+
  scale_x_date(breaks=date_breaks("3 months"),
               labels=date_format("%b %Y"),
               limits=c(as.Date("2020-01-01"), as.Date("2022-02-01")))
+
  scale_y_continuous(limits = c(0,8))+
  geom_hline(yintercept=1.0, color="red",linetype="dotted") +
  theme_cowplot(font_size = 10)+
  theme(legend.position = "bottom")

# Removing title and Y-axis limit for manuscript Figure 1
d_ON1 <-w_ON+theme_cowplot(font_size = 12)+
  labs(title="",x=" ",y=expression(R[t]))+
  theme(legend.position="bottom")+
  scale_x_date(breaks=date_breaks("3 months") , labels=date_format("%b
 %Y"),
               limits=c(as.Date("2019-11-29"), as.Date("2022-01-27")))
+
  scale_y_continuous(limits = c(0,8))+
  geom_hline(yintercept=1.0, color="red",linetype="dotted") +
  theme(legend.position = "bottom")


### Multiplier: Poisson distribution with a mean of 4
set.seed(1234)
# Generating a matrix of random numbers: each column (x-axis) is a day
, and
# each row (y-axis) is an iteration (e.g., you may have n=1000 simulat
ions)
poisON<-matrix(rpois(n=(nrow (ONcases)*1000), lambda=4),
               ncol = nrow(ONcases))

# Renaming matrix columns and rows
colnames(poisON) <- colnames(poisON, do.NULL = FALSE,
                             prefix = "day.")
rownames(poisON) <- rownames(poisON, do.NULL = FALSE,
                             prefix = "iteration.")

# Estimated number of infections
# Multiply the daily new case count by each row of the matrix to gener
```

```
ate n
# simulations of the epi curve (estimated number of infections).
# Apply sweep operation that multiplies row by column multiplication
# (MARGIN=2) between poisON and ONcases$I
save.resON<-(sweep(poisON, MARGIN=2, ONcases$I, `*`))

# Calculate the median values for each day (by column)
# save.resON = simulated data (estimated number of infections)
# calculate the median values for each day (by column)
EstInf_too1ON<-apply(save.resON,2,median)

# Calculating 95% CrI
# save.resON: each column is a day; each row is one iteration
# apply by column (2)
EstInf_lowerON <- apply(save.resON,2, quantile, probs=0.025) #2.5 perc
entile
EstInf_upperON <- apply(save.resON,2, quantile, probs=0.975)#97.5 perc
entile

# Create dataframe for the medians and CrI
EstInf_dfON <- data.frame(EstInf_too1ON,
                          EstInf_lowerON,
                          EstInf_upperON)

# Preparing data for EpiEstim
newdataON<-as.matrix.data.frame(save.resON)

# Run EpiEstim::estimate_R on each of these simulated epi curves.
test_resON<-vector(mode="list", length = nrow(newdataON)) # list

for(i in 1:nrow(newdataON)){
  test_resON [[i]]<- estimate_R(newdataON [i,],
                      method="parametric_si",  # we use the parametric
 method
                      config=config_lit)
}

r_m0  <-test_resON
n     <-10       # The number of values you want to randomly generate
Sample<-ONcases

# Generate 10 random values from the posterior R distribution
# for each time step for each hypothetical time series
sample_r_m0<-EpiEstim::sample_posterior_R(r_m0[[1]],
                                          n      = n,
                                          window = 1L)
```

```
for(v in 2:length(r_m0)){
  sample_r_m0<-append(sample_r_m0,
                      sample_posterior_R(r_m0[[v]],
                                         n      = n,
                                         window = 1L))
}
sample_r_m0_df<-sample_r_m0
for(window_number in 2:length(r_m0[[1]]$R$t_start)){
  sample_r_m0<-sample_posterior_R(r_m0[[1]],
                                  n      = n,
                                  window = window_number)
  for(v in 2:length(r_m0)){
    sample_r_m0<-append(sample_r_m0,
                        sample_posterior_R(r_m0[[v]],
                                           n      = n,
                                           window = window_number))
  }
  sample_r_m0_df<-rbind(sample_r_m0_df, sample_r_m0)
}

sample_r_m0_df<-as.data.frame(sample_r_m0_df)

sample_r_m0_df<-cbind(Sample$dates[(r_m0[[1]]$R$t_end[1]):nrow(Sample)
],
                      sample_r_m0_df)

colnames(sample_r_m0_df)[1]<-"dates"


sample_r_m0_df <- sample_r_m0_df %>%
  rowwise() %>%
  mutate(Median=median(c_across(contains("V")))) %>%
  mutate(Lower=quantile(c_across(contains("V")), probs=0.025,
                        na.rm=F, names=F)) %>%
  mutate(Upper=quantile(c_across(contains("V")), probs=0.975,
                        na.rm=F, names=F)) %>%
  ungroup()  %>%
  dplyr::select(dates, Median, Lower, Upper, everything())

ON4_sample_r_m0_df <- sample_r_m0_df

M4_p1 <- ggplot(data    = ON4_sample_r_m0_df,
             mapping = aes(x = dates,
                           y = Median))+
  geom_line(col="black") +
  geom_ribbon(aes(ymin = Lower,
```

```
                   ymax = Upper),
               alpha = 0.2)

M4_g1 <- M4_p1 + labs(y = expression(R[t]),
                 x = "") +
  geom_hline(yintercept = 1,
             linetype   = 2) +
  theme_classic() +
  theme_cowplot(font_size = 10)+
  scale_x_date(breaks = date_breaks("3 months"),
               labels = date_format("%b %Y"),
               limits = c(as.Date("2020-01-01"),
                          as.Date("2022-02-01")))
M4_g1
(M4_g1b<-M4_g1+scale_y_continuous(limits=c(0,8)))


### Multiplier: Poisson distribution with a mean of 11
# Generating a matrix of random numbers: each column (x-axis) is a day
, and
# each row (y-axis) is an iteration (e.g., you may have n=1000 simulat
ions)
poisON11<-matrix(rpois(n=(nrow (ONcases)*1000), lambda=11),
                 ncol=nrow (ONcases))

# naming matrix columns and rows
colnames(poisON11) <- colnames(poisON11, do.NULL = FALSE,
                               prefix = "day.")
rownames(poisON11) <- rownames(poisON11, do.NULL = FALSE,
                               prefix = "iteration.")

# Estimated number of infections
# Multiply the daily new case count by each row of the matrix to gener
ate n
# simulations of the epi curve (estimated number of infections).
# Apply sweep operation that multiplies row by column multiplication
# (MARGIN=2) between poisON11 and ONcases$I
save.resON11<-(sweep(poisON11, MARGIN=2,ONcases$I, `*`))

# Calculate the median values for each day (by column)
EstInf_medianON11<-apply(save.resON11,2,median)

# Calculating 95% CrI
EstInf_lowerON11<- apply(save.resON11,2, quantile, probs=0.025)
EstInf_upperON11 <-apply(save.resON11,2, quantile, probs=0.975)
```

```
# create dataframe for the medians and CrI of Estimated number of infe
ction
EstInf_dfON11 <- data.frame(EstInf_medianON11,
                            EstInf_lowerON11,
                            EstInf_upperON11)

# preparing data for EpiEstim
newdataON11<-as.matrix.data.frame(save.resON11)

# Run EpiEstim::estimate_R on each of these simulated epi curves.
# Create a blank list
test_resON11<-vector(mode="list", length = nrow(newdataON11)) #list
# Save the EpiEstim estimate_R output object into the list
for(i in 1:nrow(newdataON11)){
  test_resON11 [[i]]<- estimate_R(newdataON11 [i,],
                             method="parametric_si",
                             config=config_lit)
}

  r_m1<-test_resON11


  # Generate 10 random values from the posterior R distribution
  # for each time step for each hypothetical time series
  sample_r_m1<-EpiEstim::sample_posterior_R(r_m1[[1]],
                                  n      = n,
                                  window = 1L)
  for(v in 2:length(r_m1)){
    sample_r_m1<-append(sample_r_m1,
                   sample_posterior_R(r_m1[[v]],
                                   n      = n,
                                   window = 1L))
  }
  sample_r_m1_df<-sample_r_m1
  for(window_number in 2:length(r_m1[[1]]$R$t_start)){
    sample_r_m1<-sample_posterior_R(r_m1[[1]],
                                  n      = n,
                                  window = window_number)
    for(v in 2:length(r_m1)){
      sample_r_m1<-append(sample_r_m1,
                     sample_posterior_R(r_m1[[v]],
                                     n      = n,
                                     window = window_number))
    }
    sample_r_m1_df<-rbind(sample_r_m1_df, sample_r_m1)
  }
```

```
  sample_r_m1_df<-as.data.frame(sample_r_m1_df)

  sample_r_m1_df<-cbind(Sample$dates[(r_m1[[1]]$R$t_end[1]):nrow(Sampl
e)],
                        sample_r_m1_df)

  colnames(sample_r_m1_df)[1]<-"dates"

  sample_r_m1_df <- sample_r_m1_df %>%
    rowwise() %>%
    mutate(Median=median(c_across(contains("V")))) %>%
    mutate(Lower=quantile(c_across(contains("V")), probs=0.025,
                          na.rm=F, names=F)) %>%
    mutate(Upper=quantile(c_across(contains("V")), probs=0.975,
                          na.rm=F, names=F)) %>%
    ungroup()  %>%
    dplyr::select(dates, Median, Lower, Upper, everything())

  ON11_sample_r_m1_df <-sample_r_m1_df

  M11_p1 <- ggplot(data    = ON11_sample_r_m1_df,
              mapping = aes(x = dates,
                            y = Median))+
    geom_line(col="black") +
    geom_ribbon(aes(ymin = Lower,
                    ymax = Upper),
                alpha = 0.2)

  M11_g1 <- M11_p1 + labs(y = expression(R[t]),
                 x = "Assumed date of infection") +
    geom_hline(yintercept = 1,
               linetype   = 2) +
    theme_classic() +
    theme_cowplot(font_size = 10)+
    scale_x_date(breaks = date_breaks("3 months"),
                 labels = date_format("%b %Y"),
                 limits = c(as.Date("2020-01-01"),
                            as.Date("2022-02-01")))
  M11_g1
  (M11_g1b<-M11_g1+scale_y_continuous(limits=c(0,8)))


  ### Export Figure S3
  tiff(file="ON_sensitivity figure_v4.tif",
       width=12, height=12,
```

```
      units='in', res=300)
  pdf(file="ON_sensitivity figure_v4.pdf", width=12, height=12)
  ggarrange (plot(d_ON),    # Rt based on case count
             plot(M4_g1b),  # Rt based on estimated infection count
                            # (Multiplier = 4)
             plot(M11_g1b), # Rt based on estimated infection count
                            # (Multiplier = 11)
             common.legend =  TRUE, legend = "bottom",
             ncol =1, nrow=3,align="v")

  dev.off()
```

ON Figure 1

```
# Create a case count data set
counts_on <- linelistON %>% count(Date.Infected) %>% drop_na(Date.Infe
cted)

rolling_counts_on <- counts_on %>%   # Generate new data set with a
                                     # 7-day sliding average daily cou
nts
  mutate(n = slider::slide_dbl(      # Create new columns using slide_
dbl()
    n,                               # Calculate new percentage change
    .f = ~mean(.x, na.rm = F),       # Function is mean
                                     # (with missing values NOT remove
d)
    .before = 6))                    # Each sliding window consists of
                                     # the row and 6 prior rows

###  7-day avg incidence
p_onI <- plot(epiestim_res_litON, "incid",
            options_I=list(xlab="Assumed date of infection"),
            legend = F)

d_onI <-p_onI+
  labs(title="Ontario", x="",y="Daily number of new cases")+
  geom_line(data=rolling_counts_on,aes(x=Date.Infected,y=n) )+
    #7-day rolling average line
  scale_x_date(breaks=date_breaks("3 months"),
               labels=date_format("%b %Y"),
               limits=c(as.Date("2019-11-29"),
                        as.Date("2022-01-27")))+
  theme_cowplot(font_size = 12)

###  incidence by variant
# Create ON variant data set
```

```r
VarsON <- read.csv("covid19-epiSummary-variants ontario.csv", header=T
)

# Format date reported YYYY-MM-DD
VarsON$dates<-as.Date(VarsON$dates, "%m/%d/%Y")

# Create date of infection (9 day shift)
VarsON$date_infected<-VarsON$dates-9

# Remove unnecessary variables
VarsON = subset(VarsON, select = -c(dates))

# Rename variables
VarsON <- VarsON %>%
  rename(date=date_infected)

# Create plot
d_onV1<-ggplot(VarsON, aes(date)) +theme_cowplot(font_size = 12)+
  labs(title="",x="", y="Daily number of new cases")+
  theme(legend.position="bottom")+
  scale_x_date(breaks=date_breaks("3 months") ,
               labels=date_format("%b %Y"),
               limits=c(as.Date("2019-11-29"),
                        as.Date("2022-01-27"))) +
  geom_line(aes(y = WT, color = "WT")) +
  geom_line(aes(y = Alpha, color = "Alpha")) +
  geom_line(aes(y = Delta, color = "Delta")) +
  geom_line(aes(y = Omicron, color = "Omicron")) +
  geom_line(aes(y = Other, color = "Other")) +
  scale_color_manual(name = "Variant",values = c("WT"     = "purple",
                                                 "Alpha"   = "red",
                                                 "Delta"   = "green",
                                                 "Omicron"= "blue",
                                                 "Other"   = "dimgrey")
)


###  Rt policy change and variant emergence
# Labels ABCDE are the sequence number for the starting date
# for each of your policies,
# and we have to start with the number 2 no matter what
# because the first Rt estimate is day 2 (with data from the previous
day)
# t_start(2,A,B,C,D,E)
t_start_ON <- c(2,61,85,239,340,361,455,535,578,682)
```

```r
# t_end is the day before the next policy implementation
t_end_ON <- c(60,84,238,339,360,454,534,577,681,739)

# Estimate R based on discrete policy windows instead of 7-day sliding
 window
ON_policy_res_parametric_si <- estimate_R(ONcases,
                                          method="parametric_si",
                                          config=make_config(list(
                                            t_start = t_start_ON,
                                            t_end = t_end_ON,
                                            mean_si=4.60,
                                            std_si=5.55)))
# Initial plot
p_onP <- plot(ON_policy_res_parametric_si, "R",
             options_I=list(xlab="Assumed date of infection"),
             legend = F)

# Creation of alpha-labels
a=data.frame(date=as.Date(c("2020-03-14",
                            "2020-04-07",
                            "2020-09-08",
                            "2020-12-18",
                            "2021-01-08",
                            "2021-04-12",
                            "2021-07-01",
                            "2021-08-13",
                            "2021-11-25"))
             ,y=c(0.5,0.6,0.45,0,0,0,0,0,0),
             event=c("A","B","C","D","E","F","G","H","I"))

# Create complete plot
g_onP <-p_onP+theme_classic()+theme_cowplot(font_size = 12)+
  theme(legend.position="none")+
  labs(title=" ",
       x="Assumed date of infection",
       y=expression('Policy Change R'[t]))+
  scale_x_date(breaks=date_breaks("3 months") , labels=date_format("%b
 %Y"),
               limits=c(as.Date("2019-12-01"), as.Date("2022-02-01")))
 +

  # horizontal line: reproduction number equals 1
  geom_hline(yintercept=1,
             color="red",
             linetype="dotted")+
```

```r
  #vertical line - nothing to change
  geom_vline(data=a, mapping=aes(xintercept=date),
             color="blue",
             linetype="dotted") +

  #labels - nothing to change
  annotate(geom = "label",x=as.Date(a$date),y=a$y,label=a$event,size=2
,
           fontface="bold",color="blue",check_overlap=TRUE)


### Export tiff file for Figure 1
tiff(file="ON_combined figure_v4.tif",
     width=12, height=12,
     units='in', res=300)
pdf(file="ON_combined figure_v4.pdf", width=12, height=12)
ggpubr::ggarrange (plot(d_onI), #incident+7-day rolling average
           plot(d_onV1), #variant
           plot(d_ON1),  #Rt
           plot(g_onP), #policy change Rt
           ncol =1, nrow=4, align="v")
dev.off()
```

ON Percent Change for Policy Change Rt

```r
set.seed(12345)
test_res_ON <- ON_policy_res_parametric_si

test_res_ON$R$t_start
(number_of_windows<-length(test_res_ON$R$t_start))
# Create empty lists to store the medians, 95% CrI, and 1000 random va
lues from
# the posterior R distribution
W_Median_list_ON          <-rep(NA,
                                number_of_windows) #vector
W_CrI_list_ON             <-vector(mode="list",
                                   length = number_of_windows) #list
W_R_list_ON               <-vector(mode="list",
                                   length = number_of_windows) #list
W_R_sample_change_list_ON  <-vector(mode="list",
                                   length = number_of_windows-1) #lis
t
W_R_sample_change_median_ON <-rep(NA,
                                 number_of_windows-1) #vector
W_R_sample_change_CrI_ON <-vector(mode="list",
                                 length = number_of_windows-1) #list
W_R_sample_percentage_change_list_ON<-vector(mode="list",
```

```
                                               length = number_of_windows
-1) #list
W_R_sample_percentage_change_median_ON <-rep(NA,
                                              number_of_windows-1) #vec
tor
W_R_sample_percentage_change_CrI_ON<-vector(mode="list",
                                               length = number_of_windows
-1) #list

# for loop
for(w in 1:number_of_windows){
  (W_Median_list_ON[w] <- test_res_ON$R$'Median(R)'[w])
  (W_CrI_list_ON[[w]]<- c(test_res_ON$R$'Quantile.0.025(R)'[w], test_r
es_ON$R$'Quantile.0.975(R)'[w]))
  #Generate 1000 random values from the posterior R distribution
  W_R_list_ON[[w]] <- sample_posterior_R(test_res_ON, n=1000, window=w
)
}


for(w in 1:(number_of_windows-1)){
  # Change as a fraction of the Rt during the previous window
  # Median and 95% credible intervals of the distribution of R_sample_
change
  # Convert to Percentage change
  # Multiply by 100 to get the percentage
  W_R_sample_change_list_ON[[w]]<-(W_R_list_ON[[w+1]]-W_R_list_ON[[w]]
)/W_R_list_ON[[w]]
  W_R_sample_change_median_ON[w]<-median(W_R_sample_change_list_ON[[w]
])
  W_R_sample_change_CrI_ON[[w]] <-c(quantile(W_R_sample_change_list_ON
[[w]],
                                                  probs=c(0.025, 0.975)))
  W_R_sample_percentage_change_list_ON[[w]]<-100*W_R_sample_change_lis
t_ON[[w]]
  W_R_sample_percentage_change_median_ON[w]<-100*W_R_sample_change_med
ian_ON[w]
  W_R_sample_percentage_change_CrI_ON[[w]] <-100*W_R_sample_change_CrI
_ON[[w]]
}
## Meaning: (R_next - R_previous) / R_previous
## R_next minus R_previous
## If this is positive, it means R_next > R_previous (i.e., increase)
## If this is negative, it means R_next < R_previous (i.e., decrease)
W_Median_list_ON
W_CrI_list_ON
```

```
W_R_sample_percentage_change_median_ON
W_R_sample_percentage_change_CrI_ON
```

British Columbia

BC Figure S4

```
# Create incidence
epi_day_BC <- incidence2::incidence(  # create incidence object
                                      # (incidence2 package)
  x = linelistBC,                     # dataset
  date_index = Date.Infected,         # date column
  interval = "week"                   # date grouping interval
)


### Weekly Incidence by Age Category
age_outbreak_BC <- incidence(
  linelistBC,                         # dataset
  date_index = Date.Infected,         # date column
  interval = "week",                  # Monday weekly aggregation of c
ases
  groups = Age,                       # age_cat is set as a group
  na_as_group = TRUE)                 # missing values assigned their
own group

# plot the grouped incidence object - age
(d_3_BC<-plot(
  age_outbreak_BC,                         # incidence object with age
_cat as group
  fill = Age,                              # age_cat is used for bar f
ill color
  centre_dates = FALSE,                    # Create date label
  date_format = "%b %Y") +                 # Format date label
  scale_fill_manual(values = cbpAge) +     # fill colors must be set b
eforehand
  labs(fill = "Age Category") +            # create label
  theme(legend.position = "bottom"))       # specify legend location


### Weekly Incidence by Sex
sex_outbreak_BC <- incidence(
  linelistBC,                         # dataset
  date_index = Date.Infected,         # date column
  interval = "week",                  # Monday weekly aggregation of c
ases
  groups = Sex,                       # age_cat is set as a group
```

```
  na_as_group = TRUE)                   # missing values assigned their
own group

# plot the grouped incidence object - sex
(d_5_BC<-plot(
  sex_outbreak_BC,                        # incidence object with age_ca
t as group
  fill = Sex,                                # age_cat is used for bar f
ill color
  centre_dates = FALSE,                      # Create date label
  date_format = "%b %Y") +                   # Format date label
  scale_fill_manual(values = cbpSex) +       # fill colors must be set b
eforehand
  labs(fill = "Sex Category") +           # create label
  theme(legend.position = "bottom"))         # specify legend location


### Weekly Incidence by PHRAs
location_outbreak_BC <- incidence(
  linelistBC,                           # dataset
  date_index = Date.Infected,           # date column
  interval = "week",                    # Monday weekly aggregation of c
ases
  groups = PH.Unit,                     # age_cat is set as a group
  na_as_group = TRUE)                   # missing values assigned their
own group

# rename PH.Unit - add RA
location_outbreak_BC$PH.Unit <- factor(location_outbreak_BC$PH.Unit,
                                       levels = c("Fraser HA",
                                                  "Interior HA",
                                                  "Northern HA",
                                                  "Vancouver Coastal H
A",
                                                  "Vancouver Island HA
",
                                                  "Unknown"))
# plot the grouped incidence object - PHRAs
(d_7_BC<- plot(
  location_outbreak_BC,                      # incidence object with age
_cat as group
  fill = PH.Unit,                            # age_cat is used for bar f
ill color
  centre_dates = FALSE,                      # Create date label
  date_format = "%b %Y") +                   # Format date label
  scale_fill_manual(values = cbpPHU1) +      # fill colors must be set b
```

```
eforehand
  labs(fill = "RHA") +                        # create label
  theme(legend.position = "bottom"))          # specify legend location


### Export 3 panel figure S4
prefix<-"BC_EpiCurve_Age_Sex_PHU"       # name for figure
tiffname<-paste0(prefix, "_3panel.tif")   # paste file type to name
tiff(tiffname,
     height=8, width=10,
     units="in",res=300)        # specify figure aspects
pdf(file="BC_EpiCurve_Age_Sex_PHU.pdf", width=10, height=8)
gridExtra::grid.arrange(d_3_BC,
                        d_5_BC,
                        d_7_BC,
                        nrow=3) # specify figure arrangement
dev.off()
```

BC Figure S5

Monthly log10 incidence by PHRAs

```
mapsBC <- linelistBC[c("Province", "PH.Unit", "Date.Infected")]
mapsBC$X <- 1

# Unique ID creation M2001 = January 2020 | M2105 = May 2021
mapsBC$mo <- paste0("M", format(as.Date(mapsBC$Date.Infected), "%y%m")
)

# Calculate the cumulative case count by PH.Unit
mapsBC<-mapsBC %>%
  group_by(Province, PH.Unit) %>%
  arrange(Province, PH.Unit, Date.Infected)  %>%
  mutate(cumcase_RHA = cumsum(X))

# Collapse line list data to monthly data
mapsBC <- mapsBC %>%
  group_by(Province, PH.Unit, mo) %>%
  filter(cumcase_RHA == max(cumcase_RHA)) %>%
  arrange(Province, PH.Unit, mo)

# Delete unnecessary variables
mapsBC = subset(mapsBC, select = -c(X, Date.Infected))

# Transform data from long to wide format
mapsBC<-spread(mapsBC, mo, cumcase_RHA)
```

```r
# Merge geometry polygons based on PH_Unit
# Source: https://www.jla-data.net/eng/merging-geometry-of-sf-objects-
in-r/
BC_map <- mapname %>%
  group_by(PH_Unit) %>%
  summarise()

# Identify ON map regions
BC_PHRU <- c("Fraser HA",
             "Interior HA",
             "Northern HA",
             "Vancouver Coastal HA",
             "Vancouver Island HA")

# Select map regions in ON only
BC_map<-BC_map %>%
  filter(PH_Unit %in% (BC_PHRU))

ggplot()+geom_sf(data=BC_map, aes(fill=PH_Unit)) #Verify Maps of the 5
 PH_unit

BC_map1 <- merge(BC_map, mapsBC, by.x = 'PH_Unit', by.y = 'PH.Unit', a
ll.x=T)

### To prepare for a loop
(list1BC<-colnames(mapsBC[,c(4:27)]))
(list2BC<-stringr::str_sub(list1BC, start=2L,end=3L))
(list3BC<-stringr::str_sub(list1BC, start=4L,end=5L))
(list2aBC<-paste("20", list2BC, sep=""))
(list3aBC<-as.integer(list3BC))

month.name[list3aBC]
month.abb[list3aBC]
paste(month.abb[list3aBC], list2aBC, sep=" ")
(title_vectorBC <- paste(month.abb[list3aBC], list2aBC, sep=" "))

### Testing loop using list1BC and title_vector:
ggplot()+
  geom_sf(data=BC_map1, aes(fill=log10(.data[[list1BC[1]]]))) +
  theme_bw() +
  coord_sf(default_crs = sf::st_crs(4326)) +
  myscale +
  mytheme +
  ggtitle(title_vectorBC[1])

# Write a function
```

```r
BC_individual_plot <- function(index,
                               data,
                               list1=list1BC,
                               title_vector=title_vectorBC,
                               myscale=myscale,
                               mytheme=mytheme){
  ggplot()+
    geom_sf(data=data, aes(fill=log10(.data[[list1BC[index]]]))) +
    theme_bw() +
    coord_sf(default_crs = sf::st_crs(4326)) +
    myscale +
    mytheme +
    ggtitle(title_vectorBC[index])
}

length(title_vectorBC)
(indicesBC<-c(1:24))

#Create empty list of length same as the length of indices
BC_plots_list<-vector(mode='list', length=length(indicesBC))

# testing
BC_individual_plot(1, BC_map1, list1BC, title_vectorBC, myscale, mythe
me)
BC_individual_plot(4, BC_map1, list1BC, title_vectorBC, myscale, mythe
me)

### A for-loop using the ON_individual_plot function
### to create the individual plots
for (i in indicesBC){
  BC_plots_list[[i]]<-BC_individual_plot(i,
                                         BC_map1,
                                         list1BC,
                                         title_vectorBC,
                                         myscale,
                                         mytheme)
}

### Export Figure S5
legend_bc <- get_legend(BC_plots_list[[length(title_vectorBC)]]+
                          theme(legend.position="right")+
                          labs(fill = "log10 CC"))
prefix<-"BC_MonMap"
tiffname<-paste0(prefix, ".tif")
tiff(tiffname,height=8, width=8, units='in', res=300)
pdf(file="BC_MonMap.pdf", width=8, height=8)
```

```
gridExtra::grid.arrange(BC_plots_list[[2]],
                        BC_plots_list[[3]],BC_plots_list[[4]],
                        BC_plots_list[[5]],BC_plots_list[[6]],
                        BC_plots_list[[7]],BC_plots_list[[8]],
                        BC_plots_list[[9]],BC_plots_list[[10]],
                        BC_plots_list[[11]],BC_plots_list[[12]],
                        BC_plots_list[[13]],BC_plots_list[[14]],
                        BC_plots_list[[15]],BC_plots_list[[16]],
                        BC_plots_list[[17]],BC_plots_list[[18]],
                        BC_plots_list[[19]],BC_plots_list[[20]],
                        BC_plots_list[[21]],BC_plots_list[[22]],
                        BC_plots_list[[23]],BC_plots_list[[24]],
                        legend_bc, ncol=6, nrow=4)
dev.off()
```

BC Figure S6

3-panel sensitivity

```
# Create dataset of case counts by day
BCcases <- incidence2::incidence(linelistBC,
                                 date_index = Date.Infected) %>%
  tidyr::complete(date_index = seq.Date(    # ensure all dates are rep
resented
    from = min(date_index, na.rm = T),
    to = max(date_index, na.rm=T),
    by = "day"),
    fill = list(count = 0)) %>%             # convert NA counts to 0
  rename(I = count,         # rename to names expected by EpiEstim::es
timate_R
         dates = date_index)

# Set mean and stand deviation for case counts
config_lit <- make_config(
  mean_si = 4.6,
  std_si = 5.55
)

# Estimate R for - Epi curves, R, and SI distribution
epiestim_res_litBC <- estimate_R(
  incid = BCcases,
  method = "parametric_si",
  config = config_lit
)

### median Rt
dfBC<-data.frame(epiestim_res_litBC$dates[8:length(epiestim_res_litBC$
```

```
dates)],
                  epiestim_res_litBC$R$`Median(R)`,
                  epiestim_res_litBC$R$`Quantile.0.025(R)`,
                  epiestim_res_litBC$R$`Quantile.0.975(R)`)
colnames(dfBC)<-c("dates", "median", "lower", "upper")
w_BC <-ggplot(data=dfBC) +
  geom_line(aes(x=dates, y=median))+
  geom_ribbon(aes(x=dates, ymin=lower,
                  ymax=upper), fill="grey70", alpha=0.5)

d_BC <-w_BC+theme_classic()+
  labs(title="British Columbia",x=" ",y=expression(R[t]))+
  theme(legend.position="bottom")+
  scale_x_date(breaks=date_breaks("3 months") ,
               labels=date_format("%b %Y"),
               limits=c(as.Date("2020-01-01"), as.Date("2022-02-01")))
+
  scale_y_continuous(limits = c(0,8))+
  geom_hline(yintercept=1.0, color="red",linetype="dotted") +
  theme_cowplot(font_size = 10)+
  theme(legend.position = "bottom")

# Removing Y-axis limit for manuscript figure 2
d_BC1 <-w_BC+theme_cowplot(font_size = 12)+
  labs(title="",x=" ",y=expression(R[t]))+
  theme(legend.position="bottom")+
  scale_x_date(breaks=date_breaks("3 months") , labels=date_format("%b
 %Y"),
               limits=c(as.Date("2019-11-29"), as.Date("2022-01-27")))
+
  scale_y_continuous(limits = c(0,8))+
  geom_hline(yintercept=1.0, color="red",linetype="dotted") +
  theme(legend.position = "bottom")

### Multiplier: Poisson distribution with a mean of 4
set.seed(1234)
# Generating a matrix of random numbers: each column (x-axis) is a day
, and
# each row (y-axis) is an iteration (e.g., you may have n=1000 simulat
ions)
poisBC<-matrix(rpois(n= (nrow (BCcases)*1000), lambda=4),
               ncol=nrow (BCcases))

# Renaming matrix columns and rows
colnames(poisBC) <- colnames(poisBC, do.NULL = FALSE, prefix = "day.")
rownames(poisBC) <- rownames(poisBC, do.NULL = FALSE, prefix = "iterat
```

```
ion.")

# Estimated number of infections
# Multiply the daily new case count by each row of the matrix to gener
ate n
# simulations of the epi curve (estimated number of infections).
# Apply sweep operation that multiplies row by column multiplication
# (MARGIN=2) between poisBC and BCcases$I
save.resBC<-(sweep(poisBC, MARGIN=2,BCcases$I, `*`))

# Calculate the median values for each day (by column)
EstInf_too1BC<-apply(save.resBC,2,median)

# Calculating 95% CrI
EstInf_lowerBC <- apply(save.resBC,2, quantile, probs=0.025)
EstInf_upperBC <- apply(save.resBC,2, quantile, probs=0.975)

# Create dataframe to for the medians and CrI
EstInf_dfBC <- data.frame(EstInf_too1BC, EstInf_lowerBC, EstInf_upperB
C)

# Preparing data for EpiEstim
newdataBC<-as.matrix.data.frame(save.resBC)

# Run your EpiEstim on each of these simulated epi curves.
test_resBC<-vector(mode="list", length = nrow(newdataBC)) # list

for(i in 1:nrow(newdataBC)){
  test_resBC [[i]]<- estimate_R(newdataBC [i,],
                                method="parametric_si",
                                config=config_lit)
}

r_m2<-test_resBC
Sample2<-BCcases

# Generate 10 random values from the posterior R distribution
# for each time step for each hypothetical time series
sample_r_m2<-EpiEstim::sample_posterior_R(r_m2[[1]],
                                          n      = n,
                                          window = 1L)
for(v in 2:length(r_m2)){
  sample_r_m2<-append(sample_r_m2,
                      sample_posterior_R(r_m2[[v]],
                                         n      = n,
                                         window = 1L))
```

```
}
sample_r_m2_df<-sample_r_m2
for(window_number in 2:length(r_m2[[1]]$R$t_start)){
  sample_r_m2<-sample_posterior_R(r_m2[[1]],
                                n      = n,
                                window = window_number)
  for(v in 2:length(r_m2)){
    sample_r_m2<-append(sample_r_m2,
                        sample_posterior_R(r_m2[[v]],
                                           n      = n,
                                           window = window_number))
  }
  sample_r_m2_df<-rbind(sample_r_m2_df, sample_r_m2)
}

sample_r_m2_df<-as.data.frame(sample_r_m2_df)

sample_r_m2_df<-cbind(Sample2$dates[(r_m2[[1]]$R$t_end[1]):nrow(Sample
2)],
                      sample_r_m2_df)

colnames(sample_r_m2_df)[1]<-"dates"


sample_r_m2_df <- sample_r_m2_df %>%
  rowwise() %>%
  mutate(Median=median(c_across(contains("V")))) %>%
  mutate(Lower=quantile(c_across(contains("V")), probs=0.025,
                        na.rm=F, names=F)) %>%
  mutate(Upper=quantile(c_across(contains("V")), probs=0.975,
                        na.rm=F, names=F)) %>%
  ungroup()  %>%
  dplyr::select(dates, Median, Lower, Upper, everything())

BC4_sample_r_m2_df <- sample_r_m2_df

M4_p2 <- ggplot(data    = BC4_sample_r_m2_df,
                mapping = aes(x = dates,
                              y = Median))+
  geom_line(col="black") +
  geom_ribbon(aes(ymin = Lower,
                  ymax = Upper),
              alpha = 0.2)

M4_g2 <- M4_p2 + labs(y = expression(R[t]),
                      x = "") +
```

```r
  geom_hline(yintercept = 1,
             linetype   = 2) +
  theme_classic() +
  theme_cowplot(font_size = 10)+
  scale_x_date(breaks = date_breaks("3 months"),
               labels = date_format("%b %Y"),
               limits = c(as.Date("2020-01-01"),
                          as.Date("2022-02-01")))
M4_g2
(M4_g2b<-M4_g2+scale_y_continuous(limits=c(0,8)))

### Multiplier: Poisson distribution with a mean of 11
# Generating a matrix of random numbers: each column (x-axis) is a day
, and
# each row (y-axis) is an iteration (e.g., you may have n=1000 simulat
ions)
poisBC11<-matrix(rpois(n= (nrow (BCcases)*1000), lambda=11),
                 ncol=nrow (BCcases))

# naming matrix columns and rows
colnames(poisBC11) <- colnames(poisBC11, do.NULL = FALSE,
                              prefix = "day.")
rownames(poisBC11) <- rownames(poisBC11, do.NULL = FALSE,
                              prefix = "iteration.")

# Estimated number of infections
# Multiply the daily new case count by each row of the matrix to gener
ate n
# simulations of the epi curve (estimated number of infections).
# Apply sweep operation that multiplies row by column multiplication
# (MARGIN=2) between poisBC11 and BCcases$I
save.resBC11<-(sweep(poisBC11, MARGIN=2,BCcases$I, `*`))

# calculate the median values for each day (by column)
EstInf_medianBC11<-apply(save.resBC11,2, median)

# Calculating 95% CrI
EstInf_lowerBC11<- apply(save.resBC11,2, quantile, probs=0.025)
EstInf_upperBC11 <-apply(save.resBC11,2, quantile, probs=0.975)

# create dataframe for the medians and CrI of Estimated number of infe
ction
EstInf_dfBC11 <- data.frame(EstInf_medianBC11,
                            EstInf_lowerBC11,
                            EstInf_upperBC11)
```

```r
# preparing data for EpiEstim
newdataBC11<-as.matrix.data.frame(save.resBC11)

# Run your EpiEstim on each of these simulated epi curves.
# Create a blank list
test_resBC11<-vector(mode="list", length = nrow(newdataBC11)) #list
# Save the EpiEstim estimate_R output object into the list
for(i in 1:nrow(newdataBC11)){
  test_resBC11 [[i]]<- estimate_R(newdataBC11 [i,],
                                  method="parametric_si",
                                  config=config_lit)
}

r_m3<-test_resBC11


# Generate 10 random values from the posterior R distribution
# for each time step for each hypothetical time series
sample_r_m3<-EpiEstim::sample_posterior_R(r_m3[[1]],
                                          n      = n,
                                          window = 1L)
for(v in 2:length(r_m3)){
  sample_r_m3<-append(sample_r_m3,
                      sample_posterior_R(r_m3[[v]],
                                         n      = n,
                                         window = 1L))
}
sample_r_m3_df<-sample_r_m3
for(window_number in 2:length(r_m3[[1]]$R$t_start)){
  sample_r_m3<-sample_posterior_R(r_m3[[1]],
                                  n      = n,
                                  window = window_number)
  for(v in 2:length(r_m3)){
    sample_r_m3<-append(sample_r_m3,
                        sample_posterior_R(r_m3[[v]],
                                           n      = n,
                                           window = window_number))
  }
  sample_r_m3_df<-rbind(sample_r_m3_df, sample_r_m3)
}

sample_r_m3_df<-as.data.frame(sample_r_m3_df)

sample_r_m3_df<-cbind(Sample2$dates[(r_m3[[1]]$R$t_end[1]):nrow(Sample
2)],
                      sample_r_m3_df)
```

```
colnames(sample_r_m3_df)[1]<-"dates"


sample_r_m3_df <- sample_r_m3_df %>%
  rowwise() %>%
  mutate(Median=median(c_across(contains("V")))) %>%
  mutate(Lower=quantile(c_across(contains("V")), probs=0.025,
                        na.rm=F, names=F)) %>%
  mutate(Upper=quantile(c_across(contains("V")), probs=0.975,
                        na.rm=F, names=F)) %>%
  ungroup()  %>%
  dplyr::select(dates, Median, Lower, Upper, everything())

BC11_sample_r_m3_df <-sample_r_m3_df

M11_p2 <- ggplot(data    = BC11_sample_r_m3_df,
             mapping = aes(x = dates,
                           y = Median))+
  geom_line(col="black") +
  geom_ribbon(aes(ymin = Lower,
                  ymax = Upper),
              alpha = 0.2)

M11_g2 <- M11_p2 + labs(y = expression(R[t]),
                x = "Assumed date of infection") +
  geom_hline(yintercept = 1,
             linetype   = 2) +
  theme_classic() +
  theme_cowplot(font_size = 10)+
  scale_x_date(breaks = date_breaks("3 months"),
               labels = date_format("%b %Y"),
               limits = c(as.Date("2020-01-01"),
                          as.Date("2022-02-01")))
M11_g2
(M11_g2b<-M11_g2+scale_y_continuous(limits=c(0,8)))


### Export Figure S6
tiff(file="BC_sensitivity figure_v4.tif",
     width=12, height=12,
     units='in', res=300)
pdf(file="BC_sensitivity figure_v4.pdf", width = 12, height = 12)
ggarrange (plot(d_BC), #Rt based on case count
           plot(M4_g2b), # Rt based on estimated infection count
                         # (Multiplier = 4)
```

```
            plot(M11_g2b), # Rt based on estimated infection count
                           # (Multiplier = 11)
            common.legend =  TRUE, legend = "bottom",
            ncol =1, nrow=3,align="v")

dev.off()
```

BC Figure 2

```
# Create a case count data set
counts_bc <- linelistBC %>% count(Date.Infected) %>% drop_na(Date.Infe
cted)

rolling_counts_bc <- counts_bc %>%   # Generate new data set with
                                     # a 7-day sliding average daily c
ounts
  mutate(n = slider::slide_dbl(      # Create new columns using slide_
dbl()
    n,                               # Calculate new percentage change
    .f = ~mean(.x, na.rm = F),       # Function is mean
                                     # (with missing values NOT remove
d)
    .before = 6))                    # Each sliding window is the row
                                     # and 6 prior rows

###  7-day avg incidence
p_bcI <- plot(epiestim_res_litBC, "incid",
              options_I=list(xlab="Assumed date of infection"),
              legend = F)

d_bcI <-p_bcI+
  labs(title="British Columbia", x="",y="Daily number of new cases")+
  geom_line(data=rolling_counts_bc,aes(x=Date.Infected,y=n) )+
             # 7-day rolling average line
  scale_x_date(breaks=date_breaks("3 months") ,
               labels=date_format("%b %Y"),
               limits=c(as.Date("2019-11-29"),
                        as.Date("2022-01-27")))+
  theme_cowplot(font_size = 12)

###  incidence by variant
# Create ON variant data set
VarsBC <- read.csv("BC Variants.csv", header=T)

# Format date reported YYYY-MM-DD
VarsBC$dates<-as.Date(VarsBC$dates, "%m/%d/%Y")
```

```r
# Create date of infection (9 day shift)
VarsBC$date_infected<-VarsBC$dates-9

# Remove unnecessary variables
VarsBC = subset(VarsBC, select = -c(dates))

# Rename variables
VarsBC <- VarsBC %>%
  rename(date=date_infected)

# Create plot
d_bcV1<-ggplot(VarsBC, aes(date)) +
  theme_cowplot(font_size = 12)+
  labs(title="",x="", y="Daily number of new cases")+
  theme(legend.position="bottom")+
  scale_x_date(breaks=date_breaks("3 months"),
               labels=date_format("%b %Y"),
               limits=c(as.Date("2019-11-29"),
                        as.Date("2022-01-27"))) +
  geom_line(aes(y = WT, color = "WT")) +
  geom_line(aes(y = Alpha, color = "Alpha")) +
  geom_line(aes(y = Delta, color = "Delta")) +
  geom_line(aes(y = Omicron, color = "Omicron")) +
  geom_line(aes(y = Other, color = "Other")) +
  scale_color_manual(name = "Variant",values = c("WT" = "purple",
                                                 "Alpha" = "red",
                                                 "Delta"="green",
                                                 "Omicron"="blue",
                                                 "Other"="dimgrey"))


###  Rt policy change and variant emergence
t_start_BC <- c(2,55,79,233,334,355,449,529,572,672)

# t_end is the day before the next policy implementation
t_end_BC <- c(54,78,232,333,354,448,528, 571,671,730)

# Estimate R based on discrete policy windows instead of 7-day sliding
 window
BC_policy_res_parametric_si <- estimate_R(BCcases,
                                          method="parametric_si",
                                          config=make_config(list(
                                            t_start = t_start_BC,
                                            t_end = t_end_BC,
                                            mean_si=4.60,
                                            std_si=5.55)))
```

```r
# Initial plot
p_bcP <- plot(BC_policy_res_parametric_si, "R",
              options_I=list(xlab="Assumed date of infection"),
              legend = F)

# Creation of alpha-labels
b=data.frame(date=as.Date(c("2020-03-14",
                            "2020-04-07",
                            "2020-09-08",
                            "2020-12-18",
                            "2021-01-08",
                            "2021-04-12",
                            "2021-07-01",
                            "2021-08-13",
                            "2021-11-21")),
             y=c(0.5,0.6,0.45,0,0,0,0,0,0),
             event=c("A","B","C","D","E","F","G","H","I"))

# Create complete plot
g_bcP <-p_bcP+theme_classic()+
  theme_cowplot(font_size = 12)+
  theme(legend.position="none")+
  labs(title=" ",
       x="Assumed date of infection",
       y=expression('Policy Change R'[t]))+
  scale_x_date(breaks=date_breaks("3 months") ,
               labels=date_format("%b %Y"),
               limits=c(as.Date("2019-12-01"),
                        as.Date("2022-02-01"))) +

  geom_hline(yintercept=1, color="red",linetype="dotted")+
  geom_vline(data=b, mapping=aes(xintercept=date), color="blue", linet
ype="dotted") +

  #labels - nothing to change
  annotate(geom = "label",x=as.Date(b$date),y=b$y,label=b$event,size=2
,
           fontface="bold",color="blue",check_overlap=TRUE)


### Export tiff file for Figure 2
tiff(file="BC_combined figure_v4.tif", width=12, height=12, units='in'
, res=300)
pdf(file="BC_combined figure_v4.pdf", width=12, height=12)
ggpubr::ggarrange (plot(d_bcI), #incident+7-day rolling average
                   plot(d_bcV1), #variant
```

```
                    plot(d_BC1), #Rt
                    plot(g_bcP), #policy change Rt
                    ncol =1, nrow=4, align="v")
dev.off()
```

BC Percent Change for Policy Change Rt

```
set.seed(12345)
test_res_BC <- BC_policy_res_parametric_si

test_res_BC$R$t_start
(number_of_windows<-length(test_res_BC$R$t_start))
# Create empty lists to store the medians, 95% CrI, and 1000 random va
lues from
# the posterior R distribution
W_Median_list_BC           <-rep(NA,
                                 number_of_windows) #vector
W_CrI_list_BC              <-vector(mode="list",
                                    length = number_of_windows) #list
W_R_list_BC                <-vector(mode="list",
                                    length = number_of_windows) #list
W_R_sample_change_list_BC  <-vector(mode="list",
                                    length = number_of_windows-1) #lis
t
W_R_sample_change_median_BC <-rep(NA,
                                  number_of_windows-1) #vector
W_R_sample_change_CrI_BC <-vector(mode="list",
                                  length = number_of_windows-1) #list
W_R_sample_percentage_change_list_BC <-vector(mode="list",
                                              length = number_of_windo
ws-1)#list
W_R_sample_percentage_change_median_BC <-rep(NA,
                                             number_of_windows-1) #vec
tor
W_R_sample_percentage_change_CrI_BC <-vector(mode="list",
                                             length = number_of_window
s-1) #list

# for loop
for(w in 1:number_of_windows){
  (W_Median_list_BC[w] <- test_res_BC$R$'Median(R)'[w])
  (W_CrI_list_BC[[w]]<- c(test_res_BC$R$'Quantile.0.025(R)'[w], test_r
es_BC$R$'Quantile.0.975(R)'[w]))
  #Generate 1000 random values from the posterior R distribution
  W_R_list_BC[[w]] <- sample_posterior_R(test_res_BC, n=1000, window=w
)
}
```

```
for(w in 1:(number_of_windows-1)){
  # Change as a fraction of the Rt during the previous window
  # Median and 95% credible intervals of the distribution of R_sample_
change
  # Convert to Percentage change
  # Multiply by 100 to get the percentage
  W_R_sample_change_list_BC[[w]]<-(W_R_list_BC[[w+1]]-W_R_list_BC[[w]]
)/W_R_list_BC[[w]]
  W_R_sample_change_median_BC[w]<-median(W_R_sample_change_list_BC[[w]
])
  W_R_sample_change_CrI_BC[[w]] <-c(quantile(W_R_sample_change_list_BC
[[w]],
                                              probs=c(0.025, 0.975)))
  W_R_sample_percentage_change_list_BC[[w]]<-100*W_R_sample_change_lis
t_BC[[w]]
  W_R_sample_percentage_change_median_BC[w]<-100*W_R_sample_change_med
ian_BC[w]
  W_R_sample_percentage_change_CrI_BC[[w]] <-100*W_R_sample_change_CrI
_BC[[w]]
}
## Meaning: (R_next - R_previous) / R_previous
## R_next minus R_previous
## If this is positive, it means R_next > R_previous (i.e., increase)
## If this is negative, it means RW_Median_list_BC_next < R_previous (
i.e., decrease)
W_Median_list_BC
W_CrI_list_BC
W_R_sample_percentage_change_median_BC
W_R_sample_percentage_change_CrI_BC
```

Alberta

AB Figure S7

```
# Create incidence
epi_day_AB <- incidence2::incidence(       # create incidence object (
incidence2 package)
  x = linelistAB,                          # dataset
  date_index = Date.Infected,              # date column
  interval = "week"                        # date grouping interval
)


### Weekly Incidence by Age Category
age_outbreak_AB <- incidence(
```

```
  linelistAB,                              # dataset
  date_index = Date.Infected,              # date column
  interval = "week",                       # Monday weekly aggregation of c
ases
  groups = Age,                            # age_cat is set as a group
  na_as_group = TRUE)                      # missing values assigned their
own group

# plot the grouped incidence object - age
(d_3_AB<-plot(
  age_outbreak_AB,                              # incidence object with age
_cat as group
  fill = Age,                                   # age_cat is used for bar f
ill color
  centre_dates = FALSE,                         # Create date label
  date_format = "%b %Y") +                      # Format date label
  scale_fill_manual(values = cbpAge) +          # fill colors must be set b
eforehand
  labs(fill = "Age Category") +                 # create label
  theme(legend.position = "bottom"))            # specify legend location


### Weekly Incidence by Sex
sex_outbreak_AB <- incidence(
  linelistAB,                              # dataset
  date_index = Date.Infected,              # date column
  interval = "week",                       # Monday weekly aggregation of c
ases
  groups = Sex,                            # age_cat is set as a group
  na_as_group = TRUE)                      # missing values assigned their
own group

# plot the grouped incidence object - sex
(d_5_AB<-plot(
  sex_outbreak_AB,                            # incidence object with age_ca
t as group
  fill = Sex,                                   # age_cat is used for bar f
ill color
  centre_dates = FALSE,                         # Create date label
  date_format = "%b %Y") +                      # Format date label
  scale_fill_manual(values = cbpSex) +          # fill colors must be set b
eforehand
  labs(fill = "Sex Category") +              # create label
  theme(legend.position = "bottom"))            # specify legend location
```

```
### Weekly Incidence by PHRAs
location_outbreak_AB <- incidence(
  linelistAB,                        # dataset
  date_index = Date.Infected,        # date column
  interval = "week",                 # Monday weekly aggregation of c
ases
  groups = PH.Unit,                  # age_cat is set as a group
  na_as_group = TRUE)                # missing values assigned their
own group

# rename PH.Unit - add RA
location_outbreak_AB$PH.Unit <- factor(location_outbreak_AB$PH.Unit,
                                       levels = c("Calgary HSZ",
                                                  "Central HSZ",
                                                  "Edmonton HSZ",
                                                  "North HSZ",
                                                  "South HSZ",
                                                  "Unknown"))
# plot the grouped incidence object - PHRAs
(d_7_AB<- plot(
  location_outbreak_AB,                   # incidence object with age
_cat as group
  fill = PH.Unit,                         # age_cat is used for bar f
ill color
  centre_dates = FALSE,                   # Create date label
  date_format = "%b %Y") +                # Format date label
  scale_fill_manual(values = cbpPHU1) +   # fill colors must be set b
eforehand
  labs(fill = "HSZ") +                    # create label
  theme(legend.position = "bottom"))      # specify legend location


### Export 3 panel figure S7
prefix<-"AB_EpiCurve_Age_Sex_PHU"        # name for figure
tiffname<-paste0(prefix, "_3panel.tif")     # paste file type to name
tiff(tiffname,
     height=8, width=10,
     units="in",res=300)                    # specify figure aspects
pdf(file="AB_EpiCurve_Age_Sex_PHU.pdf", width=10, height=8)
gridExtra::grid.arrange(d_3_AB, d_5_AB, d_7_AB,
                        nrow=3)             # specify figure arrangeme
nt
dev.off()
```

AB Figure S8

Monthly log10 incidence by PHRAs

```
mapsAB <- linelistAB[c("Province", "PH.Unit", "Date.Infected")]
mapsAB$X <- 1

# Unique ID creation M2001 = January 2020 | M2105 = May 2021
mapsAB$mo <- paste0("M", format(as.Date(mapsAB$Date.Infected), "%y%m")
)

# Calculate the cumulative case count by PH.Unit
mapsAB<-mapsAB %>%
  group_by(Province, PH.Unit) %>%
  arrange(Province, PH.Unit, Date.Infected)  %>%
  mutate(cumcase_HSZ = cumsum(X))

# Collapse line list data to monthly data
mapsAB <- mapsAB %>%
  group_by(Province, PH.Unit, mo) %>%
  filter(cumcase_HSZ == max(cumcase_HSZ)) %>%
  arrange(Province, PH.Unit, mo)

# Delete unnecessary variables
mapsAB = subset(mapsAB, select = -c(X, Date.Infected))

# Transform data from long to wide format
mapsAB<-spread(mapsAB, mo, cumcase_HSZ)

#Remove Unknown and NA from PH.Unit
mapsAB<-subset(mapsAB, PH.Unit=="Calgary HSZ" | PH.Unit=="Central HSZ"
 |
                 PH.Unit=="Edmonton HSZ" | PH.Unit=="North HSZ" |
                 PH.Unit=="South HSZ")

# Create AB Map
AB_map <- mapname %>%
  group_by(PH_Unit) %>%
  summarise()

# Identify ON map regions
AB_PHRU <- c("Calgary HSZ", "Central HSZ", "Edmonton HSZ",
             "North HSZ", "South HSZ")

# Select map regions in ON only
AB_map<-AB_map %>%
  filter(PH_Unit %in% (AB_PHRU))
```

```r
ggplot()+
  geom_sf(data=AB_map, aes(fill=PH_Unit)) #Review Maps of the 5 PH_uni
t

AB_map1 <- merge(AB_map, mapsAB, by.x = 'PH_Unit', by.y = 'PH.Unit', a
ll.x=T)

### To prepare for a loop
(list1AB<-colnames(mapsAB[,c(4:26)]))
(list2AB<-stringr::str_sub(list1AB, start=2L,end=3L))
(list3AB<-stringr::str_sub(list1AB, start=4L,end=5L))
(list2aAB<-paste("20", list2AB, sep=""))
(list3aAB<-as.integer(list3AB))

month.name[list3aAB]
month.abb[list3aAB]
paste(month.abb[list3aAB], list2aAB, sep=" ")
(title_vectorAB <- paste(month.abb[list3aAB], list2aAB, sep=" "))

# Write a function
AB_individual_plot <- function(index,
                               data,
                               list1=list1AB,
                               title_vector=title_vectorAB,
                               myscale=myscale,
                               mytheme=mytheme){
  ggplot()+
    geom_sf(data=data, aes(fill=log10(.data[[list1AB[index]]]))) +
    theme_bw() +
    coord_sf(default_crs = sf::st_crs(4326)) +
    myscale +
    mytheme +
    ggtitle(title_vectorAB[index])
}

length(title_vectorAB)
#[1] 23
(indicesAB<-c(1:23))

#Create empty list of length same as the length of indices
AB_plots_list<-vector(mode='list', length=length(indicesAB))

# testing
AB_individual_plot(21, AB_map1, list1AB, title_vectorAB, myscale, myth
eme)
```

```
AB_individual_plot(22, AB_map1, list1AB, title_vectorAB, myscale, myth
eme)

### A for-loop using the ON_individual_plot function
### to create the individual plots
for (i in indicesAB){
  AB_plots_list[[i]]<-AB_individual_plot(i,
                                          AB_map1,
                                          list1AB,
                                          title_vectorAB,
                                          myscale,
                                          mytheme)
}

### Export Figure S8
legend_ab <- get_legend(AB_plots_list[[length(title_vectorAB)]]+
                          theme(legend.position="right")+
                          labs(fill="log10 CC"))

prefix<-"AB_MonMap"
tiffname<-paste0(prefix, ".tif")
tiff(tiffname,height=8, width=8, units='in', res=300)
pdf(file="AB_MonMap.pdf", width=8, height=8)
gridExtra::grid.arrange(AB_plots_list[[1]],AB_plots_list[[2]],
                        AB_plots_list[[3]],AB_plots_list[[4]],
                        AB_plots_list[[5]],AB_plots_list[[6]],
                        AB_plots_list[[7]],AB_plots_list[[8]],
                        AB_plots_list[[9]],AB_plots_list[[10]],
                        AB_plots_list[[11]],AB_plots_list[[12]],
                        AB_plots_list[[13]],AB_plots_list[[14]],
                        AB_plots_list[[15]],AB_plots_list[[16]],
                        AB_plots_list[[17]],AB_plots_list[[18]],
                        AB_plots_list[[19]],AB_plots_list[[20]],
                        AB_plots_list[[21]],AB_plots_list[[22]],
                        AB_plots_list[[23]],
                        legend_ab, ncol=6, nrow=4)
dev.off()
```

AB Figure S9

3-panel sensitivity

```
# Create dataset of case counts by day
ABcases <- incidence2::incidence(linelistAB, date_index = Date.Infecte
d) %>%
  tidyr::complete(date_index = seq.Date(          # ensure all dates a
re represented
```

```
    from = min(date_index, na.rm = T),
    to = max(date_index, na.rm=T),
    by = "day"),
    fill = list(count = 0)) %>%                    # convert NA counts
to 0
  rename(I = count,                                 # rename to names ex
pected by estimateR
         dates = date_index)

# Set mean and stand deviation for case counts
config_lit <- make_config(
  mean_si = 4.6,
  std_si = 5.55
)

# Estimate R for - Epi curves, R, and SI distribution
epiestim_res_litAB <- estimate_R(
  incid = ABcases,
  method = "parametric_si",
  config = config_lit
)

### median Rt
dfAB<-data.frame(epiestim_res_litAB$dates[8:length(epiestim_res_litAB$
dates)],
                 epiestim_res_litAB$R$`Median(R)`,
                 epiestim_res_litAB$R$`Quantile.0.025(R)`,
                 epiestim_res_litAB$R$`Quantile.0.975(R)`)
colnames(dfAB)<-c("dates", "median", "lower", "upper")
w_AB <-ggplot(data=dfAB) +
  geom_line(aes(x=dates, y=median))+
  geom_ribbon(aes(x=dates, ymin=lower,
                  ymax=upper), fill="grey70", alpha=0.5)

d_AB <-w_AB+theme_classic()+
  labs(title="Alberta",x=" ",y=expression(R[t]))+
  theme(legend.position="bottom")+
  scale_x_date(breaks=date_breaks("3 months"),
               labels=date_format("%b %Y"),
               limits=c(as.Date("2020-01-01"), as.Date("2022-02-01")))
+
  scale_y_continuous(limits = c(0,8))+
  geom_hline(yintercept=1.0, color="red",linetype="dotted") +
  theme_cowplot(font_size = 10)+
  theme(legend.position = "bottom")
```

```
# Removing Y-axis limit for manuscript figure 3
d_AB1 <-w_AB+theme_cowplot(font_size = 12)+
  labs(title="",x=" ",y=expression(R[t]))+
  theme(legend.position="bottom")+
  scale_x_date(breaks=date_breaks("3 months") , labels=date_format("%b
 %Y"),
               limits=c(as.Date("2019-11-29"), as.Date("2022-01-27")))
+
  scale_y_continuous(limits = c(0,8))+
  geom_hline(yintercept=1.0, color="red",linetype="dotted") +
  theme_cowplot(font_size = 10)+
  theme(legend.position = "bottom")


### Multiplier: Poisson distribution with a mean of 4
set.seed(1234)
# Generating a matrix of random numbers: each column (x-axis) is a day
, and
# each row (y-axis) is an iteration (e.g., you may have n=1000 simulat
ions)
poisAB<-matrix(rpois(n= (nrow (ABcases)*1000), lambda=4),
               ncol=nrow (ABcases))

# Renaming matrix columns and rows
colnames(poisAB) <- colnames(poisAB, do.NULL = FALSE,
                             prefix = "day.")
rownames(poisAB) <- rownames(poisAB, do.NULL = FALSE,
                             prefix = "iteration.")

# Multiply the daily new case count by each row of the matrix to gener
ate n
# simulations of the epi curve (estimated number of infections).
# Apply sweep operation that multiplies row by column multiplication
# (MARGIN=2) between poisAB and ABcases$I
save.resAB<-(sweep(poisAB, MARGIN=2,ABcases$I, `*`))

# Calculate the median values for each day (by column)
EstInf_too1AB<-apply(save.resAB,2,median)

# Calculating 95% CrI
EstInf_lowerAB <- apply(save.resAB,2, quantile, probs=0.025)
EstInf_upperAB <- apply(save.resAB,2, quantile, probs=0.975)

# Create dataframe to for the medians and CrI
EstInf_dfAB <- data.frame(EstInf_too1AB, EstInf_lowerAB, EstInf_upperA
B)
```

```r
# Preparing data for EpiEstim
newdataAB<-as.matrix.data.frame(save.resAB)

# Run your EpiEstim on each of these simulated epi curves.
test_resAB<-vector(mode="list", length = nrow(newdataAB)) # list

for(i in 1:nrow(newdataAB)){
  test_resAB [[i]]<- estimate_R(newdataAB [i,],
                                method="parametric_si",
                                config=confit_lit)
}

r_m4<-test_resAB
Sample3<-ABcases

# Generate 10 random values from the posterior R distribution
# for each time step for each hypothetical time series
sample_r_m4<-EpiEstim::sample_posterior_R(r_m4[[1]],
                                          n      = n,
                                          window = 1L)
for(v in 2:length(r_m4)){
  sample_r_m4<-append(sample_r_m4,
                      sample_posterior_R(r_m4[[v]],
                                         n      = n,
                                         window = 1L))
}
sample_r_m4_df<-sample_r_m4
for(window_number in 2:length(r_m4[[1]]$R$t_start)){
  sample_r_m4<-sample_posterior_R(r_m4[[1]],
                                  n      = n,
                                  window = window_number)
  for(v in 2:length(r_m4)){
    sample_r_m4<-append(sample_r_m4,
                        sample_posterior_R(r_m4[[v]],
                                           n      = n,
                                           window = window_number))
  }
  sample_r_m4_df<-rbind(sample_r_m4_df, sample_r_m4)
}

sample_r_m4_df<-as.data.frame(sample_r_m4_df)

sample_r_m4_df<-cbind(Sample3$dates[(r_m4[[1]]$R$t_end[1]):nrow(Sample
3)],
                      sample_r_m4_df)
```

```r
colnames(sample_r_m4_df)[1]<-"dates"

sample_r_m4_df <- sample_r_m4_df %>%
  rowwise() %>%
  mutate(Median=median(c_across(contains("V")))) %>%
  mutate(Lower=quantile(c_across(contains("V")), probs=0.025, na.rm=F,
 names=F)) %>%
  mutate(Upper=quantile(c_across(contains("V")), probs=0.975, na.rm=F,
 names=F)) %>%
  ungroup()  %>%
  dplyr::select(dates, Median, Lower, Upper, everything())

AB4_sample_r_m4_df <- sample_r_m4_df

M4_p3 <- ggplot(data    = AB4_sample_r_m4_df,
                mapping = aes(x = dates,
                              y = Median))+
  geom_line(col="black") +
  geom_ribbon(aes(ymin = Lower,
                  ymax = Upper),
              alpha = 0.2)

M4_g3 <- M4_p3 + labs(y = expression(R[t]),
                      x = "") +
  geom_hline(yintercept = 1,
             linetype   = 2) +
  theme_classic() +
  theme_cowplot(font_size = 10)+
  scale_x_date(breaks = date_breaks("3 months"),
               labels = date_format("%b %Y"),
               limits = c(as.Date("2020-01-01"),
                          as.Date("2022-02-01")))
M4_g3
(M4_g3b<-M4_g3+scale_y_continuous(limits=c(0,8)))

### Multiplier: Poisson distribution with a mean of 11
# Generating a matrix of random numbers: each column (x-axis) is a day
, and each
# row (y-axis) is an iteration (e.g., you may have n=1000 simulations)
poisAB11<-matrix(rpois(n= (nrow (ABcases)*1000), lambda=11), ncol=nrow
 (ABcases))

# naming matrix columns and rows
colnames(poisAB11) <- colnames(poisAB11, do.NULL = FALSE, prefix = "da
y.")
```

```r
rownames(poisAB11) <- rownames(poisAB11, do.NULL = FALSE, prefix = "it
eration.")

# Estimated number of infections
# Multiply the daily new case count by each row of the matrix to gener
ate n
# simulations of the epi curve (estimated number of infections).
# Apply sweep operation that multiplies row by column multiplication
# (MARGIN=2) between poisAB11 and ABcases$I
save.resAB11<-(sweep(poisAB11, MARGIN=2,ABcases$I, `*`))

# calculate the median values for each day (by column)
EstInf_medianAB11<-apply(save.resAB11,2,median)

# Calculating 95% CrI
EstInf_lowerAB11<- apply(save.resAB11,2, quantile, probs=0.025)
EstInf_upperAB11 <-apply(save.resAB11,2, quantile, probs=0.975)

# create data frame for the medians and CrI of Estimated number of inf
ection
EstInf_dfAB11 <- data.frame(EstInf_medianAB11, EstInf_lowerAB11, EstIn
f_upperAB11)

# preparing data for EpiEstim
newdataAB11<-as.matrix.data.frame(save.resAB11)

# Run your EpiEstim on each of these simulated epi curves.
# Create a blank list
test_resAB11<-vector(mode="list", length = nrow(newdataAB11)) #list

# Save the EpiEstim::estimate_R output object into the list
for(i in 1:nrow(newdataAB11)){
  test_resAB11 [[i]]<- estimate_R(newdataAB11 [i,],
                                 method="parametric_si",
                                 config=config_lit)
}

r_m5<-test_resAB11


# Generate 10 random values from the posterior R distribution
# for each time step for each hypothetical time series
sample_r_m5<-EpiEstim::sample_posterior_R(r_m5[[1]],
                                         n      = n,
                                         window = 1L)
for(v in 2:length(r_m5)){
```

```
  sample_r_m5<-append(sample_r_m5,
                      sample_posterior_R(r_m5[[v]],
                                         n      = n,
                                         window = 1L))
}
sample_r_m5_df<-sample_r_m5
for(window_number in 2:length(r_m5[[1]]$R$t_start)){
  sample_r_m5<-sample_posterior_R(r_m5[[1]],
                                  n      = n,
                                  window = window_number)
  for(v in 2:length(r_m5)){
    sample_r_m5<-append(sample_r_m5,
                        sample_posterior_R(r_m5[[v]],
                                           n      = n,
                                           window = window_number))
  }
  sample_r_m5_df<-rbind(sample_r_m5_df, sample_r_m5)
}

sample_r_m5_df<-as.data.frame(sample_r_m5_df)

sample_r_m5_df<-cbind(Sample3$dates[(r_m5[[1]]$R$t_end[1]):nrow(Sample
3)],
                      sample_r_m5_df)

colnames(sample_r_m5_df)[1]<-"dates"

sample_r_m5_df <- sample_r_m5_df %>%
  rowwise() %>%
  mutate(Median=median(c_across(contains("V")))) %>%
  mutate(Lower=quantile(c_across(contains("V")), probs=0.025, na.rm=F,
 names=F)) %>%
  mutate(Upper=quantile(c_across(contains("V")), probs=0.975, na.rm=F,
 names=F)) %>%
  ungroup()  %>%
  dplyr::select(dates, Median, Lower, Upper, everything())

AB11_sample_r_m5_df <-sample_r_m5_df

M11_p3 <- ggplot(data    = AB11_sample_r_m5_df,
             mapping = aes(x = dates,
                           y = Median))+
  geom_line(col="black") +
  geom_ribbon(aes(ymin = Lower,
                  ymax = Upper),
              alpha = 0.2)
```

```
M11_g3 <- M11_p3 + labs(y = expression(R[t]),
                x = "Assumed date of infection") +
  geom_hline(yintercept = 1,
             linetype   = 2) +
  theme_classic() +
  theme_cowplot(font_size = 10)+
  scale_x_date(breaks = date_breaks("3 months"),
               labels = date_format("%b %Y"),
               limits = c(as.Date("2020-01-01"),
                          as.Date("2022-02-01")))
M11_g3
(M11_g3b<-M11_g3+scale_y_continuous(limits=c(0,8)))


### Export Figure S9
tiff(file="AB_sensitivity figure_v4.tif",
     width=12, height=12,
     units='in', res=300)
pdf(file="AB_sensitivity figure_v4.pdf", width=12, height=12)
ggarrange (plot(d_AB),    # Rt based on case count
           plot(M4_g3b),  # Rt based on estimated infection count
                          # (Multiplier = 4)
           plot(M11_g3b), # Rt based on estimated infection count
                          # (Multiplier = 11)
           common.legend =  TRUE, legend = "bottom",
           ncol =1, nrow=3,align="v")

dev.off()
```

AB Figure 3

```
# Create a case count data set
counts_ab <- linelistAB %>% count(Date.Infected) %>% drop_na(Date.Infe
cted)

rolling_counts_ab <- counts_ab %>%   # Generate new data set with
                                     # a 7-day sliding average daily c
ounts
  mutate(n = slider::slide_dbl(      # Create new columns using slide_
dbl()
    n,                               # Calculate new percentage change
    .f = ~mean(.x, na.rm = F),       # Function is mean
                                     # (with missing values NOT remove
d)
    .before = 6))                    # Each sliding window is
                                     # the row and 6 prior rows
```

```
###  7-day avg incidence
p_abI <- plot(epiestim_res_litAB, "incid",
              options_I=list(xlab="Assumed date of infection"),
              legend = F)

d_abI <-p_abI+
  labs(title="Alberta", x="",y="Daily number of new cases")+
  geom_line(data=rolling_counts_ab,aes(x=Date.Infected,y=n) )+
          #7-day rolling average line
  scale_x_date(breaks=date_breaks("3 months") ,
               labels=date_format("%b %Y"),
               limits=c(as.Date("2019-11-29"),
                        as.Date("2022-01-27")))+
  theme_cowplot(font_size = 12)


###  incidence by variant
# Create ON variant data set
VarsAB <- read.csv("covid19-epiSummary-variants Alberta.csv", header=T
)

# Format date reported YYYY-MM-DD
VarsAB$dates<-as.Date(VarsAB$dates, "%m/%d/%Y")

# Create date of infection (9 day shift)
VarsAB$date_infected<-VarsAB$dates-9

# Remove unnecessary variables
VarsAB = subset(VarsAB, select = -c(dates))

# Rename variables
VarsAB <- VarsAB %>%
  rename(date=date_infected)

# Create plot
d_abV1<-ggplot(VarsAB, aes(date)) +theme_cowplot(font_size = 12)+
  labs(title="",x="", y="Daily number of new cases")+
  theme(legend.position="bottom")+
  scale_x_date(breaks=date_breaks("3 months"),
               labels=date_format("%b %Y"),
               limits=c(as.Date("2019-11-29"),
                        as.Date("2022-01-27"))) +
  geom_line(aes(y = WT, color = "WT")) +
  geom_line(aes(y = Alpha, color = "Alpha")) +
  geom_line(aes(y = Delta, color = "Delta")) +
```

```r
  geom_line(aes(y = Omicron, color = "Omicron")) +
  geom_line(aes(y = Other, color = "Other")) +
  scale_color_manual(name = "Variant",values = c("WT"      = "purple",
                                                 "Alpha"   = "red",
                                                 "Delta"   = "green",
                                                 "Omicron"= "blue",
                                                 "Other"   = "dimgrey")
)


###  Rt policy change and variant emergence
t_start_AB <- c(2,18,42,196,297,318,412,506,535,629)

# t_end is the day before the next policy implementation
t_end_AB <- c(17,41,195,296,317,411,505,534,628,693)

# Estimate R based on discrete policy windows instead of 7-day sliding
 window
AB_policy_res_parametric_si <- estimate_R(ABcases,
                                          method="parametric_si",
                                          config=make_config(list(
                                            t_start = t_start_AB,
                                            t_end = t_end_AB,
                                            mean_si=4.60,
                                            std_si=5.55)))
# Initial plot
p_abP <- plot(AB_policy_res_parametric_si, "R",
              options_I=list(xlab="Assumed date of infection"),
              legend = F)

# Creation of alpha-labels
c=data.frame(date=as.Date(c("2020-03-14",
                            "2020-04-07",
                            "2020-09-08",
                            "2020-12-18",
                            "2021-01-08",
                            "2021-04-12",
                            "2021-07-15",
                            "2021-08-13",
                            "2021-11-15")),
              y=c(0.5,0.6,0.45,0,0,0,0,0,0),
              event=c("A","B","C","D","E","F","G","H","I"))

# Create complete plot
g_abP <-p_abP+  theme_classic()+
  theme_cowplot(font_size = 12)+
```

```
  theme(legend.position="none")+
  labs(title=" ",
       x="Assumed date of infection",
       y=expression('Policy Change R'[t]))+
  scale_x_date(breaks=date_breaks("3 months"),
               labels=date_format("%b %Y"),
               limits=c(as.Date("2019-12-01"),
                        as.Date("2022-02-01"))) +

  geom_hline(yintercept=1, color="red",linetype="dotted")+
  geom_vline(data=c, mapping=aes(xintercept=date), color="blue", linet
ype="dotted") +

  #labels - nothing to change
  annotate(geom = "label",x=as.Date(c$date),y=c$y,label=c$event,size=2
,
           fontface="bold",color="blue",check_overlap=TRUE)


### Export tiff file for Figure 3
tiff(file="AB_combined figure_v4.tif", width=12, height=12, units='in'
, res=300)
pdf(file="AB_combined figure_v4.pdf", width=12, height=12)
ggpubr::ggarrange (plot(d_abI), #incident+7-day rolling average
                   plot(d_abV1), #variant
                   plot(d_AB1), #Rt
                   plot(g_abP), #policy change Rt
                   ncol =1, nrow=4, align="v")
dev.off()
```

AB Percent Change for Policy Change Rt

```
set.seed(12345)
test_res_AB <- AB_policy_res_parametric_si

test_res_AB$R$t_start
(number_of_windows<-length(test_res_AB$R$t_start))
# Create empty lists to store the medians, 95% CrI, and 1000 random va
lues from
# the posterior R distribution
W_Median_list_AB           <-rep(NA,
                                 number_of_windows) #vector
W_CrI_list_AB              <-vector(mode="list",
                                    length = number_of_windows) #list
W_R_list_AB                <-vector(mode="list",
                                    length = number_of_windows) #list
W_R_sample_change_list_AB  <-vector(mode="list",
```

```
                                      length = number_of_windows-1) #lis
t
W_R_sample_change_median_AB <-rep(NA,
                                  number_of_windows-1) #vector
W_R_sample_change_CrI_AB <-vector(mode="list",
                                  length = number_of_windows-1) #list
W_R_sample_percentage_change_list_AB <-vector(mode="list",
                                              length = number_of_windo
ws-1) #list
W_R_sample_percentage_change_median_AB <-rep(NA,
                                             number_of_windows-1) #vec
tor
W_R_sample_percentage_change_CrI_AB <-vector(mode="list",
                                             length = number_of_window
s-1) #list

# for loop
for(w in 1:number_of_windows){
  (W_Median_list_AB[w] <- test_res_AB$R$'Median(R)'[w])
  (W_CrI_list_AB[[w]]<- c(test_res_AB$R$'Quantile.0.025(R)'[w],

                        test_res_AB$R$'Quantile.0.975(R)'[w]))
  #Generate 1000 random values from the posterior R distribution
  W_R_list_AB[[w]] <- sample_posterior_R(test_res_AB, n=1000, window=w
)
}


for(w in 1:(number_of_windows-1)){
  # Change as a fraction of the Rt during the previous window
  # Median and 95% credible intervals of the distribution of R_sample_
change
  # Convert to Percentage change
  # Multiply by 100 to get the percentage
  W_R_sample_change_list_AB[[w]]<-(W_R_list_AB[[w+1]]-W_R_list_AB[[w]]
)/W_R_list_AB[[w]]
  W_R_sample_change_median_AB[w]<-median(W_R_sample_change_list_AB[[w]
])
  W_R_sample_change_CrI_AB[[w]] <-c(quantile(W_R_sample_change_list_AB
[[w]],
                                             probs=c(0.025, 0.975)))
  W_R_sample_percentage_change_list_AB[[w]]<-100*W_R_sample_change_lis
t_AB[[w]]
  W_R_sample_percentage_change_median_AB[w]<-100*W_R_sample_change_med
ian_AB[w]
  W_R_sample_percentage_change_CrI_AB[[w]] <-100*W_R_sample_change_CrI
```

```
_AB[[w]]
}
## Meaning: (R_next - R_previous) / R_previous
## R_next minus R_previous
## If this is positive, it means R_next > R_previous (i.e., increase)
## If this is negative, it means R_next
W_Median_list_AB
W_CrI_list_AB
W_R_sample_percentage_change_median_AB
W_R_sample_percentage_change_CrI_AB
```

Rt by variant

Ontario Figure 4

```
### wild-type
# Create WT dataset
wtON <- subset(VarsON, select = c(date, WT))
wtON <- rename(wtON, I=WT)
wtON$dates<-as.Date(wtON$date, "%m/%d/%Y")

# WT in ON was reported from 2020-02-15 to 2021-09-09 subset the datas
et after
# the end date
wtON <- subset(wtON, c(wtON$date < "2021-09-10"))

# Estimate R
res_parameric_wtON <- estimate_R(wtON,
                                 method="parametric_si",
                                 config=config_lit)
res_parameric_wtON$dates <- as.Date(res_parameric_wtON$date, "%m/%d/%Y
")

WT_ON <- plot(res_parameric_wtON, "R",
             options_I=list(xlab="Assumed date of infection"),
             legend = F)

# Use to plot with standard theme
WT_ON1 <-WT_ON+theme_classic()+
  labs(title="Wild Type", x="",
       y=expression(R[t]))+
  theme(legend.position = "none")+
  scale_x_date(breaks=date_breaks("3 months"),
               labels=date_format("%b %Y"),
               limits=c(as.Date("2019-11-29"),
                        as.Date("2022-01-27")))+
```

```
  scale_y_continuous(limits = c(0,7))+
  geom_hline(yintercept=1, color="red", linetype="dotted")

### Alpha
# Create Alpha dataset
alON <- subset(VarsON, select = c(date, Alpha))
alON <- rename(alON, I=Alpha)
alON$dates <- as.Date(alON$date, "%m/%d/%Y")

# Alpha in ON was most consistently reported from 2020-11-20 to 2021-1
0-07
# subset the dataset after the end date
alON <- subset(alON, c(alON$date > "2020-11-19"))
alON <- subset(alON, c(alON$date < "2021-09-08"))

# Estimate R
res_parameric_alON <- estimate_R(alON,
                                 method="parametric_si",
                                 config=config_lit)
res_parameric_alON$dates <- as.Date(res_parameric_alON$date, "%m/%d/%Y
")

AL_ON <- plot(res_parameric_alON, "R",
              options_I=list(xlab="Assumed date of infection"),
              legend = F)

# Use to plot with standard theme
AL_ON1 <-AL_ON+theme_classic()+
  labs(title="Alpha", x="",
       y=expression(R[t]))+
  theme(legend.position = "none")+
  scale_x_date(breaks=date_breaks("3 months"),
               labels=date_format("%b %Y"),
               limits=c(as.Date("2019-11-29"),
                        as.Date("2022-01-27")))+
  scale_y_continuous(limits = c(0,7))+
  geom_hline(yintercept=1, color="red",linetype="dotted")


### delta
# Create Delta dataset
dlON <- subset(VarsON, select = c(date, Delta))
dlON <- rename(dlON, I=Delta)
dlON$dates <- as.Date(dlON$date, "%m/%d/%Y")

# Delta in ON was most consistently reported from 2021-03-12 to 2022-0
```

```
1-18 (end
# of study period) subset the dataset after the end date
dlON <- subset(dlON, c(dlON$date > "2021-03-11"))

# Estimate R
res_parameric_dlON <- estimate_R(dlON,
                                 method="parametric_si",
                                 config=config_lit)
res_parameric_dlON$dates <- as.Date(res_parameric_dlON$date, "%m/%d/%Y
")

DL_ON <- plot(res_parameric_dlON, "R",
              options_I=list(xlab="Assumed date of infection"),
              legend = F)

# Use to plot with standard theme
DL_ON1 <-DL_ON+theme_classic()+
  labs(title="Delta", x="",
       y=expression(R[t]))+
  theme(legend.position = "none")+
  scale_x_date(breaks=date_breaks("3 months"),
               labels=date_format("%b %Y"),
               limits=c(as.Date("2019-11-29"),
                        as.Date("2022-01-27")))+
  scale_y_continuous(limits = c(0,7))+
  geom_hline(yintercept=1, color="red",linetype="dotted")


### omicron
# Create Omicron dataset
omON <- subset(VarsON, select = c(date, Omicron))
omON <- rename(omON, I=Omicron)
omON$dates <- as.Date(omON$date, "%m/%d/%Y")

# Omicron in ON was reported from 2021-11-12 to 2022-01-18 (end
# of study period) subset the dataset after the end date
omON <- subset(omON, c(omON$date > "2021-11-11"))

# Estimate R
res_parameric_omON <- estimate_R(omON,
                                 method="parametric_si",
                                 config=config_lit)
res_parameric_omON$dates <- as.Date(res_parameric_omON$date, "%m/%d/%Y
")

OM_ON <- plot(res_parameric_omON, "R",
```

```
              options_I=list(xlab="Assumed date of infection"),
              legend = F)

# Use to plot with standard theme
OM_ON1 <- OM_ON+ theme_classic()+
  labs(title="Omicron",
       x="Assumed date of infection",
       y=expression(R[t]))+
  theme(legend.position = "none")+
  scale_x_date(breaks=date_breaks("3 months"),
               labels=date_format("%b %Y"),
               limits=c(as.Date("2019-11-29"),
                        as.Date("2022-01-27")))+
  scale_y_continuous(limits = c(0,7))+
  geom_hline(yintercept=1, color="red",linetype="dotted")

# Print tiff file for Figure 4
tiff(file="ON_variants_v4.tif", width=12, height=12, units='in', res=3
00)
pdf(file="ON_variants_v4.pdf", width=12, height=12)
ggpubr::ggarrange (plot(WT_ON1),
           plot(AL_ON1),
           plot(DL_ON1),
           plot(OM_ON1),
           ncol =1, nrow=4, align="v")
dev.off()
```

British Columbia Figure 5

```
### wild-type
# Create WT dataset
wtBC <- subset(VarsBC, select = c(date, WT))
wtBC <- rename(wtBC, I=WT)
wtBC$dates<-as.Date(wtBC$date, "%m/%d/%Y")

# WT in ON was reported from 2020-02-15 to 2021-09-09 subset the datas
et after
# the end date
wtBC <- subset(wtBC, c(wtBC$date < "2021-09-10"))

# Estimate R
res_parameric_wtBC <- estimate_R(wtBC,
                                method="parametric_si",
                                config=config_lit)
res_parameric_wtBC$dates <- as.Date(res_parameric_wtBC$date, "%m/%d/%Y
")
```

```r
WT_BC <- plot(res_parameric_wtBC, "R",
              options_I=list(xlab="Assumed date of infection"),
              legend = F)

# Use to plot with standard theme
WT_BC1 <- WT_BC+
  theme_classic()+
  labs(title="Wild Type", x="",
       y=expression(R[t]))+
  theme(legend.position = "none")+
  scale_x_date(breaks=date_breaks("3 months"),
               labels=date_format("%b %Y"),
               limits=c(as.Date("2019-11-29"),
                        as.Date("2022-01-27")))+
  scale_y_continuous(limits = c(0,7))+
  geom_hline(yintercept=1, color="red", linetype="dotted")


### alpha
# Create Alpha dataset
alBC <- subset(VarsBC, select = c(date, Alpha))
alBC <- rename(alBC, I=Alpha)
alBC$dates <- as.Date(alBC$date, "%m/%d/%Y")

# Alpha in ON was most consistently reported from 11/20/2020 - 10/7/20
21
# subset the dataset after the end date
alBC <- subset(alBC, c(alBC$date > "2020-11-19"))
alBC <- subset(alBC, c(alBC$date < "2021-10-08"))

# Estimate R
res_parameric_alBC <- estimate_R(alBC,
                                 method="parametric_si",
                                 config=config_lit)
res_parameric_alBC$dates <- as.Date(res_parameric_alBC$date, "%m/%d/%Y
")

AL_BC <- plot(res_parameric_alBC, "R",
              options_I=list(xlab="Assumed date of infection"),
              legend = F)

# Use to plot with standard theme
AL_BC1 <-AL_BC+
  theme_classic()+
  labs(title="Alpha", x="",
       y=expression(R[t]))+
```

```
  theme(legend.position = "none")+
  scale_x_date(breaks=date_breaks("3 months"),
               labels=date_format("%b %Y"),
               limits=c(as.Date("2019-11-29"),
                        as.Date("2022-01-27")))+
  scale_y_continuous(limits = c(0,7))+
  geom_hline(yintercept=1, color="red",linetype="dotted")


### delta
# Create Delta dataset
dlBC <- subset(VarsBC, select = c(date, Delta))
dlBC <- rename(dlBC, I=Delta)
dlBC$dates <- as.Date(dlBC$date, "%m/%d/%Y")

# Delta in ON was most consistently reported from 3/12/2021 - 1/18/202
2 (end
# of study period) subset the dataset after the end date
dlBC <- subset(dlBC, c(dlBC$date > "2021-03-11"))

# Estimate R
res_parameric_dlBC <- estimate_R(dlBC,
                                 method="parametric_si",
                                 config=config_lit)
res_parameric_dlBC$dates <- as.Date(res_parameric_dlBC$date, "%m/%d/%Y
")

DL_BC <- plot(res_parameric_dlBC, "R",
              options_I=list(xlab="Assumed date of infection"),
              legend = F)

# Use to plot with standard theme
DL_BC1 <-DL_BC+
  theme_classic()+
  labs(title="Delta", x="",
       y=expression(R[t]))+
  theme(legend.position = "none")+
  scale_x_date(breaks=date_breaks("3 months"),
               labels=date_format("%b %Y"),
               limits=c(as.Date("2019-11-29"),
                        as.Date("2022-01-27")))+
  scale_y_continuous(limits = c(0,7))+
  geom_hline(yintercept=1, color="red",linetype="dotted")


### omicron
```

```r
# Create Omicron dataset
omBC <- subset(VarsBC, select = c(date, Omicron))
omBC <- rename(omBC, I=Omicron)
omBC$dates <- as.Date(omBC$date, "%m/%d/%Y")

# Omicron in ON was reported from 11/12/2021 - 1/18/2022 (end
# of study period) subset the dataset after the end date
omBC <- subset(omBC, c(omBC$date > "2021-11-11"))

# Estimate R
res_parameric_omBC <- estimate_R(omBC,
                                 method="parametric_si",
                                 config=config_lit)
res_parameric_omBC$dates <- as.Date(res_parameric_omBC$date, "%m/%d/%Y
")

OM_BC <- plot(res_parameric_omBC, "R",
              options_I=list(xlab="Assumed date of infection"),
              legend = F)

# Use to plot with standard theme
OM_BC1 <-OM_BC+
  theme_classic()+
  labs(title="Omicron",
       x="Assumed date of infection",
       y=expression(R[t]))+
  theme(legend.position = "none")+
  scale_x_date(breaks=date_breaks("3 months"),
               labels=date_format("%b %Y"),
               limits=c(as.Date("2019-11-29"),
                        as.Date("2022-01-27")))+
  scale_y_continuous(limits = c(0,7))+
  geom_hline(yintercept=1, color="red",linetype="dotted")

# Print tiff file for Figure 5
tiff(file="BC_variants_v4.tif",
     width=12, height=12,
     units='in', res=300)
pdf(file="BC_variants_v4.pdf", width=12, height=12)
ggpubr::ggarrange (plot(WT_BC1),
                   plot(AL_BC1),
                   plot(DL_BC1),
                   plot(OM_BC1),
                   ncol =1, nrow=4, align="v")
dev.off()
```

Alberta Figure 6

```
### wild-type
# Create WT dataset
wtAB <- subset(VarsAB, select = c(date, WT))
wtAB <- rename(wtAB, I=WT)
wtAB$dates<-as.Date(wtAB$date, "%m/%d/%Y")

# WT in ON was reported from 2/26/2020 - 9/9/2021 subset the dataset a
fter
# the end date
wtAB <- subset(wtAB, c(wtAB$date < "2021-09-10"))

# Estimate R
res_parameric_wtAB <- estimate_R(wtAB,
                                 method="parametric_si",
                                 config=config_lit)
res_parameric_wtAB$dates <- as.Date(res_parameric_wtAB$date, "%m/%d/%Y
")

WT_AB <- plot(res_parameric_wtAB, "R",
              options_I=list(xlab="Assumed date of infection"),
              legend = F)

# Use to plot with standard theme
WT_AB1 <-WT_AB+
  theme_classic()+
  labs(title="Wild Type", x="",
       y=expression(R[t]))+
  theme(legend.position = "none")+
  scale_x_date(breaks=date_breaks("3 months"),
               labels=date_format("%b %Y"),
               limits=c(as.Date("2019-11-29"),
                        as.Date("2022-01-27")))+
  scale_y_continuous(limits = c(0,7))+
  geom_hline(yintercept=1, color="red",linetype="dotted")

### alpha
# Create Alpha dataset
alAB <- subset(VarsAB, select = c(date, Alpha))
alAB <- rename(alAB, I=Alpha)
alAB$dates <- as.Date(alAB$date, "%m/%d/%Y")

# Alpha in ON was most consistently reported from 2020-11-20 to 2021-1
0-07
# subset the dataset after the end date
alAB <- subset(alAB, c(alAB$date > "2020-11-19"))
```

```
alAB <- subset(alAB, c(alAB$date < "2021-10-08"))

# Estimate R
res_parameric_alAB <- estimate_R(alAB,
                                 method="parametric_si",
                                 config=config_lit)
res_parameric_alAB$dates <- as.Date(res_parameric_alAB$date, "%m/%d/%Y
")

AL_AB <- plot(res_parameric_alAB, "R",
              options_I=list(xlab="Assumed date of infection"),
              legend = F)

# Use to plot with standard theme
AL_AB1 <-AL_AB+
  theme_classic()+
  labs(title="Alpha", x="",
       y=expression(R[t]))+
  theme(legend.position = "none")+
  scale_x_date(breaks=date_breaks("3 months"),
               labels=date_format("%b %Y"),
               limits=c(as.Date("2019-11-29"),
                        as.Date("2022-01-27")))+
  scale_y_continuous(limits = c(0,7))+
  geom_hline(yintercept=1, color="red", linetype="dotted")


### delta
# Create Delta dataset
dlAB <- subset(VarsAB, select = c(date, Delta))
dlAB <- rename(dlAB, I=Delta)
dlAB$dates <- as.Date(dlAB$date, "%m/%d/%Y")

# Delta in ON was most consistently reported from 3/12/2021 - 1/18/202
2 (end
# of study period) subset the dataset after the end date
dlAB <- subset(dlAB, c(dlAB$date > "2021-03-11"))

# Estimate R
res_parameric_dlAB <- estimate_R(dlAB,
                                 method="parametric_si",
                                 config=config_lit)
res_parameric_dlAB$dates <- as.Date(res_parameric_dlAB$date, "%m/%d/%Y
")

DL_AB <- plot(res_parameric_dlAB, "R",
```

```
              options_I=list(xlab="Assumed date of infection"),
              legend = F)

# Use to plot with standard theme
DL_AB1 <-DL_AB+
  theme_classic()+
  labs(title="Delta", x="",
       y=expression(R[t]))+
  theme(legend.position = "none")+
  scale_x_date(breaks=date_breaks("3 months"),
               labels=date_format("%b %Y"),
               limits=c(as.Date("2019-11-29"),
                        as.Date("2022-01-27")))+
  scale_y_continuous(limits = c(0,7))+
  geom_hline(yintercept=1, color="red", linetype="dotted")

### Omicron
# Create Omicron dataset
omAB <- subset(VarsAB, select = c(date, Omicron))
omAB <- rename(omAB, I=Omicron)
omAB$dates <- as.Date(omAB$date, "%m/%d/%Y")

# Omicron in ON was reported from 11/12/2021 - 1/6/2022 (end
# of study period) subset the dataset after the end date
omAB <- subset(omAB, c(omAB$date > "2021-11-11"))

# Estimate R
res_parameric_omAB <- estimate_R(omAB, method="parametric_si", config=
make_config(list(mean_si=4.60, std_si=5.55)))
res_parameric_omAB$dates <- as.Date(res_parameric_omAB$date, "%m/%d/%Y
")

OM_AB <- plot(res_parameric_omAB, "R",
              options_I=list(xlab="Assumed date of infection"),
              legend = F)

# Use to plot with standard theme
OM_AB1 <- OM_AB+theme_classic()+
  labs(title="Omicron", x="Assumed date of infection",
       y=expression(R[t]))+
  theme(legend.position = "none")+
  scale_x_date(breaks=date_breaks("3 months"),
               labels=date_format("%b %Y"),
               limits=c(as.Date("2019-11-29"),
                        as.Date("2022-01-27")))+
  scale_y_continuous(limits = c(0,7))+
```

```
  geom_hline(yintercept=1, color="red", linetype="dotted")


# Print tiff file for Figure 6
tiff(file="AB_variants_v4.tif",
     width=12, height=12,
     units='in', res=300)
pdf(file="AB_variants_v4.pdf", width=12, height=12)
ggpubr::ggarrange (plot(WT_AB1),
                   plot(AL_AB1),
                   plot(DL_AB1),
                   plot(OM_AB1),
                   ncol =1, nrow=4, align="v")
dev.off()
```

Table 2 - Variant Cumulative Case Count

Ontario - population data obtained from https://www12.statcan.gc.ca/census-recensement/2021/dp-pd/prof/details/page.cfm?Lang=E&SearchText=Ontario&DGUIDlist=2021A000235&GENDERlist=1,2,3&STATISTIClist=1&HEADERlist=0 Total population for all person-time calculations 14223942

British Columbia - population data obtained from https://www12.statcan.gc.ca/census-recensement/2021/dp-pd/prof/details/page.cfm?Lang=E&SearchText=Ontario&DGUIDlist=2021A000235&GENDERlist=1,2,3&STATISTIClist=1&HEADERlist=0 Total population for all person-time calculations 5000879

Alberta - population data obtained from https://www12.statcan.gc.ca/census-recensement/2021/dp-pd/prof/details/page.cfm?Lang=E&SearchText=Ontario&DGUIDlist=2021A000235&GENDERlist=1,2,3&STATISTIClist=1&HEADERlist=0 Total population for all person-time calculations 4262635

```
### Ontario
# Wild Type
# Creating dummy variable to create WT day count
VarsON$ctwt <- ifelse(VarsON$WT !=0, VarsON$date, NA)
VarsON$ctwt <- as.Date(VarsON$ctwt, origin="1970-01-01")

# Calculation for total number of days of data, +1 day for the last da
y
(max(VarsON$ctwt, na.rm = T)-min(VarsON$ctwt, na.rm = T))+1

# Calculation of total person time
WTptON <-((max(VarsON$ctwt, na.rm = T)-min(VarsON$ctwt, na.rm = T))+1)
```

```
*14223942

# Total wild type cases
WTsumON<-sum(VarsON$WT)

# Cumulative Case Counts per person days
# 337298.5/8136094824        # = 4.145705e-05
WTsumON/as.numeric(WTptON)    # = 4.145705e-05

# Alpha
# Creating dummy variable to create Alpha day count
VarsON$ctal <- ifelse(VarsON$Alpha !=0, VarsON$date, NA)
VarsON$ctal <- as.Date(VarsON$ctal, origin="1970-01-01")

# Calculation for total number of days of data, +1 for the last day
(max(VarsON$ctal, na.rm = T)-min(VarsON$ctal, na.rm = T))+1

# Calculation of total person time
ALptON <-((max(VarsON$ctal, na.rm = T)-min(VarsON$ctal, na.rm = T))+1)
*14223942

# Total alpha cases
ALsumON<-sum(VarsON$Alpha)

# Cumulative Case Counts per person days
# 133162.1/8562813084        # = 1.555121e-05
ALsumON/as.numeric(ALptON)    # = 1.555121e-05

# Delta
# Creating dummy variable to create Delta day count
VarsON$ctdl <- ifelse(VarsON$Delta !=0, VarsON$date, NA)
VarsON$ctdl <- as.Date(VarsON$ctdl, origin="1970-01-01")

# Calculation for total number of days of data, +1 for the last day
(max(VarsON$ctdl, na.rm = T)-min(VarsON$ctdl, na.rm = T))+1

# Calculation of total person time
DLptON<-((max(VarsON$ctdl, na.rm = T)-min(VarsON$ctdl, na.rm = T))+1)*
14223942

# Total delta cases
DLsumON<-sum(VarsON$Delta)

# Cumulative Case Counts per person days
# 121710.1/6543013320        # = 1.860154e-05
DLsumON/as.numeric(DLptON)    # = 1.860154e-05
```

```
# Omicron
# Creating dummy variable to create Omicron day count
VarsON$ctom <- ifelse(VarsON$Omicron !=0, VarsON$date, NA)
VarsON$ctom <- as.Date(VarsON$ctom, origin="1970-01-01")

# Calculation for total number of days of data, +1 for the last day
(max(VarsON$ctom, na.rm = T)-min(VarsON$ctom, na.rm = T))+1

# Calculation of total person time
OMptON<-((max(VarsON$ctom, na.rm = T)-min(VarsON$ctom, na.rm = T))+1)*
14223942

# Total omicron cases
OMsumON<-sum(VarsON$Omicron)

# Cumulative Case Counts per person days
# 366494.8/967228056        # = 0.0003789125
OMsumON/as.numeric(OMptON)    # = 0.0003789125


### British Columbia
VarsBC$ctwt <- ifelse(VarsBC$WT !=0, VarsBC$date, NA)
VarsBC$ctwt <- as.Date(VarsBC$ctwt, origin="1970-01-01")

# Calculation for total number of days of data, +1 for the last day
(max(VarsBC$ctwt, na.rm = T)-min(VarsBC$ctwt, na.rm = T))+1

# Calculation of total person time
WTptBC <-((max(VarsBC$ctwt, na.rm = T)-min(VarsBC$ctwt, na.rm = T))+1)
*5000879

# Total wild type cases
WTsumBC<-sum(VarsBC$WT)

# Cumulative Case Counts per person days
# 91169.94/2865503667       # = 3.181638e-05
WTsumBC/as.numeric(WTptBC)    # = 3.181637e-05

# Alpha
# Creating dummy variable to create Alpha day count
VarsBC$ctal <- ifelse(VarsBC$Alpha !=0, VarsBC$date, NA)
VarsBC$ctal <- as.Date(VarsBC$ctal, origin="1970-01-01")

# Calculation for total number of days of data
(max(VarsBC$ctal, na.rm = T)-min(VarsBC$ctal, na.rm = T))+1

# Calculation of total person time
```

```
ALptBC <-((max(VarsBC$ctal, na.rm = T)-min(VarsBC$ctal, na.rm = T))+1)
*5000879

# Total alpha cases
ALsumBC<-sum(VarsBC$Alpha)

# Cumulative Case Counts per person days
# 36951.2/3010529158       # = 1.227399e-05
ALsumBC/as.numeric(ALptBC)    # = 1.227399e-05

# Delta
# Creating dummy variable to create Delta day count
VarsBC$ctdl <- ifelse(VarsBC$Delta !=0, VarsBC$date, NA)
VarsBC$ctdl <- as.Date(VarsBC$ctdl, origin="1970-01-01")

# Calculation for total number of days of data
(max(VarsBC$ctdl, na.rm = T)-min(VarsBC$ctdl, na.rm = T))+1

# Calculation of total person time
DLptBC<-((max(VarsBC$ctdl, na.rm = T)-min(VarsBC$ctdl, na.rm = T))+1)*
5000879

# Total delta cases
DLsumBC<-sum(VarsBC$Delta)

# Cumulative Case Counts per person days
# 82440.92/2300404340       # = 3.583758e-05
DLsumBC/as.numeric(DLptBC)    # = 3.583758e-05

# Omicron
# Creating dummy variable to create Omicron day count
VarsBC$ctom <- ifelse(VarsBC$Omicron !=0, VarsBC$date, NA)
VarsBC$ctom <- as.Date(VarsBC$ctom, origin="1970-01-01")

# Calculation for total number of days of data
(max(VarsBC$ctom, na.rm = T)-min(VarsBC$ctom, na.rm = T))+1

# Calculation of total person time
OMptBC<-((max(VarsBC$ctom, na.rm = T)-min(VarsBC$ctom, na.rm = T))+1)*
5000879

# Total omicron cases
OMsumBC<-sum(VarsBC$Omicron)

# Cumulative Case Counts per person days
# 91490.87/340059772        # = 0.0002690435
OMsumBC/as.numeric(OMptBC)    # = 0.0002690435
```

```r
### Alberta
# Wild Type
# Creating dummy variable to create WT day count
VarsAB$ctwt <- ifelse(VarsAB$WT !=0, VarsAB$date, NA)
VarsAB$ctwt <- as.Date(VarsAB$ctwt, origin="1970-01-01")

# Calculation for total number of days of data, +1 for the last day
(max(VarsAB$ctwt, na.rm = T)-min(VarsAB$ctwt, na.rm = T))+1

# Calculation of total person time
WTptAB <-((max(VarsAB$ctwt, na.rm = T)-min(VarsAB$ctwt, na.rm = T))+1)
*4262635

# Total wild type cases
WTsumAB<-sum(VarsAB$WT)

# Cumulative Case Counts per person days
# 146910.3/2395600870       # = 6.132503e-05
WTsumAB/as.numeric(WTptAB)    # = 6.132503e-05

# Alpha
# Creating dummy variable to create Alpha day count
VarsAB$ctal <- ifelse(VarsAB$Alpha !=0, VarsAB$date, NA)
VarsAB$ctal <- as.Date(VarsAB$ctal, origin="1970-01-01")

# Calculation for total number of days of data
(max(VarsAB$ctal, na.rm = T)-min(VarsAB$ctal, na.rm = T))+1

# Calculation of total person time
ALptAB <-((max(VarsAB$ctal, na.rm = T)-min(VarsAB$ctal, na.rm = T))+1)
*4262635

# Total alpha cases
ALsumAB<-sum(VarsAB$Alpha)

# Cumulative Case Counts per person days
# 54621.08/2566106270       # = 2.128559e-05
ALsumAB/as.numeric(ALptAB)    # = 2.128559e-05

# Delta
# Creating dummy variable to create Delta day count
VarsAB$ctdl <- ifelse(VarsAB$Delta !=0, VarsAB$date, NA)
VarsAB$ctdl <- as.Date(VarsAB$ctdl, origin="1970-01-01")

# Calculation for total number of days of data
```

```
(max(VarsAB$ctdl, na.rm = T)-min(VarsAB$ctdl, na.rm = T))+1

# Calculation of total person time
DLptAB<-((max(VarsAB$ctdl, na.rm = T)-min(VarsAB$ctdl, na.rm = T))+1)*
4262635

# Total delta cases
DLsumAB<-sum(VarsAB$Delta)

# Cumulative Case Counts per person days
# 116901.5/1960812100       # = 5.961893e-05
DLsumAB/as.numeric(DLptAB)    # = 5.961893e-05

# Omicron
# Creating dummy variable to create Omicron day count
VarsAB$ctom <- ifelse(VarsAB$Omicron !=0, VarsAB$date, NA)
VarsAB$ctom <- as.Date(VarsAB$ctom, origin="1970-01-01")

# Calculation for total number of days of data
(max(VarsAB$ctom, na.rm = T)-min(VarsAB$ctom, na.rm = T))+1

# Calculation of total person time
OMptAB<-((max(VarsAB$ctom, na.rm = T)-min(VarsAB$ctom, na.rm = T))+1)*
4262635

# Total omicron cases
OMsumAB<-sum(VarsAB$Omicron)

# Cumulative Case Counts per person days
#141660.6/289859180        # = 0.0004887222
OMsumAB/as.numeric(OMptAB)    # = 0.0004887222


### Overall Totals
# Wild Type
# Calculation of total person time
WTpt <-(((max(VarsON$ctwt, na.rm = T)-min(VarsON$ctwt, na.rm = T))+1)*
14223942)+
       (((max(VarsBC$ctwt, na.rm = T)-min(VarsBC$ctwt, na.rm = T))+1)*
5000879)+
       (((max(VarsAB$ctwt, na.rm = T)-min(VarsAB$ctwt, na.rm = T))+1)*
4262635)

# Total wild type cases
WTsum<-sum(VarsAB$WT)+sum(VarsBC$WT)+sum(VarsON$WT)

# Cumulative Case Counts per person days
```

```
# 575378.8/13397199361       # = 4.294769e-05
WTsum/as.numeric(WTpt)    # = 4.294769e-05

# Alpha
# Calculation of total person time
ALpt <-(((max(VarsON$ctal, na.rm = T)-min(VarsON$ctal, na.rm = T))+1)*
14223942)+
       (((max(VarsBC$ctal, na.rm = T)-min(VarsBC$ctal, na.rm = T))+1)*
5000879)+
       (((max(VarsAB$ctal, na.rm = T)-min(VarsAB$ctal, na.rm = T))+1)*
4262635)

# Total alpha cases
ALsum<-sum(VarsAB$Alpha)+sum(VarsBC$Alpha)+sum(VarsON$Alpha)

# Cumulative Case Counts per person days
# 224734.4/14139448512       # = 1.589414e-05
ALsum/as.numeric(ALpt)    # = 1.589414e-05

# Delta
# Calculation of total person time
DLpt <-(((max(VarsON$ctdl, na.rm = T)-min(VarsON$ctdl, na.rm = T))+1)*
14223942)+
       (((max(VarsBC$ctdl, na.rm = T)-min(VarsBC$ctdl, na.rm = T))+1)*
5000879)+
       (((max(VarsAB$ctdl, na.rm = T)-min(VarsAB$ctdl, na.rm = T))+1)*
4262635)

# Total delta cases
DLsum<-sum(VarsAB$Delta)+sum(VarsBC$Delta)+sum(VarsON$Delta)

# Cumulative Case Counts per person days
# 321052.6/10804229760       # = 2.971545e-05
DLsum/as.numeric(DLpt)    # = 2.971545e-05

# Omicron
# Calculation of total person time
OMpt <-(((max(VarsON$ctom, na.rm = T)-min(VarsON$ctom, na.rm = T))+1)*
14223942)+
       (((max(VarsBC$ctom, na.rm = T)-min(VarsBC$ctom, na.rm = T))+1)*
5000879)+
       (((max(VarsAB$ctom, na.rm = T)-min(VarsAB$ctom, na.rm = T))+1)*
4262635)

# Total omicron cases
OMsum <-sum(VarsAB$Omicron)+sum(VarsBC$Omicron)+sum(VarsON$Omicron)
```

```
# Cumulative Case Counts per person days
# 599646.3/1597147008        # = 0.0003754484
OMsum/as.numeric(OMpt)    # = 0.0003754484
```

Table 2 - Variant by Province IRR

Calculate the incidence rate ratio (a kind of relative risk) and its confidence intervals based on approximation, followed by null hypothesis (incidence rate ratio equals 1) testing. package: fmsb fmsb::rateratio function is used to calculate incidence rate ratio and its confidence intervals

rateratio(a, b, PT1, PT0, conf.level=0.95)

Arguments a - The number of disease occurrences among exposed cohort. b - The number of disease occurrences among the non-exposed cohort. PT1 - The observed person-time of the exposed cohort. PT0 - The observed person-time of the unexposed cohort. conf.level - Probability for confidence intervals. The default is 0.95.

Value estimate - Calculated point estimate of incidence rate ratio. conf.int - A numeric vector of length 2 to give upper/lower limit of confidence intervals. p.value - The significant probability of the result of null-hypothesis testing.

```
### Ontario TOTALS
# Wild IR
337298.5/8136094824

# Alpha : Wild (ref)
133162.1/8562813084
res <- rateratio(133162.1, 337298.5, 8562813084, 8136094824, conf.leve
l=0.95)
str(res)
print(res)

# Delta : Wild (ref)
121710.1/6543013320
res <- rateratio(121710.1, 337298.5, 6543013320, 8136094824, conf.leve
l=0.95)
str(res)
print(res)

# Omicron : Wild (ref)
366494.8/967228056
res <- rateratio(366494.8, 337298.5, 967228056, 8136094824, conf.level
=0.95)
str(res)
print(res)
```

```
### British Columbia TOTALS
# Wild IR
91169.94/2865503667

# Alpha : Wild (ref)
36951.2/3010529158
res <- rateratio(36951.2, 91169.94, 3010529158, 2865503667, conf.level
=0.95)
str(res)
print(res)

# Delta : Wild (ref)
82440.92/2300404340
res <- rateratio(82440.92, 91169.94, 2300404340, 2865503667, conf.leve
l=0.95)
str(res)
print(res)

# Omicron : Wild (ref)
91490.87/340059772
res <- rateratio(91490.87, 91169.94, 340059772, 2865503667, conf.level
=0.95)
str(res)
print(res)

### Alberta TOTALS
# Wild IR
146910.3/2395600870

# Alpha : Wild (ref)
54621.08/2566106270
res <- rateratio(54621.08, 146910.3, 2566106270, 2395600870, conf.leve
l=0.95)
str(res)
print(res)

# Delta : Wild (ref)
116901.5/1960812100
res <- rateratio(116901.5, 146910.3, 1960812100, 2395600870, conf.leve
l=0.95)
str(res)
print(res)

# Omicron : Wild (ref)
141660.6/289859180
res <- rateratio(141660.6, 146910.3, 289859180, 2395600870, conf.level
```

```
=0.95)
str(res)
print(res)

### OVERALL TOTALS
# Wild IR
575378.8/13397199361

# Alpha : Wild (ref)
224734.4/14139448512
res <- rateratio(224734.4, 575378.8, 14139448512, 13397199361, conf.le
vel=0.95)
str(res)
print(res)

# Delta : Wild (ref)
321052.6/10804229760
res <- rateratio(321052.6, 575378.8, 10804229760, 13397199361, conf.le
vel=0.95)
str(res)
print(res)

# Omicron : Wild (ref)
599646.3/1597147008
res <- rateratio(599646.3, 575378.8, 1597147008, 13397199361, conf.lev
el=0.95)
str(res)
print(res)
```

Table 2 - Province by Variant IRR

Calculate the incidence rate ratio (a kind of relative risk) and its confidence intervals based on approximation, followed by null hypothesis (incidence rate ratio equals 1) testing. package: fmsb fmsb::rateratio function is used to calculate incidence rate ratio and its confidence intervals

rateratio(a, b, PT1, PT0, conf.level=0.95)

Arguments a - The number of disease occurrence among the exposed cohort. b - The number of disease occurrence among the non-exposed cohort. PT1 - The observed person-time of the exposed cohort. PT0 - The observed person-time of the unexposed cohort. conf.level - Probability for confidence intervals. The default is 0.95.

Value estimate - Calculated point estimate of incidence rate ratio. conf.int - A numeric vector of length 2 to give upper/lower limit of confidence intervals. p.value - The significant probability of the result of null-hypothesis testing.

```
### Wild Type
# British Columbia
```

```
91169.94/2865503667

# Ontario : British Columbia (ref)
337298.5/8136094824
res <- rateratio(337298.5, 91169.94, 8136094824, 2865503667)
str(res)
print(res)

# Alberta : British Columbia
146910.3/2395600870
res <- rateratio(146910.3, 91169.94, 2395600870, 2865503667)
str(res)
print(res)

### Alpha Variant
# British Columbia
36951.2/3010529158

# Ontario : British Columbia
133162.1/8562813084
res <- rateratio(133162.1, 36951.2, 8562813084, 3010529158, conf.level
=0.95)
str(res)
print(res)

# Alberta : British Columbia
54621.08/2566106270
res <- rateratio(54621.08, 36951.2, 2566106270, 3010529158, conf.level
=0.95)
str(res)
print(res)

### Delta Variant
# British Columbia
82440.92/2300404340

# Ontario : British Columbia
121710.1/6543013320
res <- rateratio(121710.1, 82440.92, 6543013320, 2300404340, conf.leve
l=0.95)
str(res)
print(res)

# Alberta : British Columbia
116901.5/1960812100
res <- rateratio(116901.5, 82440.92, 1960812100, 2300404340, conf.leve
```

```
l=0.95)
str(res)
print(res)

### Omicron Variant
# British Columbia
91490.87/340059772

# Ontario : British Columbia
366494.8/967228056
res <- rateratio(366494.8, 91490.87, 967228056, 340059772, conf.level=
0.95)
str(res)
print(res)

# Alberta : British Columbia
141660.6/289859180
res <- rateratio(141660.6, 91490.87, 289859180, 340059772, conf.level=
0.95)
str(res)
print(res)

################################# TOTAL All Variants
### British Columbia
# Total Cases
91169.94+36951.2+82440.92+91490.87            # = 302052.9
# Total Person Time
2865503667+3010529158+2300404340+340059772    # = 8516496937
# Cumulative Case Count
302052.9/8516496937                           # = 3.54668e-05

### Ontario
# Total Cases
337298.5+133162.1+121710.1+366494.8           # = 958665.5
# Total Person Time
8136094824+8562813084+6543013320+967228056    # = 24209149284
# Cumulative Case Count
958665.5/24209149284                          # = 3.959931e-05
# Ontario : British Columbia
res <- rateratio(958665.5, 302052.9, 24209149284, 8516496937, conf.lev
el=0.95)
str(res)
print(res)

### Alberta
# Total Cases
```

```
146910.3+54621.08+116901.5+141660.6          # = 460093.5
# Total Person Time
2395600870+2566106270+1960812100+289859180    # = 7212378420
#Cumulative Case Count
460093.5/7212378420                          # = 6.37922e-05
# Alberta : British Columbia
res <- rateratio(460093.5, 302052.9, 7212378420, 8516496937, conf.leve
l=0.95)
str(res)
print(res)

### Total
# Total Cases
302052.9+958665.5+460093.5                   # = 1720812
# Total Person Time
8516496937+24209149284+7212378420            # = 39938024641
#Cumulative Case Count
1720812/39938024641                          # = 4.308706e-05
```

Sample Characteristics - Tables S1, S2, S3

```
## Table S1 - Ontario
table(linelistON$Age, linelistON$Sex)

## Table S2 - British Columbia
table(linelistBC$Age, linelistBC$Sex)

## Table S3 - Alberta
table(linelistAB$Age, linelistAB$Sex)
```

Sex-Age IRR Tables S5, S6, S7

Calculate the incidence rate ratio (a kind of relative risk) and its confidence intervals based on approximation, followed by null hypothesis (incidence rate ratio equals to 1) testing.

rateratio(a, b, PT1, PT0, conf.level=0.95)

Arguments a - The number of disease occurrences among the exposed cohort. b - The number of disease occurrences among the non-exposed cohort. PT1 - The observed person-time of the exposed cohort. PT0 - The observed person-time of the unexposed cohort. conf.level - Probability for confidence intervals. The default is 0.95.

Value estimate - Calculated point estimate of incidence rate ratio. conf.int - A numeric vector of length 2 to give upper/lower limit of confidence intervals. p.value - The significant probability of the result of null-hypothesis testing.

```
#### Female ####
#### Total (Age)
```

```
# Cumulative Case Count per person days
# Alberta
248306/1479347100
# British Columbia
162082/1856656450
# Ontario
532418/1479347100

# IRR Females - Alberta : British Columbia (ref)
res <- rateratio(248306, 162082, 1479347100, 1856656450, conf.level=0.
95)
str(res)
print(res)

# IRR Females - Ontario : British Columbia (ref)
res <- rateratio(532418, 162082, 1479347100, 1856656450, conf.level=0.
95)
str(res)
print(res)


#### Age Groups
# Cumulative Case Counts per person days
# Alberta
# <20
54914/356746005
# 20-29
45993/178173765
# 30-39
50110/231215985
# 40-49
40778/202369860
# 50-59
27219/186725385
# 60-69
14941/169046955
# 70-79
6722/97293735
# 80+
7629/57775410

# British Columbia
# <20
29183/343169350
# 20-29
33854/226102900
```

```r
# 30-39
31604/261219550
# 40-49
24731/236355750
# 50-59
19116/258628050
# 60-69
11941/256682600
# 70-79
5759/173017300
# 80+
5894/101480950

# Ontario
# <20
88002/1092319680
# 20-29
113248/668501440
# 30-39
91373/720253280
# 40-49
80597/681756800
# 50-59
71795/749476160
# 60-69
40380/681179040
# 70-79
20378/287805440
# 80+
26645/290053920

#### IRR <20
# IRR Alberta : British Columbia (ref)
res <- rateratio(54914, 29183, 356746005, 343169350, conf.level=0.95)
str(res)
print(res)

# IRR Ontario : British Columbia (ref)
res <- rateratio(88002, 29183, 1092319680, 343169350, conf.level=0.95)
str(res)
print(res)

#### IRR 20-29
# IRR Alberta : British Columbia (ref)
res <- rateratio(45993, 33854, 178173765, 226102900, conf.level=0.95)
str(res)
```

```r
print(res)

# IRR Ontario : British Columbia (ref)
res <- rateratio(113248, 33854, 668501440, 226102900, conf.level=0.95)
str(res)
print(res)

#### IRR 30-39
# IRR Alberta : British Columbia (ref)
res <- rateratio(50110, 31604, 231215985, 261219550, conf.level=0.95)
str(res)
print(res)

# IRR Ontario : British Columbia (ref)
res <- rateratio(91373, 31604, 720253280, 261219550, conf.level=0.95)
str(res)
print(res)

#### IRR 40-49
# IRR Alberta : British Columbia (ref)
res <- rateratio(40778, 24731, 202369860, 236355750, conf.level=0.95)
str(res)
print(res)

# IRR Ontario : British Columbia (ref)
res <- rateratio(80597, 24731, 681756800, 236355750, conf.level=0.95)
str(res)
print(res)

#### IRR 50-59
# Alberta : British Columbia (ref)
res <- rateratio(27219, 19116, 186725385, 258628050, conf.level=0.95)
str(res)
print(res)

# IRR Ontario : British Columbia (ref)
res <- rateratio(71795, 19116, 749476160, 258628050, conf.level=0.95)
str(res)
print(res)

#### IRR 60-69
# IRR Alberta : British Columbia (ref)
res <- rateratio(14941, 11941, 169046955, 256682600, conf.level=0.95)
str(res)
print(res)
```

```r
# IRR Ontario : British Columbia (ref)
res <- rateratio(40380, 11941, 681179040, 256682600, conf.level=0.95)
str(res)
print(res)

#### IRR 70-79
# IRR Alberta : British Columbia (ref)
res <- rateratio(6722, 5759, 97293735, 173017300, conf.level=0.95)
str(res)
print(res)

# IRR Ontario : British Columbia (ref)
res <- rateratio(20378, 5759, 287805440, 173017300, conf.level=0.95)
str(res)
print(res)

#### IRR 80+
# IRR Alberta : British Columbia (ref)
res <- rateratio(7629, 5894, 57775410, 101480950, conf.level=0.95)
str(res)
print(res)

# IRR Ontario : British Columbia (ref)
res <- rateratio(26645, 5894, 290053920, 101480950, conf.level=0.95)
str(res)
print(res)


#### Male ####
#### Total (Age)
# Cumulative Case Count per person days
# Alberta
238441/1474658955
# British Columbia
157301/1793985950
# Ontario
495338/5035779712

# IRR Males - Alberta : British Columbia (ref)
res <- rateratio(238441, 157301, 1474658955, 1793985950, conf.level=0.
95)
str(res)
print(res)

# IRR Males - Ontario : British Columbia (ref)
res <- rateratio(495338, 157301, 5035779712, 1793985950, conf.level=0.
```

```
95)
str(res)
print(res)

#### Age Groups
# Cumulative Case Counts per person days
# Alberta
# <20
58130/397568950
# 20-29
42204/194986650
# 30-39
45767/239852450
# 40-49
37699/214196600
# 50-59
26932/195256750
# 60-69
15910/174159750
# 70-79
6777/94177300
# 80+
5022/43194100

# British Columbia
# <20
31358/365361350
# 20-29
32273/235063650
# 30-39
29060/258493000
# 40-49
22232/223380000
# 50-59
18612/241673800
# 60-69
12850/235727950
# 70-79
6437/157993900
# 80+
4479/76292300

# Ontario
# <20
91794/1154868640
# 20-29
```

```r
106911/708554560
# 30-39
83615/699667360
# 40-49
68257/631811840
# 50-59
65403/708856320
# 60-69
42221/628750080
# 70-79
20880/399589120
# 80+
16257/103681792

#### IRR <20
# IRR Alberta : British Columbia (ref)
res <- rateratio(58130, 31358, 397568950, 365361350, conf.level=0.95)
str(res)
print(res)

# IRR Ontario : British Columbia (ref)
res <- rateratio(91794, 31358, 1154868640, 365361350, conf.level=0.95)
str(res)
print(res)

#### IRR 20-29
# IRR Alberta : British Columbia (ref)
res <- rateratio(42204, 32273, 194986650, 235063650, conf.level=0.95)
str(res)
print(res)

# IRR Ontario : British Columbia (ref)
res <- rateratio(106911, 32273, 708554560, 235063650, conf.level=0.95)
str(res)
print(res)

#### IRR 30-39
# IRR Alberta : British Columbia (ref)
res <- rateratio(45767, 29060, 239852450, 258493000, conf.level=0.95)
str(res)
print(res)

# IRR Ontario : British Columbia (ref)
res <- rateratio(83615, 29060, 699667360, 258493000, conf.level=0.95)
str(res)
print(res)
```

```
#### IRR 40-49
# IRR Alberta : British Columbia (ref)
res <- rateratio(37699, 22232, 214196600, 223380000, conf.level=0.95)
str(res)
print(res)

# IRR Ontario : British Columbia (ref)
res <- rateratio(68257, 22232, 631811840, 223380000, conf.level=0.95)
str(res)
print(res)

#### IRR 50-59
# IRR Alberta : British Columbia (ref)
res <- rateratio(26932, 18612, 195256750, 241673800, conf.level=0.95)
str(res)
print(res)

# IRR Ontario : British Columbia (ref)
res <- rateratio(65403, 18612, 708856320, 241673800, conf.level=0.95)
str(res)
print(res)

#### IRR 60-69
# IRR Alberta : British Columbia (ref)
res <- rateratio(15910, 12850, 174159750, 235727950, conf.level=0.95)
str(res)
print(res)

# IRR Ontario : British Columbia (ref)
res <- rateratio(42221, 12850, 628750080, 235727950, conf.level=0.95)
str(res)
print(res)

#### IRR 70-79
# IRR Alberta : British Columbia (ref)
res <- rateratio(6777, 6437, 94177300, 157993900, conf.level=0.95)
str(res)
print(res)

# IRR Ontario : British Columbia (ref)
res <- rateratio(20880, 6437, 399589120, 157993900, conf.level=0.95)
str(res)
print(res)

#### IRR 80+
```

```r
# IRR Alberta : British Columbia (ref)
res <- rateratio(5022, 4479, 43194100, 76292300, conf.level=0.95)
str(res)
print(res)

# IRR Ontario : British Columbia (ref)
res <- rateratio(16257, 4479, 103681792, 76292300, conf.level=0.95)
str(res)
print(res)


#### Total (Female + Male) ####
#### Total (Age)
# Cumulative Case Count per person days
# Alberta
486747/2954006055
# British Columbia
319383/3650641670
# Ontario
1027756/10468821312

# IRR Total - Alberta : British Columbia (ref)
res <- rateratio(486747, 319383, 2954006055, 3650641670, conf.level=0.
95)
str(res)
print(res)

# IRR Total - Ontario : British Columbia (ref)
res <- rateratio(1027756, 319383, 10468821312, 3650641670, conf.level=
0.95)
str(res)
print(res)

#### Age Groups
# Cumulative Case Counts per person days
# Alberta
# <20
113044/734164200
# 20-29
88197/363277530
# 30-39
95877/458911530
# 40-49
78477/405709920
# 50-59
54151/372085560
```

```r
# 60-69
30851/334379430
# 70-79
13499/186697665
# 80+
12651/98780220

# British Columbia
# <20
60541/708530700
# 20-29
66127/461166550
# 30-39
60664/519712550
# 40-49
46963/459735750
# 50-59
37728/500301850
# 60-69
24791/492410550
# 70-79
12196/331011200
# 80+
10373/177773250

# Ontario
# <20
179796/2247188320
# 20-29
220159/1377056000
# 30-39
174988/1419920640
# 40-49
148854/1313568640
# 50-59
137198/1458332480
# 60-69
82601/1309929120
# 70-79
41258/687394560
# 80+
42902/393735712

#### IRR <20
# IRR Alberta : British Columbia (ref)
res <- rateratio(113044, 60541, 734164200, 708530700, conf.level=0.95)
```

```r
str(res)
print(res)

# IRR Ontario : British Columbia (ref)
res <- rateratio(179796, 60541, 2247188320, 708530700, conf.level=0.95
)
str(res)
print(res)

#### IRR 20-29
# IRR Alberta : British Columbia (ref)
res <- rateratio(88197, 66127, 363277530, 461166550, conf.level=0.95)
str(res)
print(res)

# IRR Ontario : British Columbia (ref)
res <- rateratio(220159, 66127, 1377056000, 461166550, conf.level=0.95
)
str(res)
print(res)

#### IRR 30-39
# IRR Alberta : British Columbia (ref)
res <- rateratio(95877, 60664, 458911530, 519712550, conf.level=0.95)
str(res)
print(res)

# IRR Ontario : British Columbia (ref)
res <- rateratio(174988, 60664, 1419920640, 519712550, conf.level=0.95
)
str(res)
print(res)

#### IRR 40-49
# IRR Alberta : British Columbia (ref)
res <- rateratio(78477, 46963, 405709920, 459735750, conf.level=0.95)
str(res)
print(res)

# IRR Ontario : British Columbia (ref)
res <- rateratio(148854, 46963, 1313568640, 459735750, conf.level=0.95
)
str(res)
print(res)

#### IRR 50-59
```

```r
# IRR Alberta : British Columbia (ref)
res <- rateratio(54151, 37728, 372085560, 500301850, conf.level=0.95)
str(res)
print(res)

# IRR Ontario : British Columbia (ref)
res <- rateratio(137198, 37728, 1458332480, 500301850, conf.level=0.95
)
str(res)
print(res)

#### IRR 60-69
# IRR Alberta : British Columbia (ref)
res <- rateratio(30851, 24791, 334379430, 492410550, conf.level=0.95)
str(res)
print(res)

# IRR Ontario : British Columbia (ref)
res <- rateratio(82601, 24791, 1309929120, 492410550, conf.level=0.95)
str(res)
print(res)

#### IRR 70-79
# IRR Alberta : British Columbia (ref)
res <- rateratio(13499, 12196, 186697665, 331011200, conf.level=0.95)
str(res)
print(res)

# IRR Ontario : British Columbia (ref)
res <- rateratio(41258, 12196, 687394560, 331011200, conf.level=0.95)
str(res)
print(res)

#### IRR 80+
# IRR Alberta : British Columbia (ref)
res <- rateratio(12651, 10373, 98780220, 177773250, conf.level=0.95)
str(res)
print(res)

# IRR Ontario : British Columbia (ref)
res <- rateratio(42902, 10373, 393735712, 177773250, conf.level=0.95)
str(res)
print(res)
```

The END of Appendix
